# New Abietane and Kaurane Type Diterpenoids from the Stems of *Tripterygium regelii*

**DOI:** 10.3390/ijms18010147

**Published:** 2017-01-13

**Authors:** Dongsheng Fan, Shuangyan Zhou, Zhiyuan Zheng, Guo-Yuan Zhu, Xiaojun Yao, Ming-Rong Yang, Zhi-Hong Jiang, Li-Ping Bai

**Affiliations:** 1 State Key Laboratory of Quality Research in Chinese Medicine, Macau Institute for Applied Research in Medicine and Health, Macau University of Science and Technology, Taipa, Macau 999078, China; fandongsheng1985@sina.com (D.F.); zyzheng@must.edu.mo (Z.Z.); gyzhu@must.edu.mo (G.-Y.Z.); xjyao@must.edu.mo (X.Y.); mryang@must.edu.mo (M.-R.Y.); zhjiang@must.edu.mo (Z.-H.J.); 2 State Key Laboratory of Applied Organic Chemistry and Department of Chemistry, Lanzhou University, Lanzhou 730000, China; zhoushy13@lzu.edu.cn

**Keywords:** *Tripterygium regelii*, diterpenoids, cytotoxicity

## Abstract

Eleven new abietane type (**1**‒**11**), and one new kaurane (**12**), diterpenes, together with eleven known compounds (**13**–**23**), were isolated and identified from the stems of *Tripterygium regelii*, which has been used as a traditional folk Chinese medicine for the treatment of rheumatoid arthritis in China. The structures of new compounds were characterized by means of the interpretation of high-resolution electrospray ionization mass spectrometry (HRESIMS), extensive nuclear magnetic resonance (NMR) spectroscopic data and comparisons of their experimental CD spectra with calculated electronic circular dichroism (ECD) spectra. Compound **1** is the first abietane type diterpene with an **18→1** lactone ring. Compound **19** was isolated from the plants of the *Tripterygium* genus for the first time, and compounds **14**–**17** were isolated from *T. regelii* for the first time. Triregelin I (**9**) showed significant cytotoxicity against A2780 and HepG2 with IC_50_ values of 5.88 and 11.74 µM, respectively. It was found that this compound was inactive against MCF-7 cells. The discovery of these twelve new diterpenes not only provided information on chemical substances of *T. regelii*, but also contributed to the chemical diversity of natural terpenoids.

## 1. Introduction

Diterpenes are naturally-occurring 20-carbon terpenoids that display a wide array of potentially useful biological effects. Abietanes are a large group of diterpenoids, which have been isolated from a variety of terrestrial plants, such as families of Araucariaceae, Cupressaceae, Phyllocladaceae, Pinaceae, Podocarpaceae, Asteraceae, Celastraceae, Hydrocharitaceae, and Lamiaceae, etc. [1,2]. Furthermore, this class of diterpenes have been found from fungal species [2]. So far, it has been reported that some abietane type diterpenes displayed a broad spectrum of promising biological activities including anticancer [3], cytotoxic [4,5], antiviral [6,7,8,9], anti-inflammatory [10], and anti-oxidant [9] effects, and so on. For example, tanshinone IIA was regarded as a potent cytotoxic compound for human leukemia cells [11]. Carnosol has been found to possess favorable anticancer and chemo-preventive effects [12]. Triptolide is a promising lead compound to treat inflammatory, immunological and cancerous diseases [13]. Recently, it was reported that miltirone is an inhibitor of P-glycoprotein [14].

**Figure 1:**
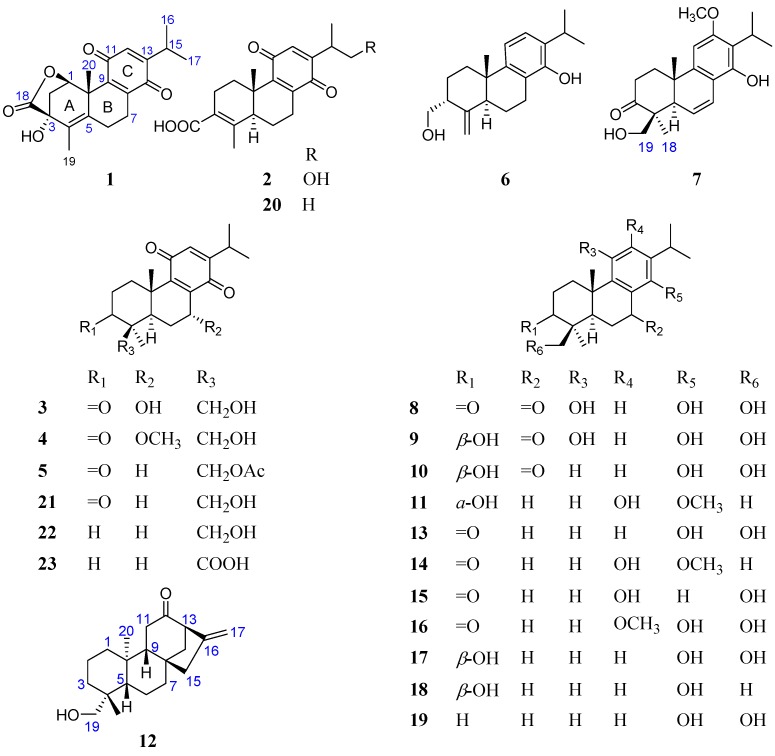
The chemical structures of compounds **1**−**23**.

As a part of ongoing research work on bioactive constituents from *Tripterygium regelii* [15,16,17], the methanolic extract of the stems of *T. regelii* was further investigated, leading to the isolation and characterization of twenty three diterpenoids, including eleven new abietane (**1**−**11**) and one new kaurane type (**12**) diterpenes, as well as eleven known abietane compounds (**13**−**23**) (Figure 1). Herein, this paper reports the isolation and structural elucidation of these new diterpenes, as well as cytotoxic evaluation of seventeen diterpenes on three cancer cell lines.

## 2. Results and Discussion

**Figure 2:**
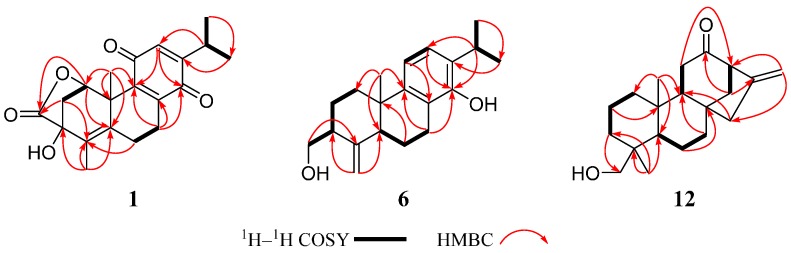
The ^1^H–^1^H COSY and key HMBC correlations of compounds **1**, **6**, and **12**.

Compound **1** was obtained as a yellow amorphous powder with a molecular formula of C_20_H_22_O_5_, which was determined by a protonated molecular ion at *m*/*z* 343.1546 [M + H]^+^ (calcd for C_20_H_23_O_5_, 343.1540) in its high-resolution electrospray ionization mass spectrometry (HRESIMS), indicating 10 degrees of unsaturation. IR spectrum of **1** showed a lactone carbonyl band at 1732 cm^−1^ and benzoquinone bands at 1680 and 1601 cm^−1^. The UV spectrum of **1** exhibited an absorption maximum at 260 nm, which is characteristic of a *p*-benzoquinone. The ^1^H nuclear magnetic resonance (NMR) spectroscopic data (Table 1) exhibited the characteristic signals for a benzoquinone proton (*δ*_H_ 6.41 (1H, d, *J* = 1.2 Hz, H-12)), an oxygenated methine (*δ*_H_ 5.86 (1H, d, *J* = 6.0 Hz, H-1)), an isopropyl moiety including a methine (*δ*_H_ 3.00 (1H, sept d, *J* = 6.6, 1.2 Hz, H-15)) and two secondary methyls (*δ*_H_ 1.12 and 1.11 (each 3H, d, *J* = 6.6 Hz, H_3_-16 and H_3_-17)), and two tertiary methyls (*δ*_H_ 1.85 and 1.54 (each 3H, s, H_3_-19 and H_3_-20)). The ^13^C NMR spectroscopic data (Table 2) displayed resonances for 20 carbons, which were confirmed by distortionless enhancement by polarization transfer (DEPT) and heteronuclear single quantum coherence (HSQC) experiments to be an ester carbonyl carbon (*δ*_C_ 177.3), a trisubstituted *p*-benzoquinone (*δ*_C_ 187.3 (C-11), 186.8 (C-14), 153.9 (C-13), 146.0 (C-8), 144.6 (C-9) and 131.6 (C-12)), a tetrasubstituted double bond (*δ*_C_ 132.6 (C-5) and 130.2 (C-4)), two aliphatic quaternary carbons (including an oxygenated one), two methines (including an oxygenated one), three methylenes, and four methyl groups. These spectroscopic data (Table 1 and Table 2) suggested that compound **1** is an abietane type diterpene with a *p*-benzoquinone C-ring [18,19], structurally similar to the known triptoquinone A (**20**) [18], an 18(4→3)-*abeo*-abietane quinone type diterpene, except for the A-ring. The Δ^4,5^ double bond was inferred from the HMBC correlations from H_2_-2, H_2_-6 and H_3_-19 to C-4 (*δ*_C_ 130.2), from H-7 (*δ*_H_ 2.90), H_3_-19 and H_3_-20 to C-5 (*δ*_C_ 132.6). The oxygenated methine (*δ*_H_ 5.86; *δ*_C_ 78.4) was assigned to C-1 based on the HMBC correlations from H-1 proton (*δ*_H_ 5.86) to C-2 (*δ*_C_ 39.1), C-3 (*δ*_C_ 74.3), C-5 (*δ*_C_ 132.6), C-9 (*δ*_C_ 144.6), C-10 (*δ*_C_ 44.7), and C-20 (*δ*_C_ 23.7). The key HMBC correlation from H-1 (*δ*_H_ 5.86) to C-18 (*δ*_C_ 177.3) suggested a lactone formed between C-1 and C-18, accounting for the remaining one degree of unsaturation. Hydroxylation of C-3 was inferred from the HMBC correlations from H-1 (*δ*_H_ 5.86), H_2_-2 (*δ*_H_ 2.36 and 1.96) and H_3_-19 (*δ*_H_ 1.85) to C-3 (*δ*_C_ 74.3). It was deduced that the proton at C-1 and the hydroxyl group at C-3 should be a *cis* relationship due to the lactone between C-1 and C-3. Therefore, the proposed structure of **1** was established as a lactone derivative of triptoquinone A bearing 5*S*, 10*S* absolute configuration by X-ray crystallographic analysis [18] (Figure 2).

**Figure 3:**
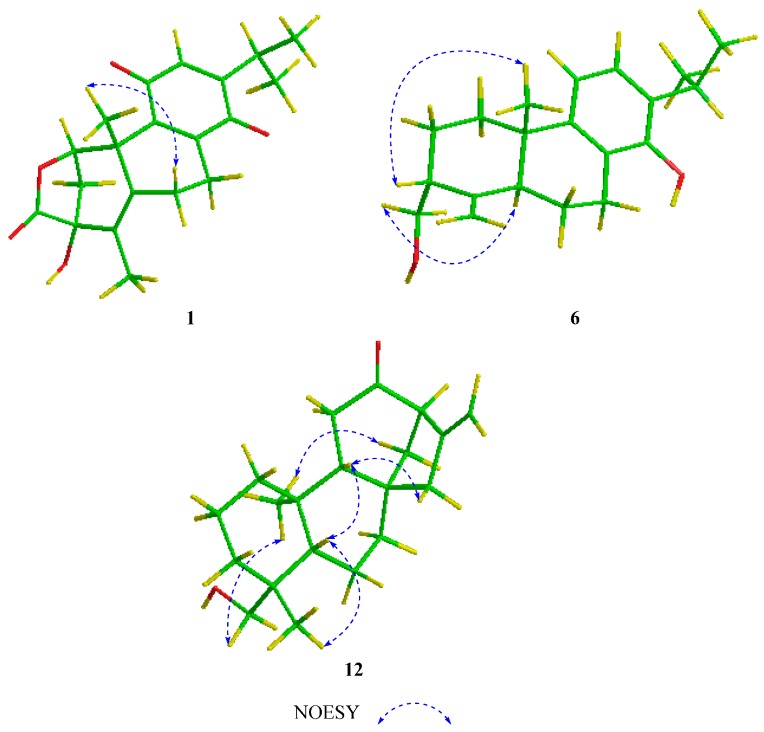
The selected NOESY correlations of compounds **1**, **6**, and **12**. The red, yellow and green atoms represent oxygens, hydrogens and carbons, respectively.

**Figure 4:**
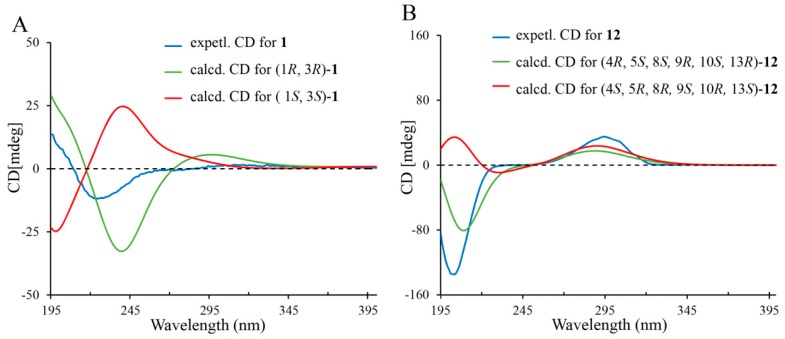
Experimental and calculated CD spectra of compounds **1** (**A**) and **12** (**B**).

However, the relative configuration of the substituents at the C-1 and C-3 could not be assigned by nuclear Overhauser effect spectroscopy (NOESY) experiment, owing to the fact that no any key NOE effects were observed (Figure 3). Hence, electron circular dichroism (ECD) calculations were conducted to determine the absolute configuration of compound **1** by time-dependent density functional theory (TDDFT) with the B3LYP/DGDZVP method [20,21]. The calculated ECD of (1*R*, 3*R*)-**1** matched well with the experimental CD spectrum (Figure 4A) of **1**. Therefore, compound **1** was determined as proposed, and given the trivial name of triregelin A.

Compound **2** was assigned as a molecular formula of C_20_H_24_O_5_ based on a deprotonated molecular ion at *m*/*z* 343.1560 [M − H]^−^ (calcd for C_20_H_23_O_5_, 343.1551) in its HRESIMS. IR spectrum of **2** exhibited a conjugated carboxylic acid band at 1688 cm^−1^ and benzoquinone band at 1649 cm^−1^. The ^1^H and ^13^C NMR spectroscopic data (Table 1 and Table 2) of **2** were closely analogous to those of triptoquinone A (**20**) [18], except for the absence of a secondary methyl group and the presence of a hydroxymethyl group (*δ*_H_ 3.67 (2H, d, *J* = 7.2 Hz, H_2_-17); *δ*_C_ 66.6). The hydroxymethyl group was allocated to be at C-15, as deduced by ^1^H–^1^H COSY correlation of H-15/H_2_-17 and HMBC correlations from the hydroxymethyl protons to C-13 (*δ*_C_ 149.0) and C-15 (*δ*_C_ 34.5). Therefore, compound **2** was characterized and given a trivial name of triregelin B.

Compound **3** showed a molecular formula of C_20_H_26_O_5_, as established from an [M + H]^+^ ion at *m*/*z* 347.1866 (calcd C_20_H_27_O_5_, 347.1853) in the HRESIMS. Analysis of the NMR spectroscopic data (Table 1 and Table 2) indicated that **3** was structurally related to triptoquinone B (**21**) [18] except for the absence of the C-7 methylene in triptoquinone B and the presence of an additional hydroxyl proton (*δ*_H_ 2.77) and an oxygenated methine (*δ*_H_ 4.81, *δ*_C_ 61.9) in **3**. These data suggested hydroxylation of C-7 in **3**, which was supported by HMBC correlation from the hydroxyl proton (*δ*_H_ 2.77) to C-7 (*δ*_C_ 61.9). The α-orientation of the hydroxyl group at C-7 was deduced from the NOESY correlation of H-7/H_3_-20. Thus, compound **3** was identified and named triregelin C.

Compound **4** gave a molecular formula of C_21_H_28_O_5_, as deduced from an [M + H]^+^ ion at *m*/*z* 361.2008 (calcd C_21_H_29_O_5_, 361.2010), 14.0142 atomic mass units (amu) more than that of **3** in the HRESIMS. The ^1^H and ^13^C NMR spectroscopic data (Table 1 and Table 2) of **4** were closely similar to those of **3**, except for the appearance of a methoxyl group. The methoxyl group was assigned at C-7, as evidenced from the observed HMBC correlation from the methoxyl protons (*δ*_H_ 3.50) to C-7 (*δ*_C_ 69.8). Thus, compound **4** was characterized and named triregelin D.

Compound **5** showed a molecular formula of C_22_H_28_O_5_ on the basis of a protonated molecular ion at *m*/*z* 373.1998 [M + H]^+^ (calcd C_22_H_29_O_5_, 373.2010) in its HRESIMS, 42.0106 amu more than that of triptoquinone B (**21**) [18]. The 1D NMR spectroscopic data (Table 1 and Table 2) of **5** were analogous to those of triptoquinone B (**21**) [18], except for the presence of an acetyl group (*δ*_H_ 2.03; *δ*_C_ 170.8, 20.9). The acetyl group was allocated to C-19, as evidenced from the HMBC correlations from H_2_-19 (*δ*_H_ 4.56, 4.08) to the carbonyl carbon (*δ*_C_ 170.8) of the acetyl group. Therefore, compound **5** was determined and named triregelin E.

Compound **6** had a molecular formula of C_20_H_28_O_2_ deduced from a protonated molecular ion at *m*/*z* 301.2162 [M + H]^+^ (calcd for C_20_H_29_O_2_, 301.2162) in the HRESIMS. IR spectrum of **6** displayed a double bond band at 1626 cm^−1^, and aromatic ring bands at 1580 and 1424 cm^−1^. The ^1^H NMR data (Table 3) showed the characteristic signals for two coupled aromatic protons (*δ*_H_ 7.04 and 6.09 (1H each, d, *J* = 7.8 Hz)), two singlet vinylic protons (*δ*_H_ 4.86 and 4.77), an oxygenated methylene (*δ*_H_ 3.95 and 3.70), an isopropyl moiety (*δ*_H_ 3.15, 1.26 and 1.25), and a tertiary methyl group (*δ*_H_ 0.99). The ^13^C NMR data (Table 2) displayed resonances for 20 carbons, which were ascribed to a tetrasubstituted benzene ring, an exocyclic double bond, an aliphatic quaternary carbon, three methines, five methylenes (including an oxygenated one) and three methyl groups. The ^1^H and ^13^C NMR spectroscopic data of **6** were similar to those of triptobenzene P [22], an 18 (4→3)-*abeo*-abietane diterpene previously isolated from *T. wilfordii*, except for the following two differences. One difference is the replacement of the methoxyl group at C-12 in triptobenzene P by a hydrogen in **6**, which was supported by ^1^H–^1^H COSY correlation of H-11/H-12, and HMBC correlations from H-12 (*δ*_H_ 7.04) to C-9 (*δ*_C_ 145.8) and C-15 (*δ*_C_ 26.9). The other difference is the downfield shift of C-14 (*δ*_C_ 150.3) in **6** relative to that (*δ*_C_ 123.8) in triptobenzene P, indicating hydroxylation of C-14 in **6**. Thus, the planar structure of **6** was established as 12-demethoxy-14-hydroxy-triptobenzene P, which was confirmed by the ^1^H–^1^H COSY and HMBC data (Figure 2). The NOE correlations of H-5α/H_2_-18 and H_3_-20*β*/H-3 indicated *α*-orientation of CH_2_-18 (Figure 3). Thus, compound **6** was defined and named triregelin F.

Compound **7** gave a molecular formula of C_21_H_28_O_4_, as established from an [M + H]^+^ ion at *m*/*z* 345.2055 (calcd for C_21_H_29_O_4_, 345.2060) in the HRESIMS. IR spectrum of **7** exhibited a carbonyl band at 1703 cm^−1^, and aromatic ring bands at 1604, 1566 and 1455 cm^−1^. The ^1^H and ^13^C NMR spectroscopic data (Table 2 and Table 3) of **7** were very similar to those of triptobenzene A (**13**) [23], except for the absence of two methylene groups and an aromatic proton, and the presence of a double bond (*δ*_H_ 6.82 (1H, dd, *J* = 10.2, 3.0 Hz, H-7); 122.1 and 5.86 (1H, dd, *J* = 10.2, 3.0 Hz, H-6); *δ*_C_ 124.0), and a methoxyl group (*δ*_H_ 3.80 (3H,s, OCH_3_-12); *δ*_C_ 55.7). The double bond was assigned at between C-6 and C-7, which was supported by HMBC correlations from H-6 (*δ*_H_ 5.86) to C-4 (*δ*_C_ 53.0) and C-10 (*δ*_C_ 37.7), and from H-7 (*δ*_H_ 6.82) to C-9 (*δ*_C_ 145.2) and C-14 (*δ*_C_ 150.3). The methoxyl group was located at C-12, as deduced from the HMBC correlation from the methoxyl protons (*δ*_H_ 3.80) to C-12 (*δ*_C_ 158.3). The key NOE correlations of H-5*α*/H_3_-18 and H_3_-20/H_2_-19 were observed in the NOESY spectrum. Accordingly, compound **7** was elucidated as illustrated in Figure 1, and named triregelin G.

Compound **8** had a molecular formula of C_20_H_26_O_5_, according to an [M + H]^+^ ion at *m*/*z* 347.1840 [M + H]^+^ (calcd for C_20_H_27_O_5_, 347.1853). The ^1^H and ^13^ C NMR spectroscopic data (Table 2 and Table 3) of **8** were closely related to those of triptobenzene A (**13**) [23]. However, one of the key differences was the replacement of the methylene at C-7 in triptobenzene A by a keto carbonyl carbon (*δ*_C_ 204.3) in **8**, as evidenced from HMBC correlation from H-5 (*δ*_H_ 2.64) to C-7. The other difference was the absence of a doublet aromatic proton and the presence of an additional hydroxyl proton (*δ*_H_ 4.62) together with the downfield shift of C-11 (*δ*_C_ 144.5) in **8** compared to that in triptobenzene A, which suggested hydroxylation of C-11 in **8**. Therefore, compound **8** was assigned and named triregelin H.

Compound **9** had a molecular formula of C_20_H_28_O_5_ based on a protonated molecular ion at *m*/*z* 349.2005 [M + H]^+^ (calcd for C_20_H_29_O_5_, 349.2010), with 2.0161 amu more than that of **8** in the HRESIMS. The ^13^C NMR spectroscopic data (Table 2) of **9** were closely comparable to those of **8**, except for the absence of the C-3 keto carbonyl in **8**, and the presence of an oxygenated methine (*δ*_H_ 3.56 (1H, dd, *J* = 11.4, 3.6 Hz, H-3); *δ*_C_ 79.7) in **9**. These suggested that the C-3 keto carbonyl group in **8** was reduced to be a hydroxyl group in **9**. The hydroxyl group at C-3 was β-oriented, as deduced from the NOESY correlations of H-3/H-5*α* and H-3/H_3_-18. Therefore, compound **9** was identified and named triregelin I.

Compound **10** displayed a molecular formula of C_20_H_28_O_4_ established by a protonated molecular ion at *m*/*z* 333.2053 [M + H]^+^ (calcd for C_20_H_29_O_4_, 333.2060), revealing 15.9948 amu less than that in **9** in the HRESIMS. The ^1^H and ^13^C NMR spectroscopic data (Table 2 and Table 3) of **10** were very similar to those of **9** except for the presence of an extra doublet aromatic proton (*δ*_H_ 6.74, H-11) and the upfield shift of C-11 (*δ*_C_ 113.6) relative to that (*δ*_C_ 144.2) in **9**. These data revealed the dehydroxylation of C-11 in **10**, which was further supported by ^1^H–^1^H COSY correlation of H-11/H-12, and HMBC correlations from H-11 to C-8 (*δ*_C_ 114.2), C-10 (*δ*_C_ 37.5), and C-13 (*δ*_C_ 134.9). Hence, compound **10** was elucidated and named triregelin J.

Compound **11** showed a molecular formula of C_21_H_32_O_3_, as deduced from a protonated molecular ion at *m*/*z* 333.2426 [M + H]^+^ (calcd for C_21_H_33_O_3_, 333.2424) in the HRESIMS. Comparison of the NMR spectroscopic data (Table 2 and Table 3) of **11** with neotriptonoterpene (**14**) [24] showed that both compounds were structurally comparable, except for the absence of the C-3 keto carbonyl in neotriptonoterpene (**14**) and the presence of an extra oxygenated methine (*δ*_H_ 3.68; *δ*_C_ 74.8) in **11**. These suggested that the C-3 keto carbonyl group in neotriptonoterpene (**14**) was reduced to be a hydroxyl group in **11**. The C-3 hydroxyl group was α-oriented, as inferred from the coupling constant (*J*_2,3_ = 3.6 Hz) and the NOESY correlation between H-3 and H_3_-19. Accordingly, the compound **11** was characterized and named triregelin K.

Compound **12**, white amorphous power, had a molecular formula of C_20_H_30_O_2_, as deduced from an [M + H]^+^ ion at *m*/*z* 303.2322 (calcd for C_20_H_31_O_2_, 303.2319) in the HRESIMS. IR spectrum of **12** exhibited a strong carbonyl band at 1710 cm^−1^. The ^1^H NMR spectrum (Table 1) exhibited the characteristic signals for a vinylic group (*δ*_H_ 4.99 and 4.87), an oxygenated methylene (*δ*_H_ 3.68 and 3.44), a hydroxyl group (*δ*_H_ 1.09), and two tertiary methyls (*δ*_H_ 0.98 and 0.85). The ^13^C NMR and DEPT spectra (Table 2) showed 20 carbon signals including a carbonyl group, an exocyclic double bond, three quaternary carbons, there methines, nine methylenes (including an oxygenated one) and two methyl groups. All the above NMR data indicated that **12** was a kaurane type diterpenoid, and structurally similar to (−)-*ent*-kaur-16-en-19-ol [25,26,27]. The distinct difference was that the C-12 methylene in (−)-*ent*-kaur-16-en-19-ol was oxidized to be a keto carbonyl group in **12**, as deduced from the downfield shift of C-12 (δ_C_ 211.5), and the HMBC correlations from H-9 (*δ*_C_ 1.58) and H_2_-14 (*δ*_H_ 2.40, 1.51) to C-12. Finally, the planar structure of **12** was confirmed on the basis of the ^1^H–^1^H COSY and HMBC experiments (Figure 2). In the NOESY spectrum, the correlations of H_3_-20/H_2_-19 and H_3_-20/H_2_-14 indicated that these protons were in the same face. In the same way, the other key NOE cross peaks of H-5/H-9 and H-9/H_2_-15 were also observed (Figure 3), suggesting H-5, H-9, and H_2_-15 were in the other face. However, **12** displayed a positive specific rotation (${\left[\alpha \right]}_{D}^{21}$ +50.86 (*c* 0.50, MeOH)) in contrast to the negative one reported for (−)-*ent*-kaur-16-en-19-ol [27]. ECD curves for the two possible stereo-structures (4*R*, 5*S*, 8*S*, 9*R*, 10*S*, 13*R*-**12** and 4*S*, 5*R*, 8*R*, 9*S*, 10*R*, 13*S*-**12**) were, therefore, calculated to determine the absolute configuration of **12**. As illustrated in Figure 4B, the calculated profile of 4*R*, 5*S*, 8*S*, 9*R*, 10*S*, and 13*R*-**12** were in good agreement with the experimental CD spectrum of **12**. Therefore, compound **12** was identified and named triregelin L.

The compounds **3**, **6**, **7**, and **9** were also selected to calculate their ECD data in order to further confirm their absolute configurations. As the results, their experimental CD spectra showed similar CD pattern to the calculated ones of (4*S*, 5*R*, 7*R*, 10*S*)-**3**, (3*R*, 5*S*, 10*S*)-**6**, (4*S*, 5*R*, 10*S*)-**7,** and (3*S*, 4*S*, 5*R*, 10*S*)-**9**, respectively (Figure S1). The HRMS, UV, IR, NMR and CD spectra (Figures S2–S121) of twelve new compounds were shown in supplementary materials.

In addition, eleven known abietanes were also isolated from the stems of *T. regelii*, including triptobenzene A (**13**) [23], neotriptonoterpene (**14**) [24], triptobenzene M (**15**) [28], wilforol F (**16**) [29], triptobenzene J (**17**) [30], triptobenzene B (**18**) [23], abieta-8, 11, 13-triene-14, 19-diol (**19**) [31], triptoquinone A (**20**), triptoquinone B (**21**), triptoquinone D (**22**) and triptoquinone F (**23**) [18]. These compounds were identified by comparison of their spectroscopic (1D NMR and specific rotation) and HRMS data with those reported in the literature. Compound **1** is the first abietane type diterpene with an 18→1 lactone ring. The discovery of the above twelve new diterpenes contributed to the chemical diversity of natural terpenoids.

Cytotoxic effects of seventeen diterpenes (**2**, **7**–**11**, **13**–**23**) were evaluated against three cancer cell lines of A2780, HepG2 and MCF-7. As the results show (Table 4), compound **9** displayed cytotoxicity against A2780, HepG2, and MCF-7 cells with IC_50_ values of 5.88, 11.74, and 46.40 µM, respectively. Compound **11** showed solely cytotoxic effect on MCF-7 cell with an IC_50_ value of 26.70 µM. Compound **14** exhibited weak cytotoxic activity on A2780, HepG2, and MCF-7 cells with IC_50_ values of 65.80, 35.45, and 64.80 µM, respectively.

## 3. Materials and Methods

### 3.1. General Experimental Procedures

Optical rotations were obtained using a Rudolph Research Analytical Autopol I automatic polarimeter (Rudolph Research Analytical, Hackettstown, NJ, USA). IR spectra were measured on an Agilent Cary 600 series FT-IR spectrometer (KBr) (Agilent, Santa Clara, CA, USA). Ultraviolet (UV) spectra were recorded on a Beckman Coulter DU^®^ 800 spectrophotometer (Beckman Coulter, Fullerton, CA, USA). HRMS spectra were carried out on an Agilent 6230 electrospray ionization (ESI) time-of-flight (TOF) mass spectrometer (Agilent, Santa Clara, CA, USA). Nuclear magnetic resonance (NMR) spectra were measured on a Bruker Ascend 600 NMR spectrometer at 600 MHz for ^1^H NMR and 150 MHz for ^13^C NMR (Bruker, Zurich, Switzerland). Chemical shifts were expressed in *δ* (ppm) with tetramethylsilane (TMS) as an internal reference, and coupling constants (*J*) were reported in hertz (Hz). Circular dichroism spectra were measured on a Jasco J1500 CD spectrometer (Jasco Corporation, Tokyo, Japan). Medium pressure liquid chromatography (MPLC) was conducted on a Sepacore Flash Chromatography System (Buchi, Flawil, Switzerland) by employing a flash column (460 mm × 36 mm, i.d., Buchi) packed with Bondapak Waters ODS (40–63 μm, Waters, Milford, MA, USA). Preparative high performance liquid chromatography (HPLC) was carried out on a Waters Xbridge Prep C_8_ column (10 mm × 250 mm, 5 μm) by utilizing a Waters liquid chromatography system equipped with 1525 Binary HPLC Pump and 2489 UV/Visible detector (Waters, Milford, MA, USA). Semi-preparative HPLC was done on a Waters Xbridge Prep C_18_ column (10 mm × 250 mm, 5 μm) by using an Agilent 1100 liquid chromatography system coupled with a quaternary pump and a diode array detector (DAD) (Agilent, Santa Clara, CA, USA). Column chromatography was conducted on silica gel (40−60 μm, Grace, Columbia, MD, USA) and Bondapak Waters ODS (40–63 μm, Waters). Thin layer chromatographies (TLCs) were performed on pre-coated silica gel 60 F_254_ plates and TLC silica gel 60 RP-18 F_254S_ plates (200 µm thick, Merck KGaA, Darmstadt, Germany), which were used to monitor fractions. Spots on the TLC were visualized by UV light (254 nm) or heating after spraying with 5% H_2_SO_4_ in ethanol.

### 3.2. Plant Material

The stems of *T. regelii* used in this study were collected from Changbai Mountain in Jilin province, China, in October 2012. The plant was authenticated by Liang Xu, Liaoning University of Traditional Chinese Medicine (Dalian, China). A voucher specimen (No. MUST-TR201210) has been deposited at State Key Laboratory of Quality Research in Chinese Medicine, Macau University of Science and Technology.

### 3.3. Extraction and Isolation

The air-dried stems of *T. regelii* (8.0 kg) were powdered, and extracted three times with methanol (64 L) under ultrasonic-assisted extraction at room temperature for 1 h. The methanol extract was evaporated under reduced pressure to yield a dark brown residue, which was then suspended in H_2_O, and successively partitioned with *n*-hexane, ethyl acetate (EtOAc) and *n*-butanol. Then, the EtOAc-soluble extract (150.0 g) was fractionated over a silica gel column using a gradient system of petroleum ether (PE)-acetone (100:0–35:65, *v*/*v*) to provide thirteen fractions (Fr.1–Fr.13). Fraction 5 was chromatographed over an ODS column using a gradient system of MeOH–H_2_O (50:50–100:0, *v*/*v*) to yield eight fractions (Fr.5-1–Fr.5-8). Fractions 5-2 (110.0 mg) and 5-4 (130.0 mg) were further separated on silica gel columns eluted with PE–EtOAc (95:5–55:45, *v*/*v*) to afford compounds **22** (15.0 mg) and **23** (30.0 mg), respectively. Similarly, fraction 7 (5.0 g) was subjected to an ODS column with a gradient system of MeOH–H_2_O (40:60–90:10, *v*/*v*) to give nine fractions (Fr.7-1–Fr.7-9). Compounds **20** (50.0 mg) and **21** (30.0 mg) were obtained by silica gel columns separation using PE–EtOAc (90:10–30:70, *v*/*v*) as eluting solvents from fractions 7-2 (150.0 mg) and 7-4 (100.0 mg), respectively. Fraction 8 (5.4 g) was subjected to an ODS column using a gradient system of MeOH–H_2_O (35:65–80:20, *v*/*v*) to afford ten fractions (Fr.8-1–Fr.8-10). The fraction 8-3 (2.5 g) was fractionated over a silica gel column, with a gradient elution by PE–EtOAc (90:10–63:35, *v*/*v*), to produce seven fractions (Fr.8-3-1–Fr.8-3-7). Fraction 8-3-4 (40.5 mg) was purified by semi-preparative HPLC using CH_3_CN–H_2_O (52:48, *v*/*v*) as mobile phase to afford compounds **5** (0.6 mg), **12** (0.7 mg) and **18** (1.0 mg). Fraction 8-3-5 (200.6 mg) was subjected to semi-preparative HPLC using CH_3_CN-H_2_O (58:42, *v*/*v*) as solvent system to give compounds **6** (1.9 mg) and **11** (2.0 mg), as well as subfraction 8-3-5-2. Compounds **14** (1.6 mg) and **19** (1.0 mg) were obtained by preparative HPLC with an isocratic elution of CH_3_CN-H_2_O (55:45, *v*/*v)* from the subfraction 8-3-5-2 (65.7 mg). Fraction 11 (5.5 g) was chromatographed over an ODS column, eluted with MeOH–H_2_O (30:70-100:0, *v*/*v*), to afford sixteen fractions (Fr.11-1–Fr.11-16). Compound **3** (5.0 mg) was isolated by preparative HPLC eluting with a MeOH–H_2_O (35:65, *v*/*v*) solvent system from fraction 11-4 (26.9 mg). Fraction 11-6 (49.5 mg) was separated by semi-preparative HPLC using CH_3_CN–H_2_O (35:65, v/v) as mobile phase to yield compound **2** (5.0 mg). Fraction 11-7 (261.1 mg) was subjected to a silica gel column with a gradient elution of PE–EtOAc (20:80–10:90, *v*/*v*) and purified by semi-preparative HPLC using CH_3_CN–H_2_O (40:60, *v*/*v*) as eluting solvent to afford compounds **8** (1.2 mg), **4** (2.0 mg), **13** (1.6 mg) and fraction 11-7-3. Compounds **7** (0.6 mg) and **16** (3.1 mg) were purified by semi-preparative HPLC with a CH_3_CN–H_2_O (42:58, *v*/*v*) solvent system from fraction 11-7-3 (56.9 mg). Fraction 11-8 (284.3 mg) was isolated by preparative HPLC using MeOH−H_2_O (46:54, *v*/*v*) as mobile phase to yield compound **1** (0.59 mg) and five fractions (Fr.11-8-1−Fr.11-8-5). Fraction 11-8-2 (42.5 mg) was subjected to semi-preparative HPLC with a CH_3_CN–H_2_O (47:53, *v*/*v*) solvent system to give compound **15** (2.0 mg). Fraction 11-8-3 (30.3 mg) was isolated by semi-preparative HPLC using CH_3_CN–H_2_O (45:55, *v*/*v*) as eluting solvent to yield compounds **10** (1.6 mg) and **17** (2.1 mg). Fraction 12 (9.0 g) was separated by MPLC using a gradient system of MeOH–H_2_O (5:95–100:0, 50 mL/min) to obtain six fractions (Fr.12-1–Fr.12-6). Fraction 12-5 was chromatographed over a silica gel column using CHCl_3_–MeOH (100:0–90:10, *v*/*v*) as solvent system, and then purified by semi-preparative HPLC using CH_3_CN–H_2_O (39:61, *v*/*v*) as mobile phase to give compound **9** (1.2 mg).

### 3.4. Structural Characterization

Triregelin A (**1**): yellow amorphous powder; ${\left[\alpha \right]}_{D}^{21}$ + 3.8 (*c* 0.50, MeOH); IR (KBr) *ν*_max_: 3444, 2925, 2854, 1732, 1601, 1455, 1377, 1260, 1167, 1086, 1013, 957, 893, 803, 756, 667 cm^−1^; UV (MeOH) *λ*_max_ (log *ε*) 260 (2.35) nm; CD (*c* 2.92 × 10^−3^ mol/L, MeOH) *λ*_max_ (Δ*ε*) 225 (−1.22), 311 (+0.16); ^1^H NMR (CDCl_3_, 600 MHz) and ^13^C NMR (CDCl_3_, 150 MHz) data, see Table 1 and Table 2; HRESIMS *m*/*z* 343.1546 [M + H]^+^ (calcd for C_20_H_23_O_5_, 343.1540).

Triregelin B (**2**): yellow amorphous powder; ${\left[\alpha \right]}_{D}^{21}$ + 75.5 (*c* 1.00, MeOH); IR (KBr) *ν*_max_: 3421, 2970, 2937, 2881, 1688, 1649, 1436, 1375, 1248, 1104, 1030, 977, 905, 798, 659, 599 cm^−1^; UV (MeOH) *λ*_max_ (log *ε*) 258 (3.17) nm; CD (*c* 1.46 × 10^−3^ mol/L, MeOH) *λ*_max_ (Δ*ε*) 269 (+7.24), 355 (+0.35), 476 (−0.54); ^1^H NMR (CDCl_3_, 600 MHz) and ^13^C NMR (CDCl_3_, 150 MHz) data, see Table 1 and Table 2; HRESIMS *m*/*z* 343.1560 [M − H]^−^ (calcd for C_20_H_23_O_5_, 343.1551).

Triregelin C (**3**): yellow, amorphous powder; ${\left[\alpha \right]}_{D}^{21}$ − 34.0 (*c* 1.00, MeOH); IR (KBr) *ν*_max_: 3437, 2966, 2936, 2876, 1700, 1650, 1463, 1430, 1383, 1294, 1235, 1165,1107, 1044, 974, 933, 900, 813, 741 cm^−1^; UV (MeOH) *λ*_max_ (log *ε*) 256 (3.42) nm; CD (*c* 1.45 × 10^−3^ mol/L, MeOH) *λ*_max_ (Δ*ε*) 283 (+2.21), 359 (−0.25), 478 (−0.32); ^1^H NMR (CDCl_3_, 600 MHz) and ^13^C NMR (CDCl_3_, 150 MHz,) data, see Table 1 and Table 2; HRESIMS *m*/*z* 347.1866 [M + H]^+^ (calcd for C_20_H_27_O_5_, 347.1853).

Triregelin D (**4**): yellow, amorphous powder; ${\left[\alpha \right]}_{D}^{21}$ − 9.3 (*c* 1.00, MeOH); IR (KBr) *ν*_max_: 3435, 2964, 2932, 2877, 1688, 1652, 1606, 1462, 1426, 1383, 1293, 1234, 1192, 1085, 1039, 910, 856 cm^−1^; UV (MeOH) *λ*_max_ (log *ε*) 255 (3.19) nm; CD (*c* 1.39 × 10^−3^ mol/L, MeOH) *λ*_max_ (Δ*ε*) 271 (+3.05), 357 (−0.22), 481 (−0.61); ^1^H NMR (CDCl_3_, 600 MHz) and ^13^C NMR (CDCl_3_, 150 MHz) data, see Table 1 and Table 2; HRESIMS *m*/*z* 361.2008 [M + H]^+^ (calcd for C_21_H_29_O_5_, 361.2010).

Triregelin E (**5**): yellow, amorphous powder; ${\left[\alpha \right]}_{D}^{21}$ + 17.0 (*c* 0.50, MeOH); IR (KBr) *ν*_max_: 3455, 2962, 2930, 2874, 1744, 1713, 1650, 1604, 1464, 1384, 1294, 1233, 1104, 1042, 906, 802, 757, 666, 603 cm^−1^; UV (MeOH) *λ*_max_ (log *ε*) 257 (2.75) nm; CD (*c* 1.34 × 10^−3^ mol/L, MeOH) *λ*_max_ (Δ*ε*) 261 (+3.35), 349 (+0.33), 474 (−0.30); ^1^H NMR (CDCl_3_, 600 MHz) and ^13^C NMR (CDCl_3_, 150 MHz) data, see Table 1 and Table 2; HRESIMS *m*/*z* 373.1998 [M + H]^+^ (calcd for C_22_H_29_O_5_, 373.2010).

Triregelin F (**6**): yellow, amorphous powder; ${\left[\alpha \right]}_{D}^{21}$+169.3 (*c* 1.00, MeOH); IR (KBr) *ν*_max_: 3437, 2964, 2872, 1626, 1424, 1250, 1160, 1115, 1059, 896, 815, 705 cm^-1^; UV (MeOH) *λ*_max_ (log *ε*) 223 (3.03), 270 (2.35) nm; CD (*c* 1.67 × 10^−3^ mol/L, MeOH) *λ*_max_ (Δ*ε*) 209 (+5.51), 217 (sh) (+3.41), 265 (+1.02); ^1^H NMR (CDCl_3_, 600 MHz) and ^13^C NMR (CDCl_3_, 150 MHz) data, see Table 2 and Table 3; HRESIMS *m*/*z* 301.2162 [M + H]^+^(calcd for C_20_H_29_O_2_, 301.2162).

Triregelin G (**7**): yellow, amorphous powder; ${\left[\alpha \right]}_{D}^{21}$ − 46.9 (*c* 0.50, MeOH); IR (KBr) *ν*_max_: 3382, 2958, 2929, 2872, 1703, 1604, 1566, 1455, 1417, 1378, 1312, 1261, 1224, 1137, 1107, 1056, 802, 756, 667 cm^−1^; UV (MeOH) *λ*_max_ (log *ε*) 233 (3.23), 279 (2.37), 312 (2.45) nm; CD (*c* 1.45 × 10^−^^3^ mol/L, MeOH) *λ*_max_ (Δ*ε*) 239 (−2.50), 286 (+1.08), 311 (−1.02), 392 (+0.31); ^1^H NMR (CDCl_3_, 600 MHz) and ^13^C NMR (CDCl_3_, 150 MHz) data, see Table 2 and Table 3; HRESIMS *m*/*z* 345.2055 [M + H]^+^ (calcd for C_21_H_29_O_4_, 345.2060).

Triregelin H (**8**): yellow, amorphous powder; ${\left[\alpha \right]}_{D}^{21}$ + 56.4 (*c* 1.00, MeOH); IR (KBr) *ν*_max_: 3398, 2961, 2926, 2872, 1697, 1624, 1429, 1382, 1349, 1301, 1233, 1162, 1107, 1040, 964, 894, 801, 754 cm^−1^; UV (MeOH) *λ*_max_ (log *ε*) 238 (2.82), 270 (2.38) nm; CD (*c* 1.45 × 10^−^^3^ mol/L, MeOH) *λ*_max_ (Δ*ε*) 233 (+2.77), 270 (−0.27), 310 (−1.08), 377 (+1.25); ^1^H NMR (CDCl_3_, 600 MHz) and ^13^C NMR (CDCl_3_, 150 MHz) data, see Table 2 and Table 3; HRESIMS *m*/*z* 347.1840 [M + H]^+^ (calcd for C_20_H_27_O_5_, 347.1853).

Triregelin I (**9**): white, amorphous powder; ${\left[\alpha \right]}_{D}^{21}$ + 29.4 (*c* 0.50, MeOH); IR (KBr) *ν*_max_: 3396, 2959, 2924, 2854, 1714, 1592, 1428, 1348, 1260, 1168, 1114, 1028, 970, 800, 755, 709 cm^−1^; UV (MeOH) *λ*_max_ (log *ε*) 235 (3.32), 270 (3.22), 380 (2.95) nm; CD (*c* 1.44 × 10^−^^3^ mol/L, MeOH) *λ*_max_ (Δ*ε*) 206 (+2.23), 235 (+0.88), 271 (−0.65), 311 (−0.61), 375 (+0.96); ^1^H NMR (CDCl_3_, 600 MHz) and ^13^C NMR (CDCl_3_, 150 MHz) data, see Table 2 and Table 3; HRESIMS *m*/*z* 349.2005 [M + H]^+^ (calcd for C_20_H_29_O_5_, 349.2010).

Triregelin J (**10**): white, amorphous powder; ${\left[\alpha \right]}_{D}^{21}$ + 1.4 (*c* 1.00, MeOH); IR (KBr) *ν*_max_: 3364, 2962, 2937, 2870, 1622, 1558, 1455, 1427, 1381, 1347, 1251, 1212, 1160, 1113, 1080, 1037, 981, 914, 821, 757, 711, 663, 582 cm^−1^; UV (MeOH) *λ*_max_ (log *ε*) 216 (3.40), 266 (2.33), 343 (2.42) nm; CD (*c* 1.51 × 10^−3^ mol/L, MeOH) *λ*_max_ (Δ*ε*) 218 (+1.82), 230 (+1.34), 266 (−3.14), 333 (+1.23), 346 (+1.71); ^1^H NMR (CDCl_3_, 600 MHz) and ^13^C NMR (CDCl_3_, 150 MHz) data, see Table 2 and Table 3; HRESIMS *m*/*z* 333.2053 [M + H]^+^ (calcd for C_20_H_29_O_4_, 333.2060).

Triregelin K (**11**): white, amorphous powder; ${\left[\alpha \right]}_{D}^{21}$ + 28.1 (*c* 0.25, MeOH); IR (KBr) *ν*_max_: 3396, 3210, 2926, 2865, 1737, 1607, 1581, 1441, 1412, 1373, 1331, 1308, 1268, 1206, 1101, 1041, 942, 926, 857, 800, 756, 695, 660 cm^−1^; UV (MeOH) *λ*_max_ (log *ε*) 227 (2.82), 283 (2.36) nm; CD (*c* 1.51 × 10^−3^ mol/L, MeOH) *λ*_max_ (Δ*ε*) 228 (+0.97); ^1^H NMR (pyridine-*d*_5_, 600 MHz) and ^13^C NMR (pyridine-*d*_5_, 150 MHz) data, see Table 2 and Table 3; HRESIMS *m*/*z* 333.2426 [M + H]^+^ (calcd for C_21_H_33_O_3_, 333.2424).

Triregelin L (**12**): white, amorphous powder; ${\left[\alpha \right]}_{D}^{21}$ + 50.86 (*c* 0.50, MeOH); IR (KBr) *ν*_max_: 3475, 3072, 2961, 2924, 2867, 1710, 1655, 1607, 1510, 1445, 1415, 1369, 1261, 1089, 1028, 882, 801, 701, 665 cm^−1^; UV (MeOH) *λ*_max_ (log *ε*) 203 (3.15) nm; CD (*c* 1.66 × 10^−1^ mol/L, MeOH) *λ*_max_ (Δ*ε*) 203 (−24.65), 295 (+6.40); ^1^H NMR (CDCl_3_, 600 MHz) and ^13^C NMR (CDCl_3_, 150 MHz) data, see Table 1 and Table 2; HRESIMS *m*/*z* 303.2322 [M + H]^+^ (calcd for C_20_H_31_O_2_, 303.2319).

### 3.5. Calculation Methods of Electronic Circular Dichroism (ECD) Spectra

The Gaussian 09 software package [32] was used to conduct all of the ECD calculations. The molecule geometries of molecules were firstly optimized at the level of B3LYP/6-31G (d, p) and the output geometries were subsequently employed to perform ECD calculations using time-dependent density functional theory (TDDFT) with the method of B3LYP/DGDZVP [20,21] since this method usually offers desirable outcomes [33]. The model of polarizable continuum was utilized to simulate the solvation effect in the calculations of circular dichroism. The experimental condition was simulated by using methanol as the solvent. The absolute configurations of all compounds were defined by comparing the calculated ECD curves with the experimental spectra.

### 3.6. Cytotoxicity of Diterpenes against Three Cancer Cell Lines

The A2780 (ovarian carcinoma) cell line was obtained from the KeyGEN biotech (Nanjing, China). HepG2 (hepatocellular carcinoma) and MCF-7 (human breast cancer) cell lines were purchased from the American Type Culture Collection. All of the cell lines were cultured in Dulbecco’s modified Eagle medium (DMEM) (Invitrogen) supplemented with 10% (*v*/*v*) heat-inactivated fetal bovine serum (FBS) (Invitrogen), 100 U/mL penicillin, and 100 μg/mL streptomycin (Invitrogen) in a humidified atmosphere of 5% CO_2_/95% air at 37 °C. Briefly, cells were seeded in 96-well plates in triplicate at a density of 2 × 10^3^ cells/well (100 μL) and cultured at 37 °C in a 5% CO_2_ humidified atmosphere for 24 h. Then, the cells were treated with fresh culture medium containing various concentrations of tested compounds and incubated at 37 °C under a humidified atmosphere of 5% CO_2_/95% air for another 72 h. After that, the supernatant in each well was discarded and the cells were washed by phosphate-buffered saline (PBS) to avoid the possible effect of culture medium and tested compounds on the following MTT (3-(4,5-dimethylthiazol-2-yl)-2,5-diphenyltetrazolium bromide) assay. Subsequently, cells were incubated for 4 h at 37 °C in culture medium containing a final concentration of 0.5 mg/mL MTT (100 μL). The formed formazan crystals were dissolved in DMSO (100 μL) after removing the supernatant in each well. A microplate reader (Infinite 200 PRO, Tecan, Männedorf, Switzerland) was employed to determine the absorbance of each well at 570 nm. GraphPad Prism 6 software (Prism 6.0, GraphPad Software, Inc., La Jolla, CA, USA) was used to calculate the IC_50_ values (concentration that suppresses 50% of cell growth) of all tested compounds. All assays were performed in triplicate in three independent experiments. Data was expressed as mean ± SD (*n* = 3).

## 4. Conclusions

To sum up, 23 diterpenoids were isolated from the Chinese herbal medicine *T. regelii*, including eleven new abietane, and one new kaurane, diterpenes. Importantly, triregelin A (**1**) represents the first abietane diterpene bearing an 18→1 lactone ring. Triregelin I (**9**) exhibited significant cytotoxic effects on A2780 and HepG2 cancer cells with IC_50_ values of 5.88 µM and 11.74 µM, respectively, and was found inactive against MCF-7 cancer cells. Triregelin K (**11**) displayed a weak cytotoxic effect on MCF-7 cell with an IC_50_ value of 26.70 µM.

## Figures and Tables

**Table 1:** ^1^H NMR (600 MHz) spectroscopic data for compounds **1**−**5** and **12**.

Position	*δ*_H_ (*J* in Hz)					
	1 *^a^*	2 *^a^*	3 *^a^*	4 *^a^*	5 *^a^*	12 *^a^*
1	5.86, d (6.0)	1.45, m *^c^*	1.89, ddd (14.4, 10.2, 5.4)	1.90, m	1.65, m	0.79, td (13.2, 3.6)
	-	2.77, m *^c^*	2.80, ddd (14.4, 9.0, 6.0) *^c^*	2.75, m *^c^*	2.77, m *^c^*	1.79, m *^c^*
2	1.96, d (10.8)	2.46, m	2.46, m *^c^*	2.45, m *^c^*	2.52, ddd (15.6, 6.6, 3.6)	1.43, dt (13.8, 3.6)
	2.36, m *^c^*	2.55, m	2.73, ddd (16.2, 10.2, 6.0) *^c^*	2.75, m *^c^*	3.03, ddd (15.6, 7.2, 3.6) *^c^*	1.55, m *^c^*
3	-	-	-	-	-	0.95, dd (13.8, 4.2)
	-	-	-	-	-	1.79, m *^c^*
5	-	2.22, m *^c^*	2.46, d (13.8) *^c^*	2.47, d (13.8) ^c^	1.78, dd (12.6, 1.8)	1.00, m *^c^*
6	2.16, m	1.50, m *^c^*	1.65, td (13.8, 4.2)	1.41, td (13.8, 3.0)	1.59, m	1.36, qd (12.6, 3.6)
	2.76, dd (13.2, 6.0)	2.24, m *^c^*	1.96, br d (13.8)	2.02, dt (13.8, 1.8)	1.96, br d, d (13.2, 7.2)	1.72, m *^c^*
7	2.37, m *^c^*	2.39, ddd (18.6, 11.4, 7.2)	4.81, br s	4.39, dd (3.0, 1.8)	2.34, ddd (18.6, 12.0, 7.2)	1.62, dd (13.8, 4.8)
	2.90, dd (19.8, 6.0)	2.80, m	-	-	2.81, m *^c^*	1.72, m *^c^*
9	-	-	-	-	-	1.58, m *^c^*
11	-	-	-	-	-	2.24, d (17.0)
	-	-	-	-	-	2.53, dd (17.0, 9.6)
12	6.41, d (1.2)	6.47, s	6.44, s	6.42, d (1.2)	6.37, d (1.2)	
13	-	-	-	-	-	3.21, d (4.8)
14	-	-	-	-	-	1.51, dd (12.6, 4.8)
	-	-	-	-	-	2.40, d (12.6)
15	3.00, sept d (6.6, 1.2)	3.12, m	3.02, sept (7.0)	3.04, sept d (7.2, 1.2)	3.00, d (7.2) *^c^*	2.36, s
16	1.12, d (6.6)	1.17, d (7.2)	1.14, d (7.0)	1.12, d (7.2)	1.10, d (7.2)	-
17	1.11, d (6.6)	3.67, d (7.2)	1.13, d (7.0)	1.13, d (7.2)	1.11, d (7.2)	4.87, s
	-	-	-	-	-	4.99, s
18	-	-	1.37, s	1.36, s	1.22, s	0.98, s
19	1.85, s	2.11, s	3.47, t (10.8)	3.44, d (12.0)	4.56, d (12.0)	3.68, dd (10.1, 4.0)
	-	-	4.02, dd (10.8, 2.4)	4.05, d (12.0)	4.08, d (12.0)	3.44, dd (10.1, 4.0)
20	1.54, s	1.18, s	1.24, s	1.22, s	1.44, s	0.85, s
OH-7	-	-	2.77, s *^c^*	-	-	-
OH-19	-	-	3.15, dd (10.8, 2.4)	3.26, br s	-	1.09, br s
OMe-7	-	-	-	3.50, s	-	-
OAc-19	-	-	-	-	2.03, s	-

*^a^* Measured in CDCl_3_; *^c^* Overlapping signal was assigned from ^1^H–^1^H COSY, HSQC and HMBC experiments. The signals of br, s, d, t, q, sept and m represent broad, singlet, doublet, triplet, quartet, septet and multiplet splitting patterns of protons, respectively.

**Table 2:** ^13^C NMR (150 MHz) spectroscopic data for compounds **1**–**12**.

Position	*δ*_C_, Type											
	1 *^a^*	2 *^a^*	3 *^a^*	4 *^a^*	5 *^a^*	6 *^a^*	7 *^a^*	8 *^a^*	9 *^a^*	10 *^a^*	11 *^b^*	12 *^a^*
1	78.4, CH	31.8, CH_2_	34.1, CH_2_	34.1, CH_2_	34.7, CH_2_	38.2, CH_2_	35.1, CH_2_	35.1, CH_2_	34.5, CH_2_	36.2, CH_2_	32.8, CH_2_	39.7, CH_2_
2	39.1, CH_2_	24.6, CH_2_	34.2, CH_2_	34.2, CH_2_	34.9, CH_2_	27.4, CH_2_	35.3, CH_2_	34.8, CH_2_	28.3, CH_2_	28.1, CH_2_	27.0, CH_2_	17.8, CH_2_
3	74.3, C	147.9, C	220.4, C	220.8, C	212.4, C	46.1, CH	214.8, C	219.0, C	79.7, CH	79.9, CH	74.8, CH	35.5, CH_2_
4	130.2, C	124.5, C	49.9, C	49.7, C	51.3, C	150.8, C	53.0, C	50.3, C	42.8, C	42.3, C	38.3, C	38.6, C
5	132.6, C	47.3, CH	45.2, CH	44.9, CH	52.9, CH	47.9, CH	51.5, CH	49.4, CH	49.6, CH	48.8, CH	44.0, CH	56.2, CH
6	22.2, CH_2_	18.7, CH_2_	26.1, CH_2_	22.5, CH_2_	18.5, CH_2_	20.8, CH_2_	124.0, CH	35.6, CH_2_	35.5, CH_2_	35.7, CH_2_	18.9, CH_2_	20.2, CH_2_
7	27.0, CH_2_	25.2, CH_2_	61.9, CH	69.8, CH	26.0, CH_2_	23.7, CH_2_	122.1, CH	204.3, C	205.6, C	205.2, C	25.1, CH_2_	39.4, CH_2_
8	146.0, C	142. 6, C	140.9, C	139.3, C	142.8, C	120.7, C	113.6, C	114.9, C	115.1, C	114.2, C	119.7, C	44.1, C
9	144.6, C	149.0, C	148.7, C	148.6, C	148.0, C	145.8, C	145.2, C	133.1, C	134.7, C	153.1, C	150.0, C	57.6, CH
10	44.7, C	36.6, C	37.5, C	37.3, C	37.6, C	39.7, C	37.7, C	38.3, C	39.5, C	37.5, C	37.9, C	39.2, C
11	187.3, C	187.4, C	187.8, C	187.9, C	187.5, C	117.6, CH	98.4, CH	144.5, C	144.2, C	113.6, CH	108.8, CH	35.9, CH_2_
12	131.6, CH	134.0, CH	132.4, CH	131.8, CH	132.0, CH	123.3, CH	158.3, C	123.8, CH	123.9, CH	133.6, CH	156.5, C	211.5, C
13	153.9, C	149.0, C	153.6, C	154.0, C	153.3, C	130.3, C	119.6, C	136.6, C	136.3, C	134.9, C	125.1, C	60.7, CH
14	186.8, C	188.1, C	188.7, C	186.4, C	187.4, C	150.3, C	150.3, C	155.4, C	155.7, C	160.7, C	156.9, C	39.4, CH_2_
15	26.6, CH	34.5, CH	26.4, CH	26.5, CH	26.4, CH	26.9, CH	24.3, CH	26.0, CH	26.0, CH	26.1, CH	26.1, CH	48.2, CH
16	21.3, CH_3_	15.4, CH_3_	21.3, CH_3_	21.3, CH_3_	21.3, CH_3_	22.6, CH_3_	20.9, CH_3_	22.1, CH_3_	22.1, CH_3_	22.1, CH_3_	21.6, CH_3_	148.8, C
17	21.4, CH_3_	66.6, CH_2_	21.3, CH_3_	21.4, CH_3_	21.3, CH_3_	22.8, CH_3_	20.9, CH_3_	22.2, CH_3_	22.2, CH_3_	22.3, CH_3_	21.7, CH_3_	107.8, CH_2_
18	177.3, C	173.7, C	22.3, CH_3_	22.3, CH_3_	21.8, CH_3_	64.7, CH_2_	19.7, CH_3_	22.6, CH_3_	22.4, CH_3_	22.0, CH_3_	29.1, CH_3_	26.9, CH_3_
19	11.8, CH_3_	18.5, CH_3_	65.7, CH_2_	65.8, CH_2_	65.7, CH_2_	104.5, CH_2_	65.9, CH_2_	65.5, CH_2_	63.7, CH_2_	63.7, CH_2_	22.4, CH_3_	65.4, CH_2_
20	23.7, CH_3_	19.2, CH_3_	19.7, CH_3_	20.0, CH_3_	20.2, CH_3_	22.5, CH_3_	20.3, CH_3_	18.3, CH_3_	18.3, CH_3_	24.2, CH_3_	25.2, CH_3_	16.4, CH_3_
OMe-7	-	-	-	57.9, CH_3_	-	-	-	-	-	-	-	-
OMe-12	-	-	-	-	-	-	55.7, CH_3_	-	-	-	-	-
OMe-14	-	-	-	-	-	-	-	-	-	-	60.5, CH_3_	-
OAc-19	-	-	-	-	20.9, CH_3_	-	-	-	-	-	-	-
	-	-	-	-	170.8, C	-	-	-	-	-	-	-

*^a^* Measured in CDCl_3_; *^b^* Measured in pyridine-*d*_5_.

**Table 3:** ^1^H NMR (600 MHz) spectroscopic data for compounds **6**−**11**.

Position	*δ*_H_ (*J* in Hz)					
	6 *^a^*	7 *^a^*	8 *^a^*	9 *^a^*	10 *^a^*	11 *^b^*
1	1.63, td (13.2, 4.2)	2.14, td (13.2, 5.4)	2.07, ddd (16.2, 9.6, 4.8)	1.50, td (13.8, 3.6)	1.70, td (13.8, 4.2)	1.97, dt (12.6, 3.6)
	2.32, dt (13.2, 4.2)	2.48, ddd (12.6, 6.0, 3.0)	3.31, m *^c^*	3.34, dt (13.8, 3.6)	2.36, dt (13.8, 3.0)	2.38, td (13.2, 3.6)
2	1.43, qd (13.0, 4.2)	2.61, ddd (15.6, 5.4, 3.0)	2.54, ddd (15.6, 8.4, 7.2)	1.89, m *^c^*	1.98, m	1.87, m *^c^*
	1.92, m *^c^*	2.83, ddd (15.6, 13.2, 6.0)	2.74, m *^c^*	2.02, m	2.04, m	2.10, tt (14.4, 3.6)
3	2.20, m *^c^*	-	-	3.56, dd (11.4, 3.6)	3.55, dd (11.4, 3.6)	3.68, q (3.6)
5	2.17, d (12.6) *^c^*	2.69, t (3.0)	2.64, d (15.0) *^c^*	1.91, dd (14.4, 2.4) *^c^*	1.94, dd (14.4, 3.6)	2.22, dd (12.6, 2.4)
6	1.81, qd (12.6, 6.0)	5.86, dd (10.2, 3.0)	2.63, d (16.2)	2.64, dd (16.8, 14.4)	2.67, dd (18.0, 14.4)	1.88, m *^c^*
	1.95, m *^c^*	-	2.72, m *^c^*	2.74, dd (16.8, 2.4)	2.80, dd (18.0, 3.6)	1.72, m *^c^*
7	2.61, ddd (16.4, 12.6, 7.2)	6.82, dd (10.2, 3.0)	-	-	-	2.81, ddd (16.2, 11.4, 7.8)
	2.87, dd (16.4, 6.0)	-	-	-	-	3.13, dd (16.2, 6.6)
11	6.90, d (7.8)	6.34, s	-	-	6.74, d (7.8)	7.07, s
12	7.04, d (7.8)	-	6.83, s	6.77, s	7.36, d (7.8)	-
15	3.15, sept (7.2)	3.44, sept (7.2)	3.32, sept (6.6) *^c^*	3.30, sept (6.6)	3.32, sept (6.6)	3.76, sept (7.2)
16	1.26, d (7.2)	1.33, d (7.2)	1.20, d (6.6)	1.18, d (6.6)	1.22, d (6.6)	1.72, d (7.2) *^c^*
17	1.25, d (7.2)	1.33, d (7.2)	2.21, d (6.6)	1.20, d (6.6)	1.20, d (6.6)	1.68, d (7.2)
18	3.70, dd (10.8, 6.0)	1.27, s	1.35, s	1.30, s	1.29, s	1.27, s
	3.95, dd (10.8, 6.0)	-	-	-	-	-
19	4.77, s	3.84, d (12.0, 4.8)	3.54, d (11.4)	3.42, dd (11.4, 9.0)	3.50, dd (11.4, 7.8)	0.96, s
	4.86, s	4.14, d (12.0)	4.06, d (11.4)	4.35, d (11.4)	4.36, d (11.4)	-
20	0.99, s	1.21, s	1.42, s	1.35, s	1.19, s	1.25, s
OH-3	-	-	-	-	2.56, s	5.69, d (3.6)
OH-11	-	-	4.62, s	4.47, s	-	-
OH-12	-	-	-	-	-	10.80, s
OH-14	-	4.90, s	12.79, s	12.96, s	13.07, s	-
OH-19	-	1.77, br s	2.96, s	2.80, br d (9.0)	2.83, br d (7.8)	-
OMe-12	-	3.80, s	-	-	-	-
OMe-14	-	-	-	-	-	3.72, s

*^a^* Measured in CDCl_3_; *^b^* Measured in pyridine-*d_5_*; *^c^* Overlapping signal was assigned from ^1^H–^1^H COSY, HSQC, and HMBC experiments. The signals of br, s, d, t, q, sept and m represent broad, singlet, doublet, triplet, quartet, septet and multiplet splitting patterns of protons, respectively.

**Table 4:** Cytotoxic effects of diterpenes on three cancer cell lines of A2780, HepG2, and MCF-7.

Compounds *	IC_50_ (µM) against A2780	IC_50_ (µM) against HepG2	IC_50_ (µM) against MCF-7
**9**	5.88 ± 2.22	11.74 ± 1.92	46.40 ± 3.54
**11**	>100	>100	26.70 ± 5.57
**14**	65.80 ± 21.53	35.45 ± 8.23	64.80 ± 24.90
taxol	0.006 ± 0.001	0.003 ± 0.0002	0.005 ± 0.001

* Seventeen compounds (**2**, **7**–**11**, **13**–**23**) were evaluated for cytotoxic effects against three cancer cell lines; IC_50_ values for other tested compounds were larger than 100 µM on three cancer cells.

## Supplementary Materials

Supplementary materials can be found at www.mdpi.com/1422-0067/18/1/147/s1.

## Abbreviations

HRESIMS	High resolution electrospray ionization mass spectrometry
CD	Circular dichroism
UV	Ultraviolet visible
IR	Infrared
NMR	Nuclear magnetic resonance
DEPT	Distortionless enhancement by polarization transfer
HSQC	Heteronuclear single quantum coherence
HMBC	Heteronuclear multiple bond correlation
^1^H–^1^H COSY	Proton–proton correlation spectroscopy
NOESY	Nuclear Overhauser effect spectroscopy
