# Supplementary material for: New Abietane and Kaurane Type Diterpenoids from the Stems of Tripterygium regelii
Source: Int J Mol Sci. 2017 Jan 13;18(1):147. doi: 10.3390/ijms18010147 (PMC5297780; doi:10.3390/ijms18010147)
Supplement: Supplementary file 1 [file ijms-18-00147-s001.pdf]

# Supplementary Material: New Abietane and Kaurane Type Diterpenoids from the Stems of *Tripterygium regelii*

**Dongsheng Fan, Shuangyan Zhou, Zhiyuan Zheng, Guo-Yuan Zhu, Xiaojun Yao, Ming-Rong Yang, Zhi-Hong Jiang and Li-Ping Bai**

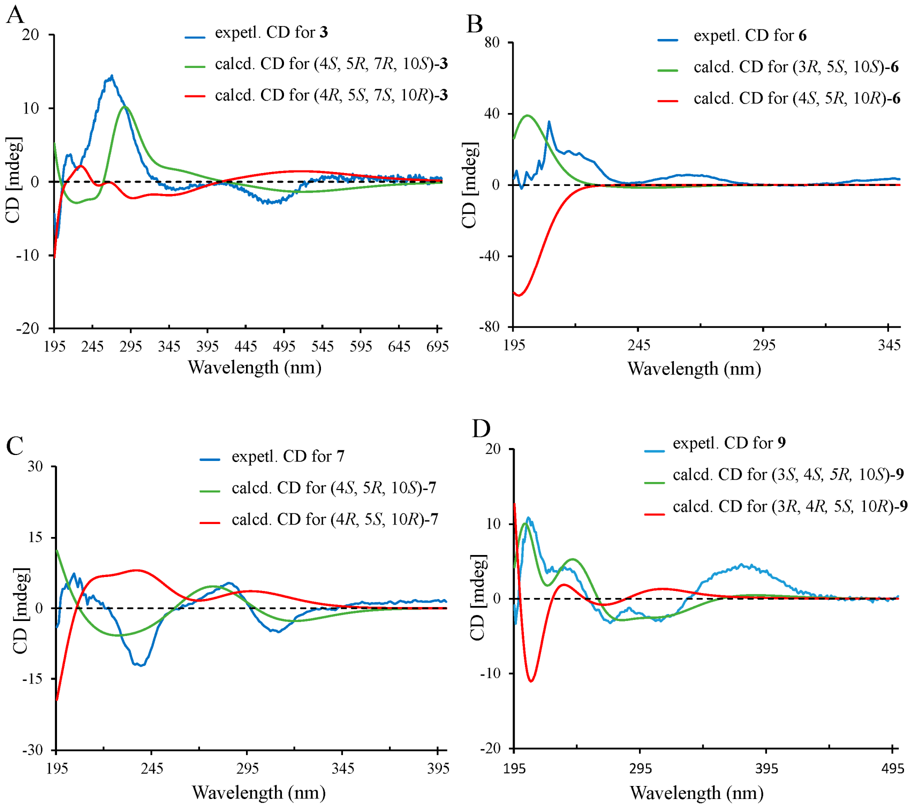


**Figure S1.** Experimental and calculated CD spectra of compounds **3** (**A**), **6** (**B**), **7** (**C**) and **9** (**D**).

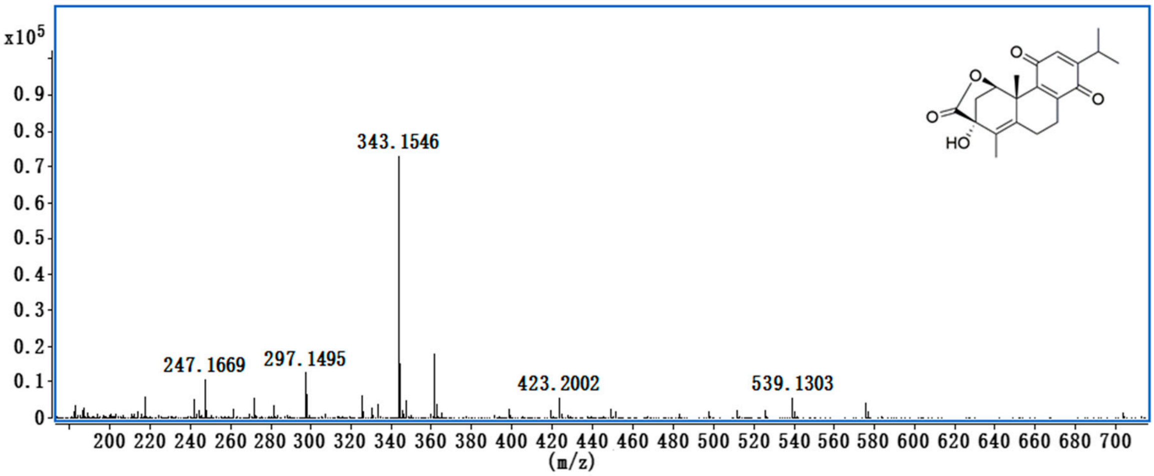


**Figure S2.** HRESIMS spectrum of compound **1**.

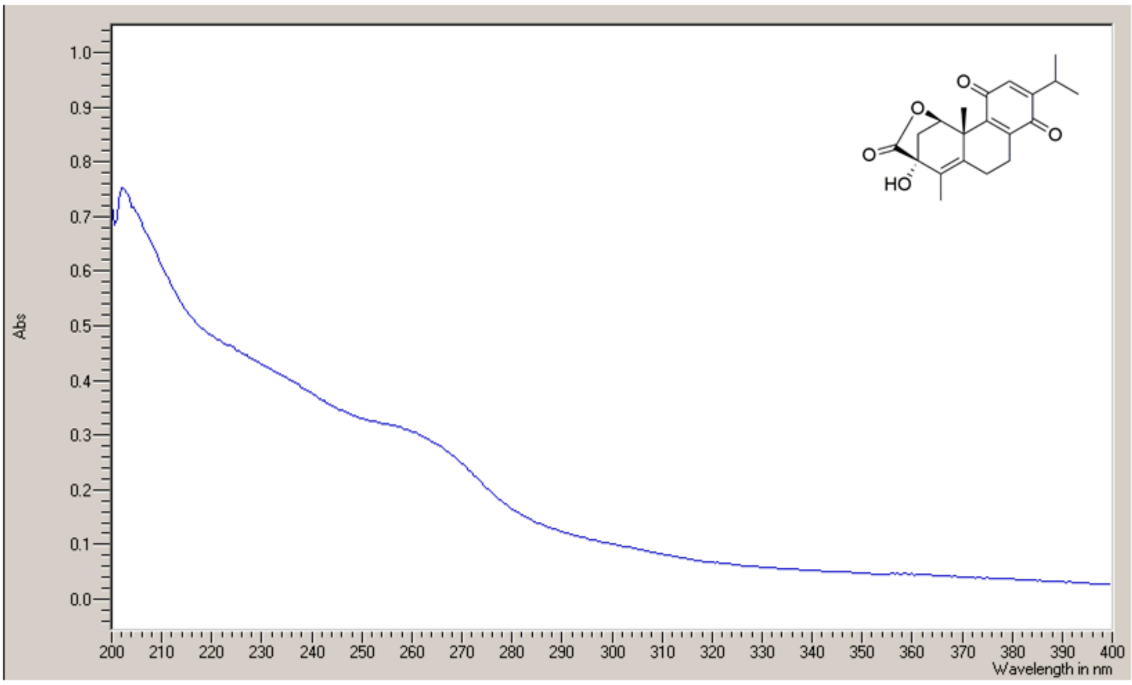


**Figure S3.** UV spectrum of compound **1** in MeOH.

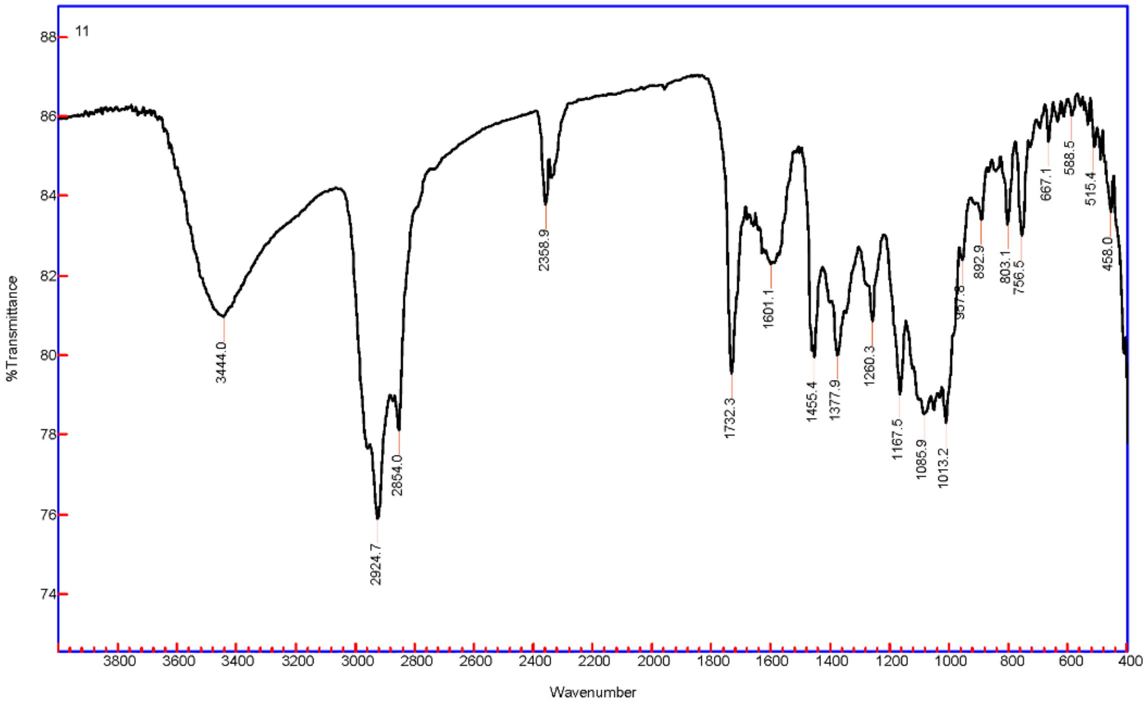


**Figure S4.** IR spectrum of compound **1**.

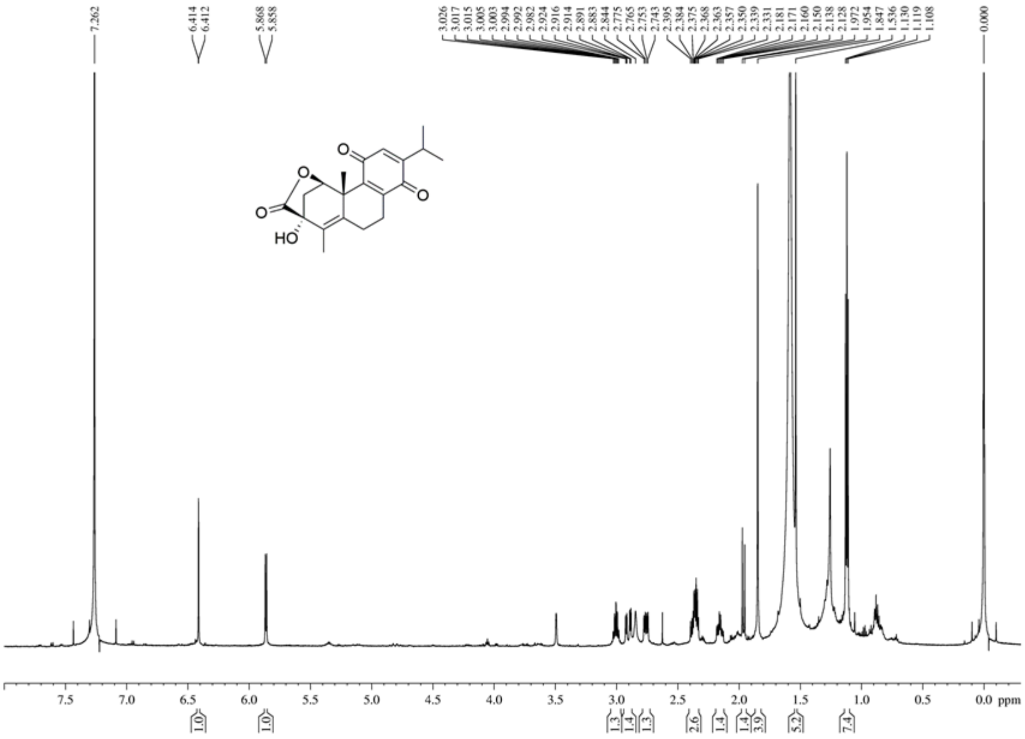


**Figure S5.** $^{1}H$ NMR spectrum of compound **1** in $CDCl_3$.

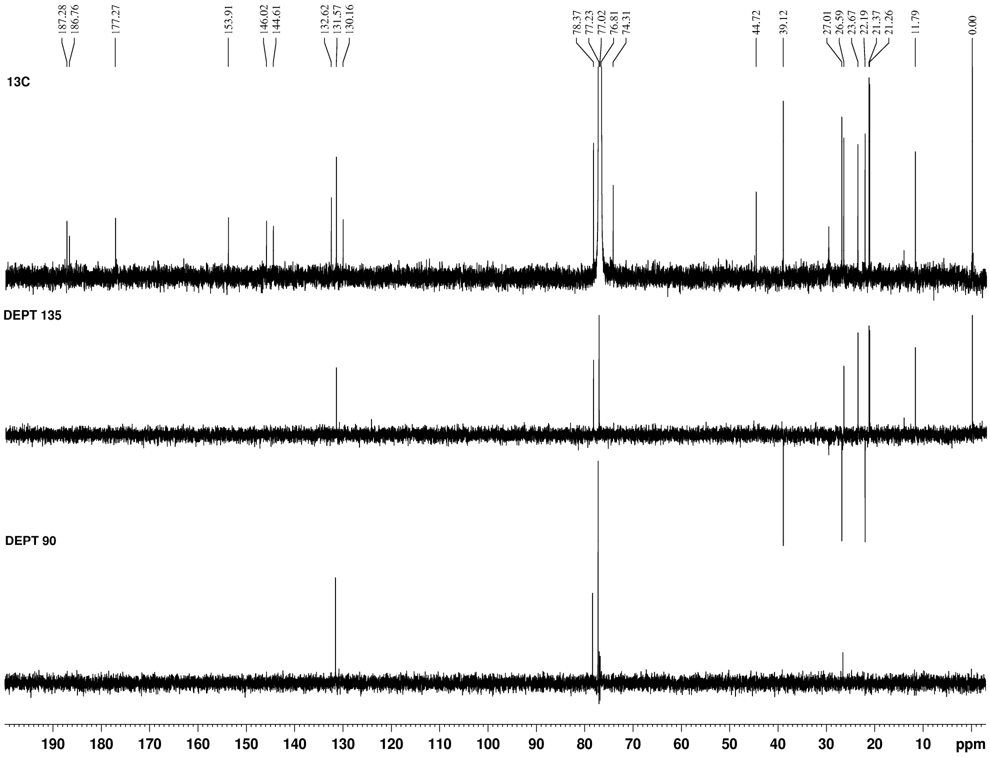


**Figure S6.** $^{13}C$ and DEPT NMR spectra of compound **1** in $CDCl_3$.

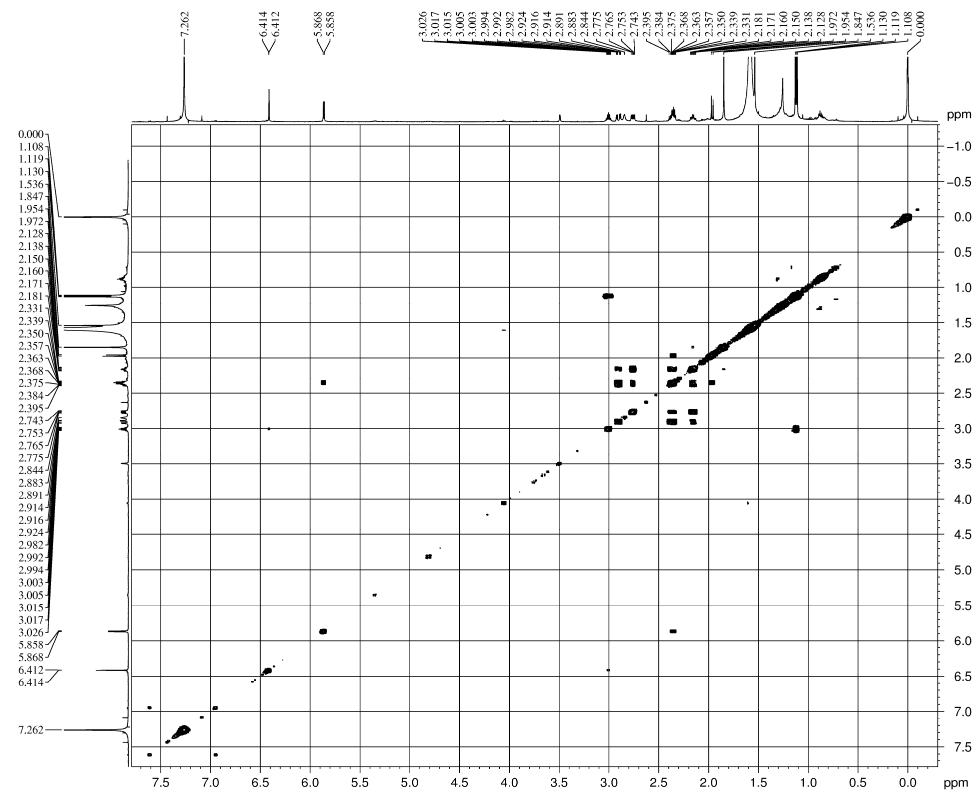


**Figure S7.** $^{1}H$–$^{1}H$ COSY spectrum of compound **1** in $CDCl_3$.

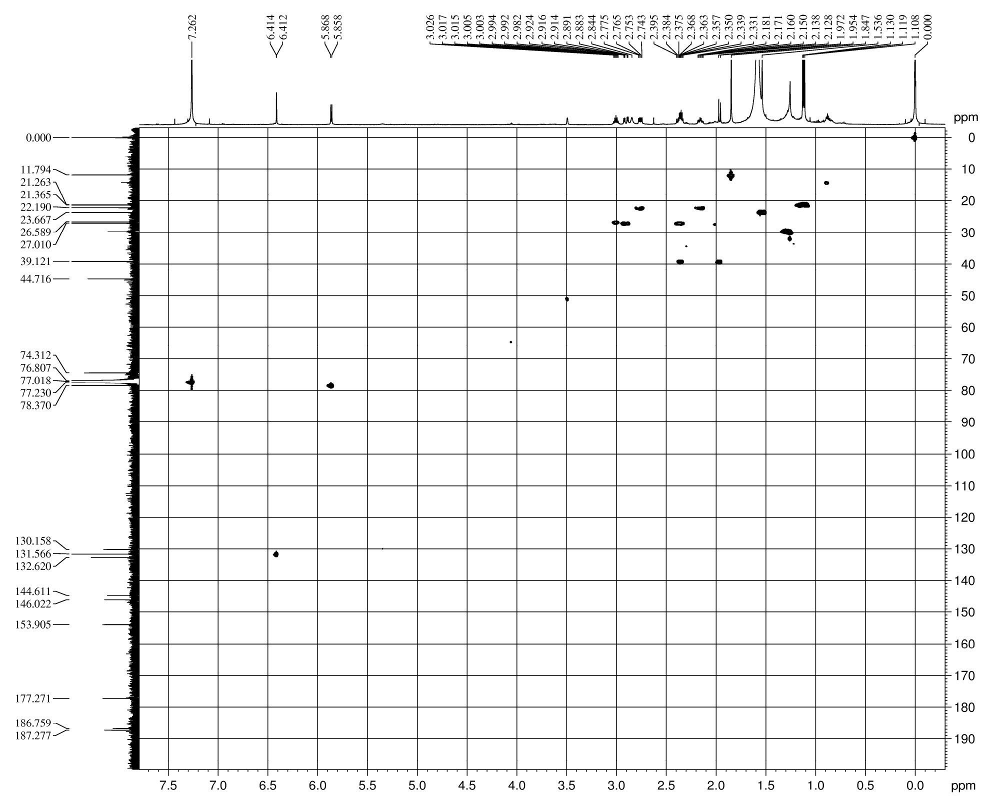


**Figure S8.** HSQC spectrum of compound **1** in $CDCl_3$.

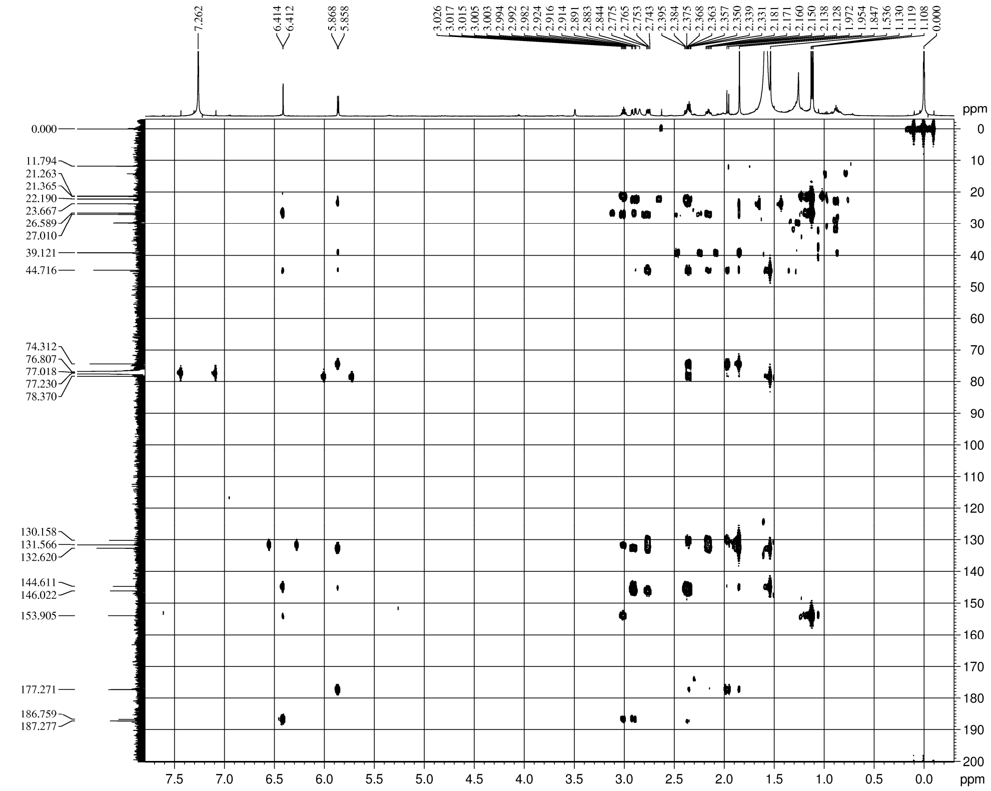


**Figure S9.** HMBC spectrum of compound **1** in $CDCl_3$.

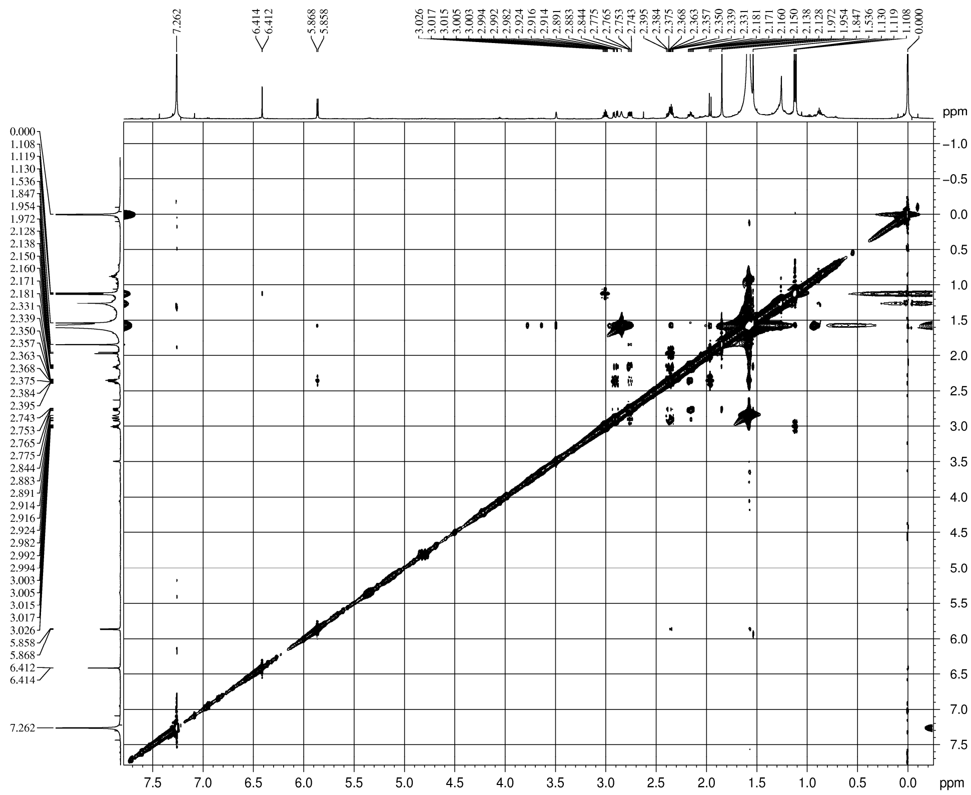


**Figure S10.** NOESY spectrum of compound **1** in $CDCl_3$.

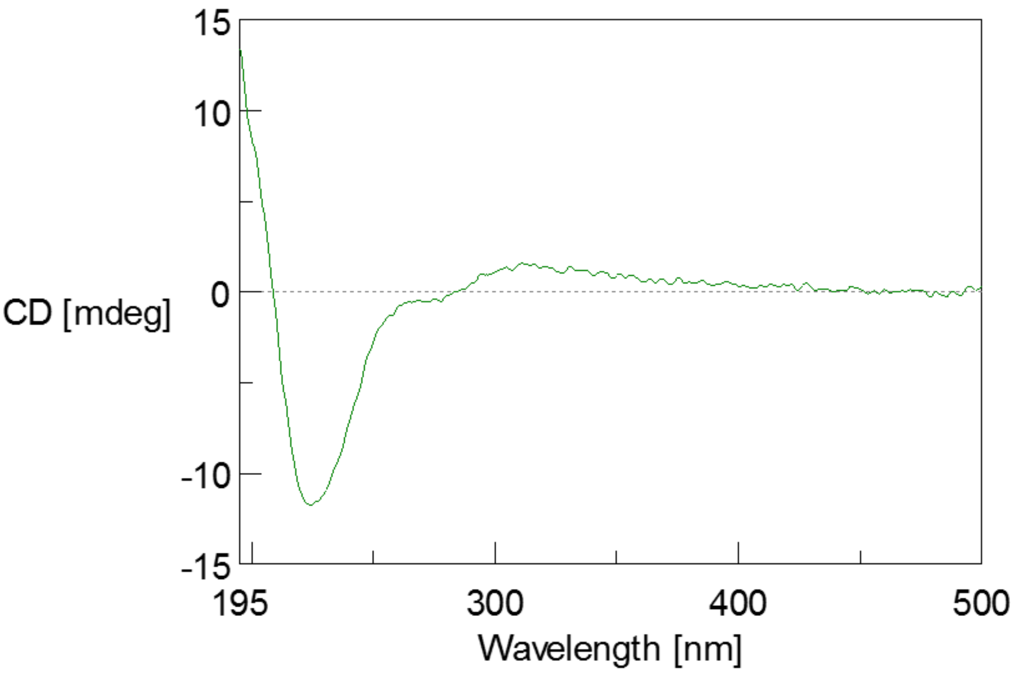


**Figure S11.** CD spectrum of compound **1** in MeOH.

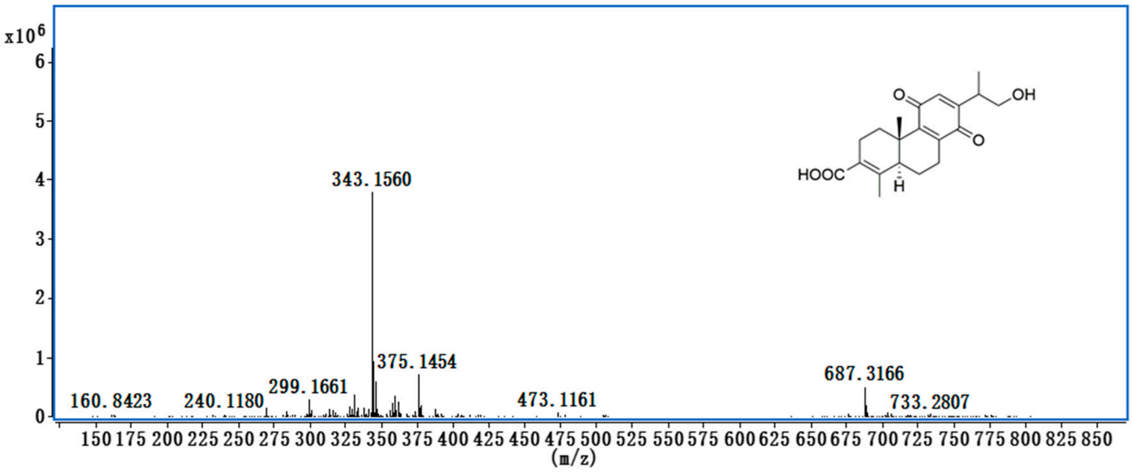


**Figure S12.** HRESIMS spectrum of compound **2**.

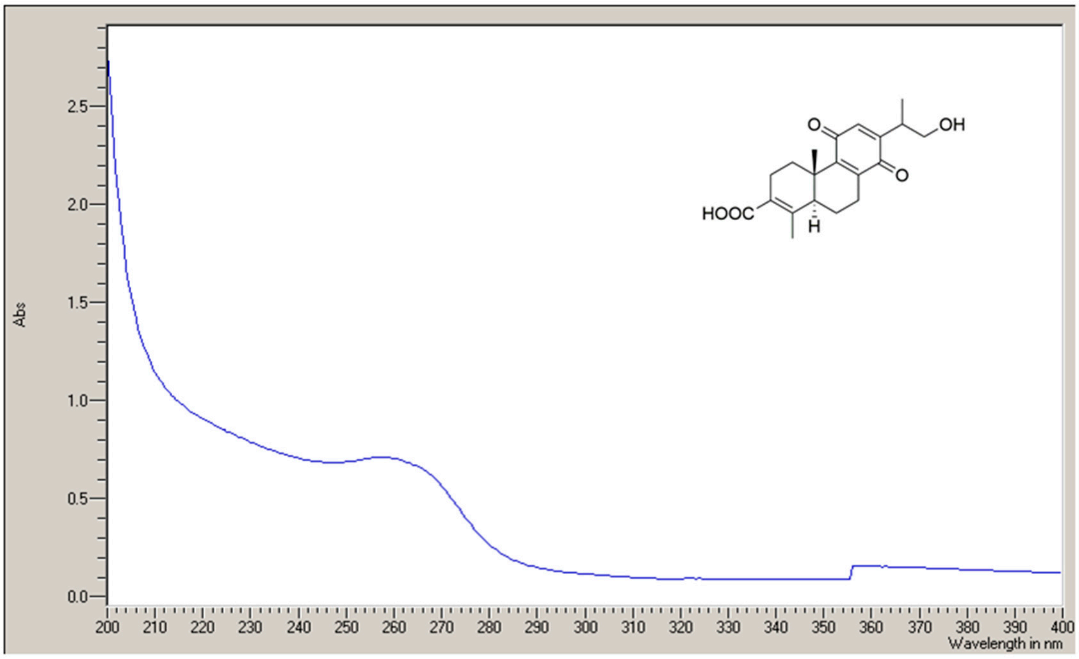


**Figure S13.** UV spectrum of compound **2** in MeOH.

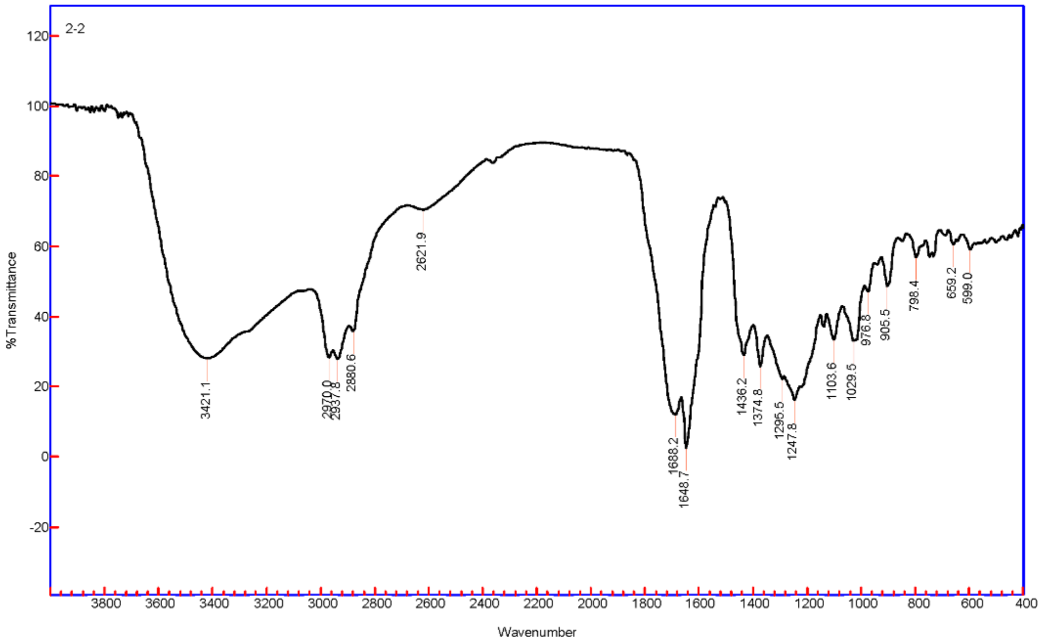


**Figure S14.** IR spectrum of compound **2**.

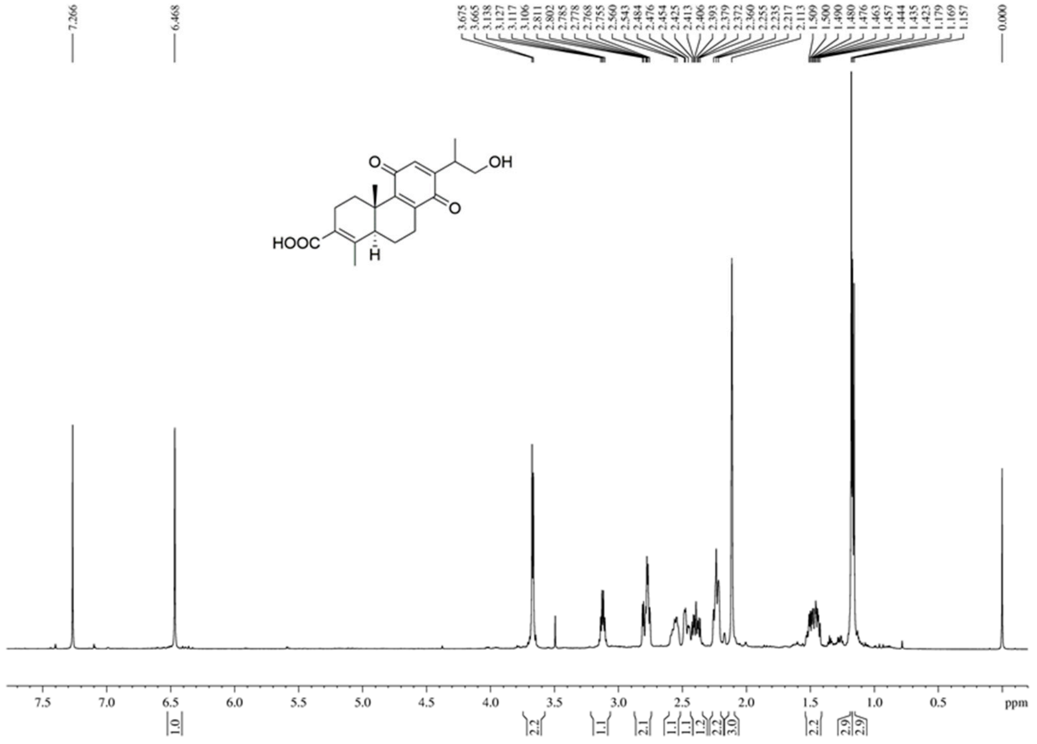

**Figure S15.** $^{1}$H NMR spectrum of compound **2** in $CDCl_3$.

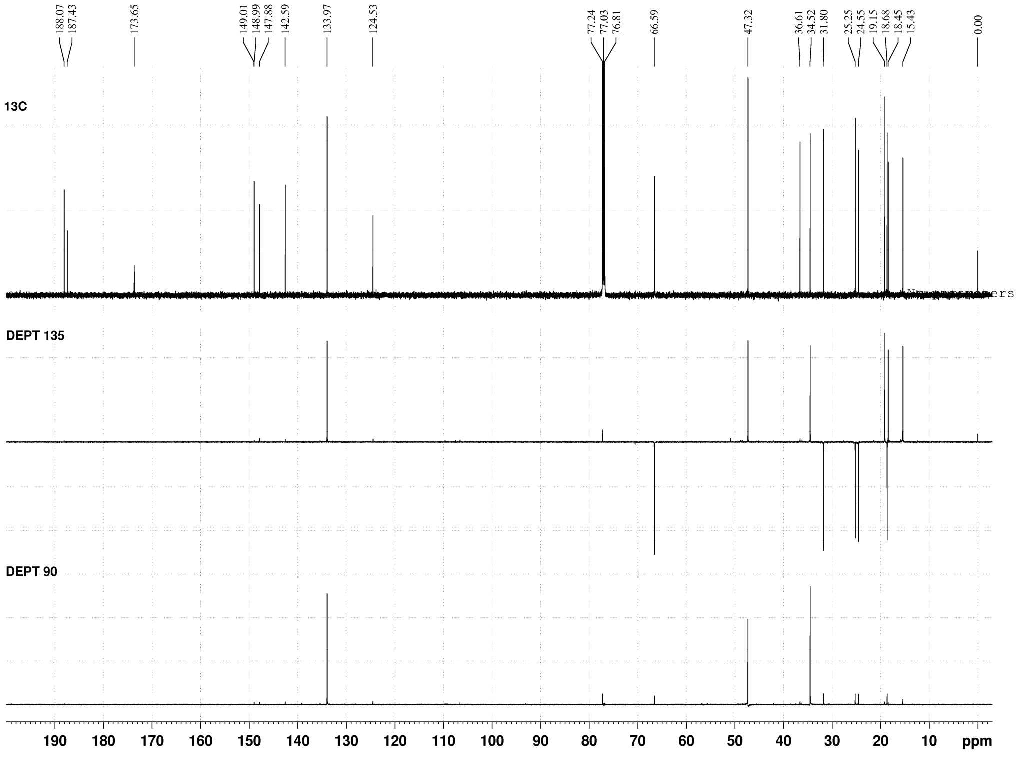


**Figure S16.** $^{13}$C and DEPT NMR spectra of compound **2** in $CDCl_3$.

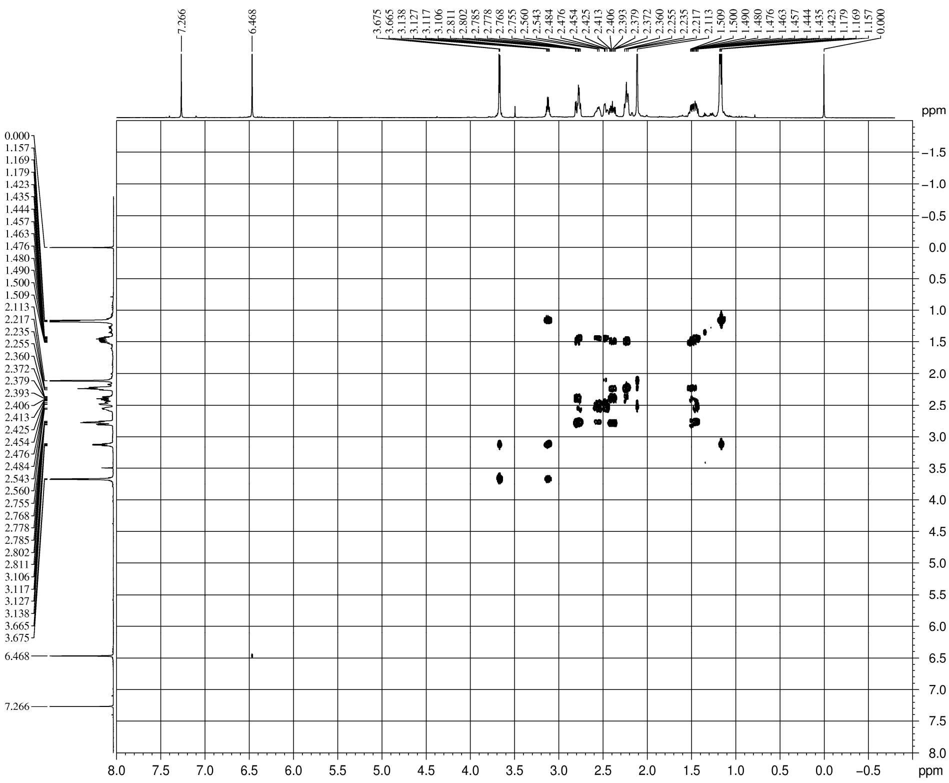


**Figure S17.** $^1H$–$^1H$ COSY spectrum of compound **2** in $CDCl_3$.

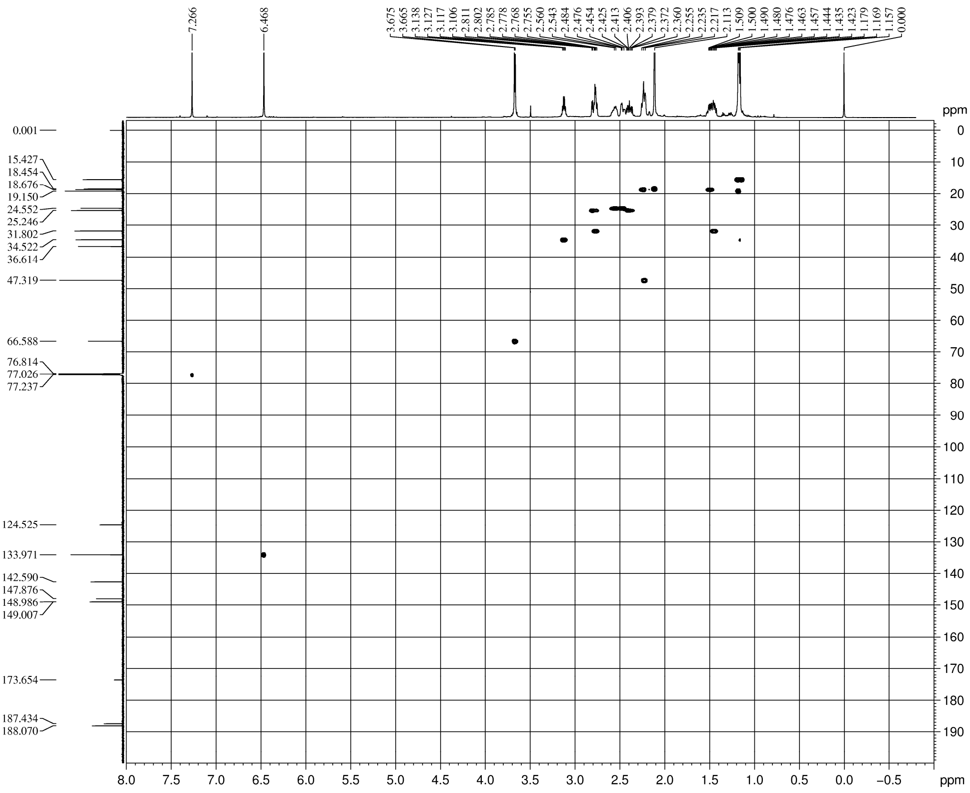


**Figure S18.** HSQC spectrum of compound **2** in $CDCl_3$.

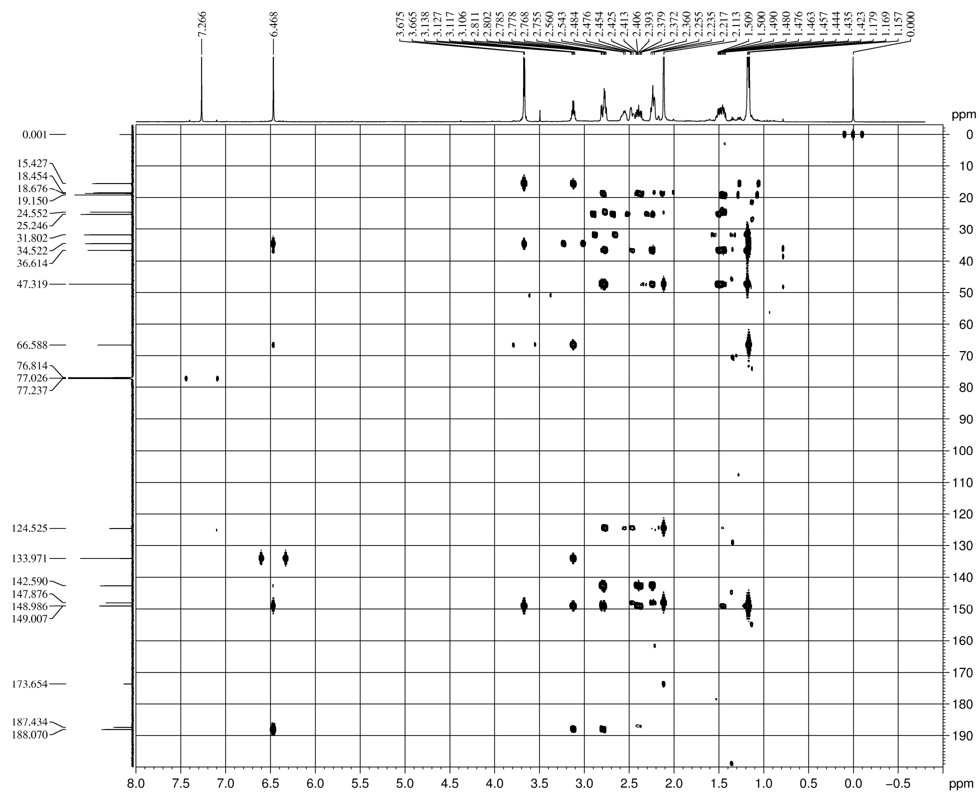

**Figure S19.** HMBC spectrum of compound **2** in $CDCl_3$.

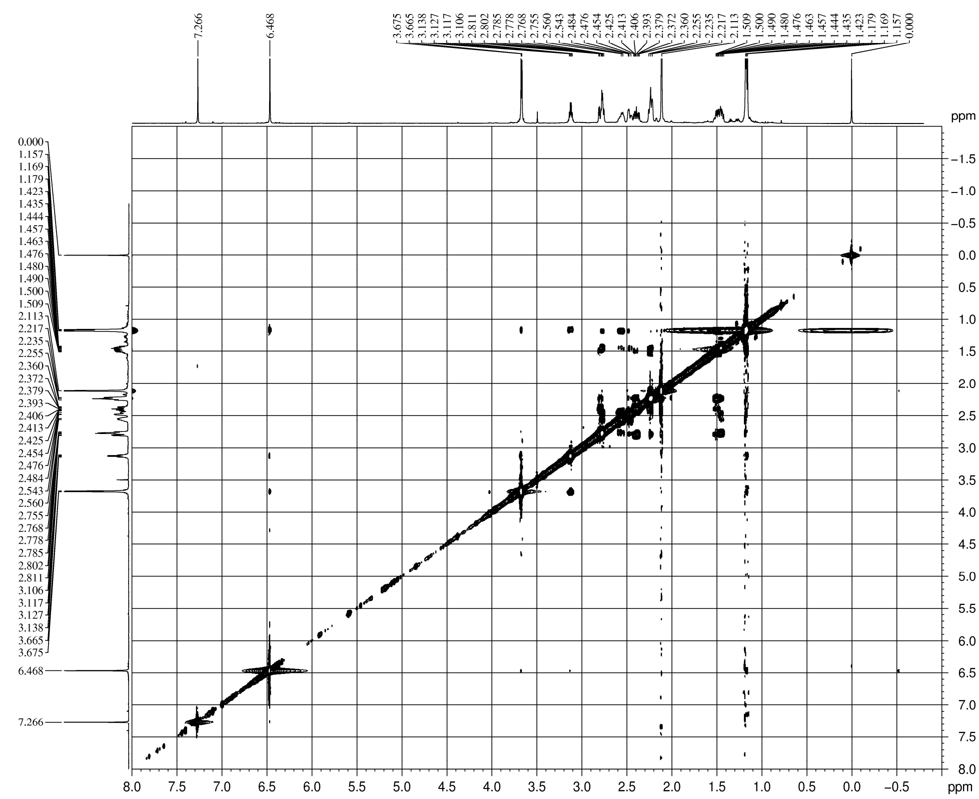

**Figure S20.** NOESY spectrum of compound **2** in $CDCl_3$.

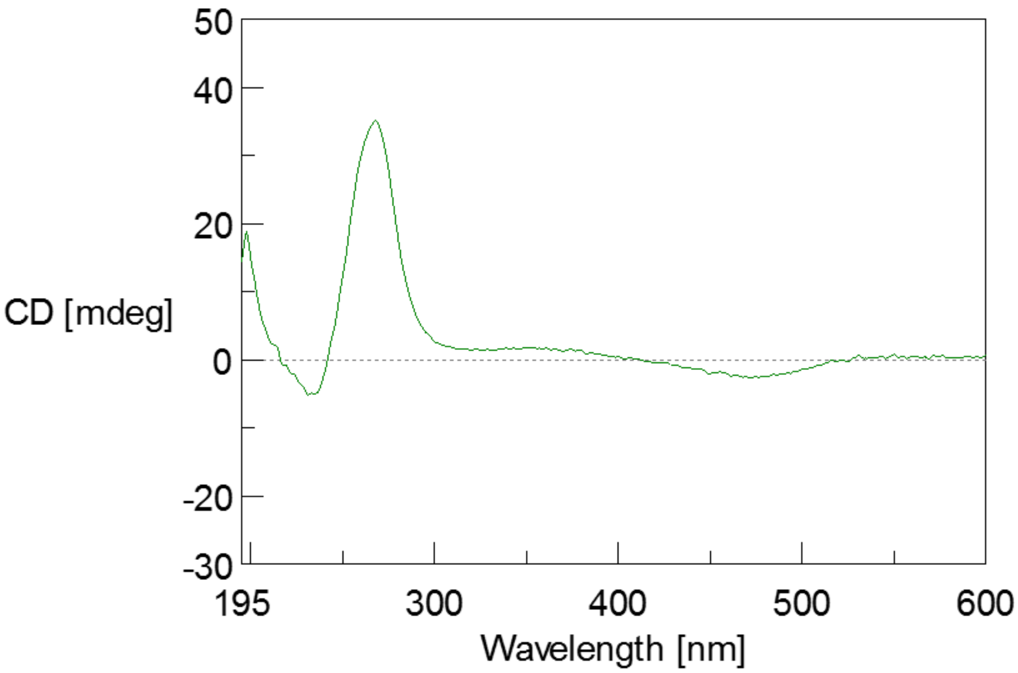


**Figure S21.** CD spectrum of compound **2** in MeOH.

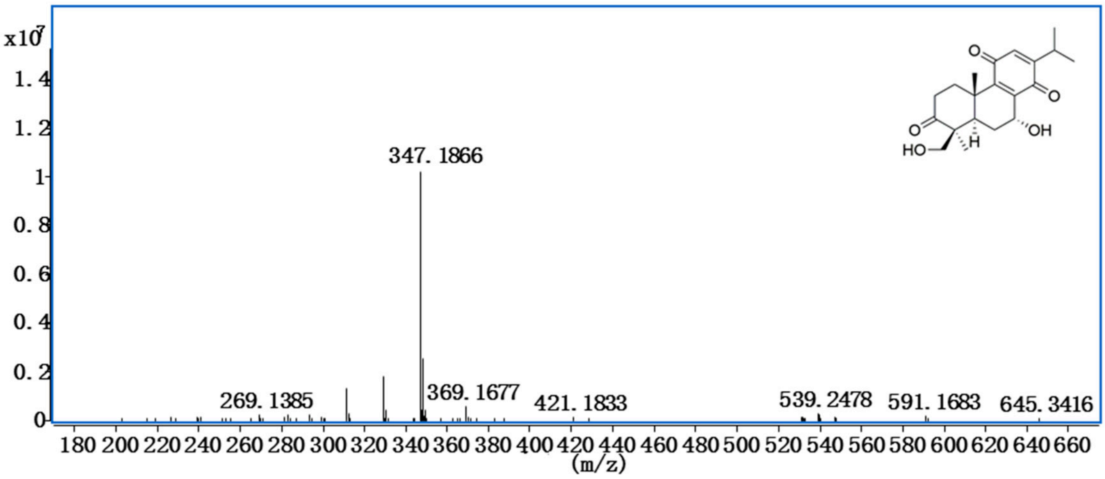


**Figure S22.** HRESIMS spectrum of compound **3**.

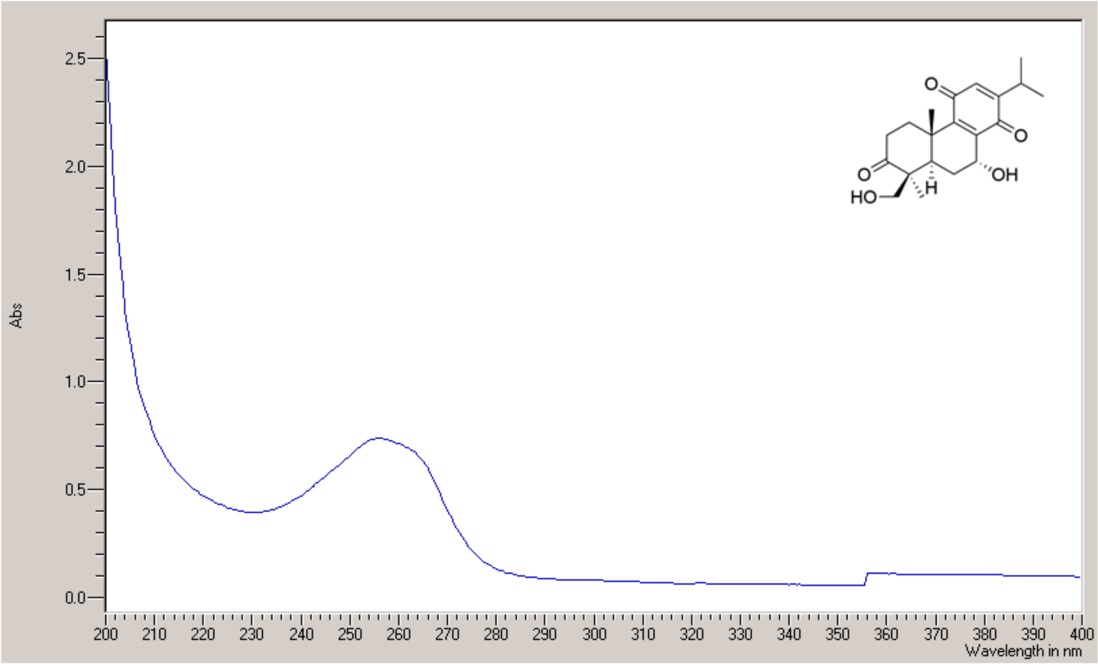


**Figure S23.** UV spectrum of compound **3** in MeOH.

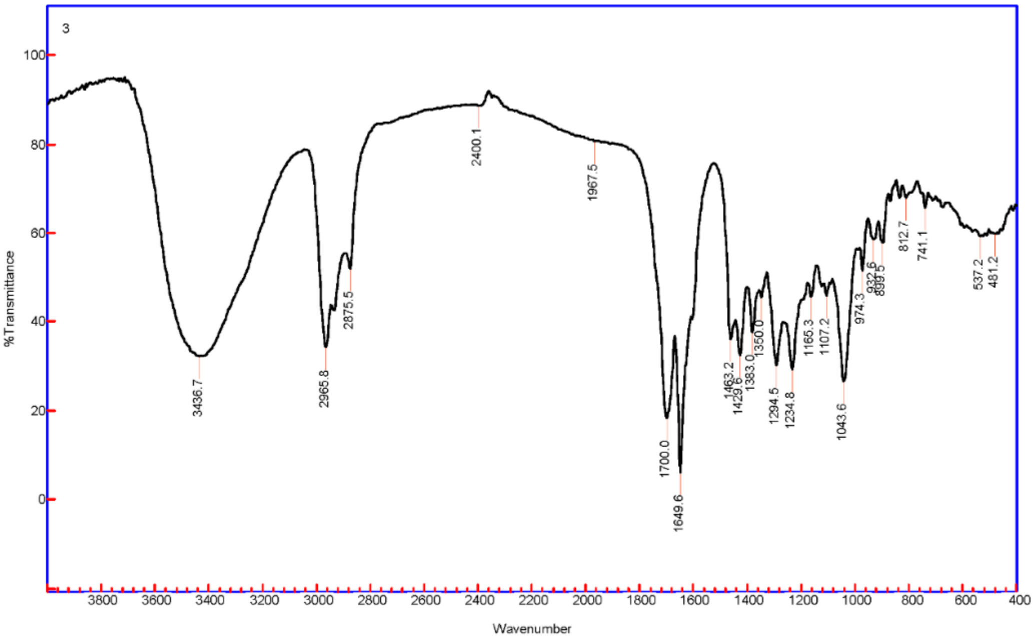


**Figure S24.** IR spectrum of compound **3**.

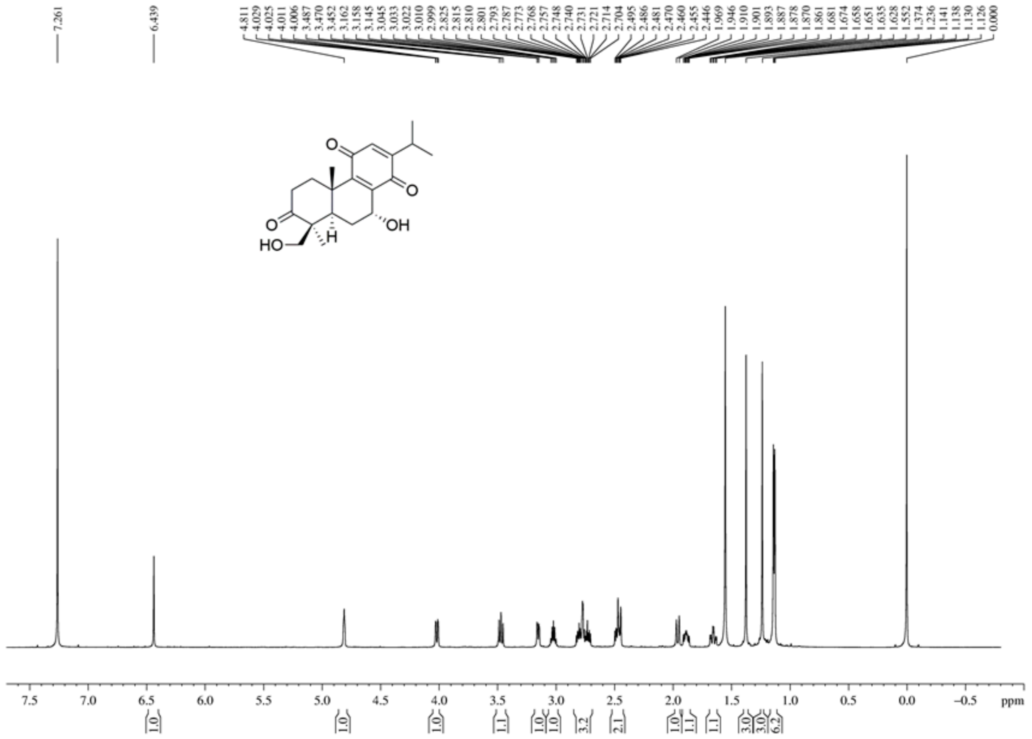


**Figure S25.** $^{1}H$ NMR spectrum of compound **3** in $CDCl_3$.

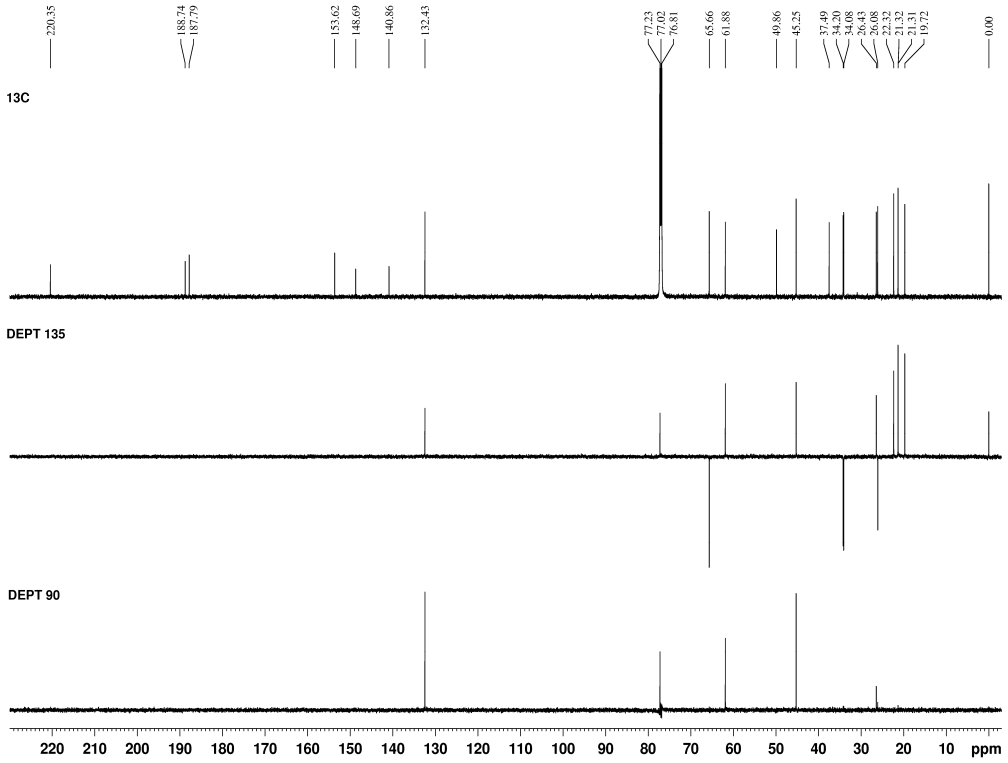


**Figure S26.** $^{13}C$ and DEPT NMR spectra of compound **3** in $CDCl_3$.

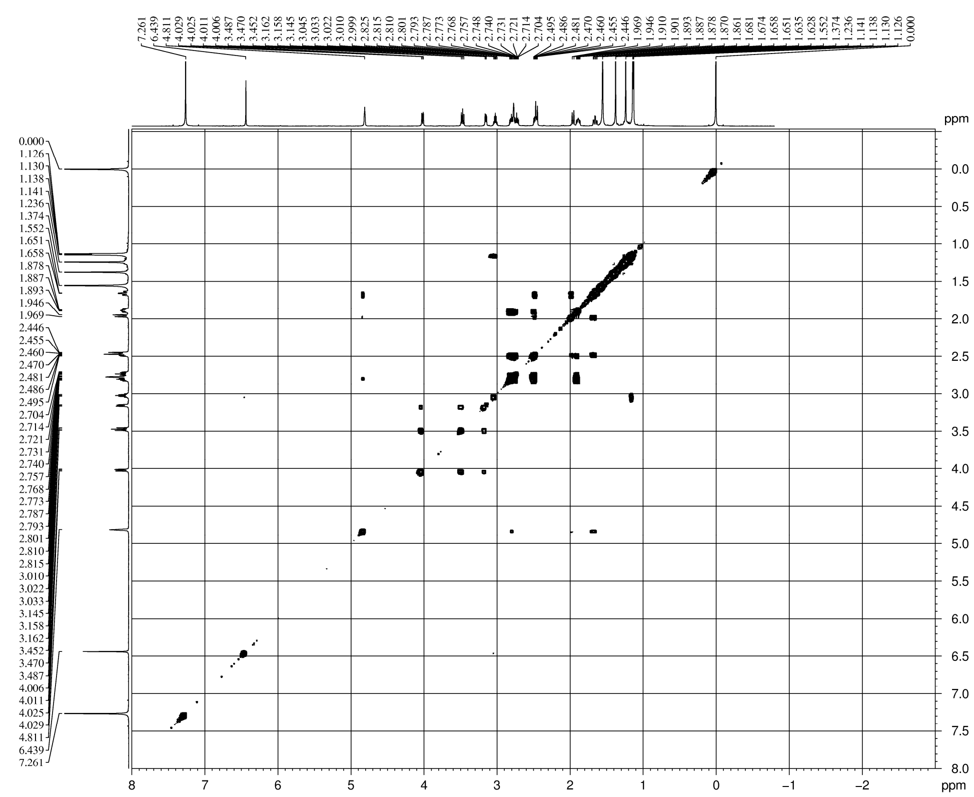


**Figure S27.** $^{1}H$–$^{1}H$ COSY spectrum of compound **3** in $CDCl_3$.

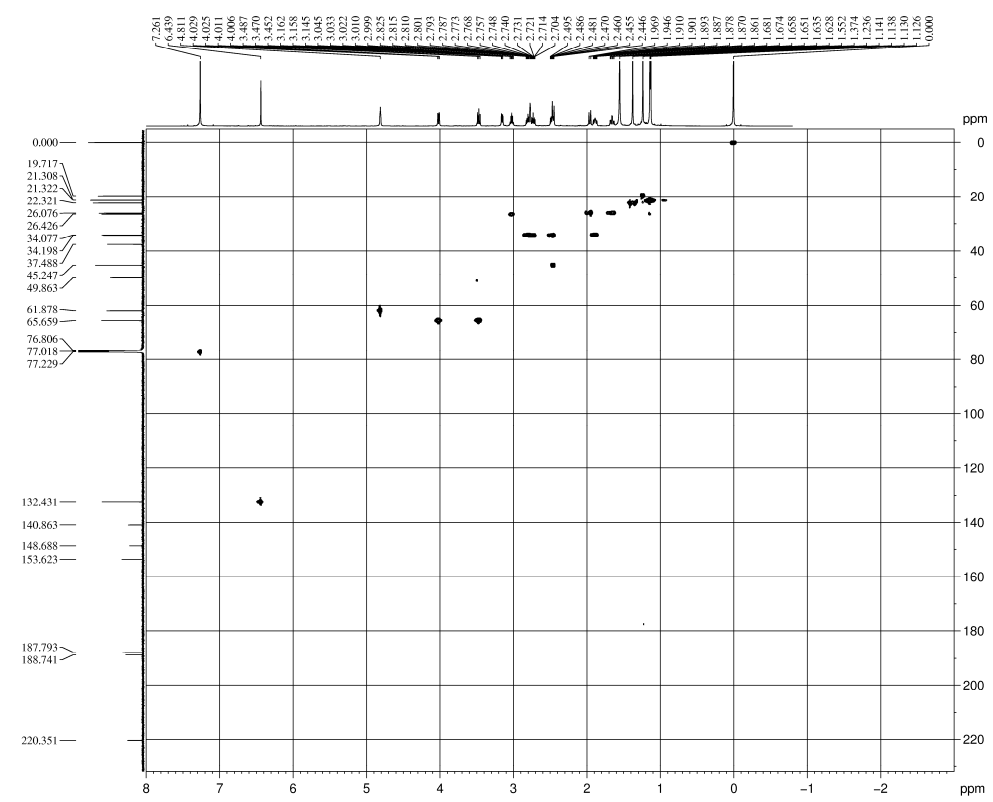


**Figure S28.** HSQC spectrum of compound **3** in $CDCl_3$.

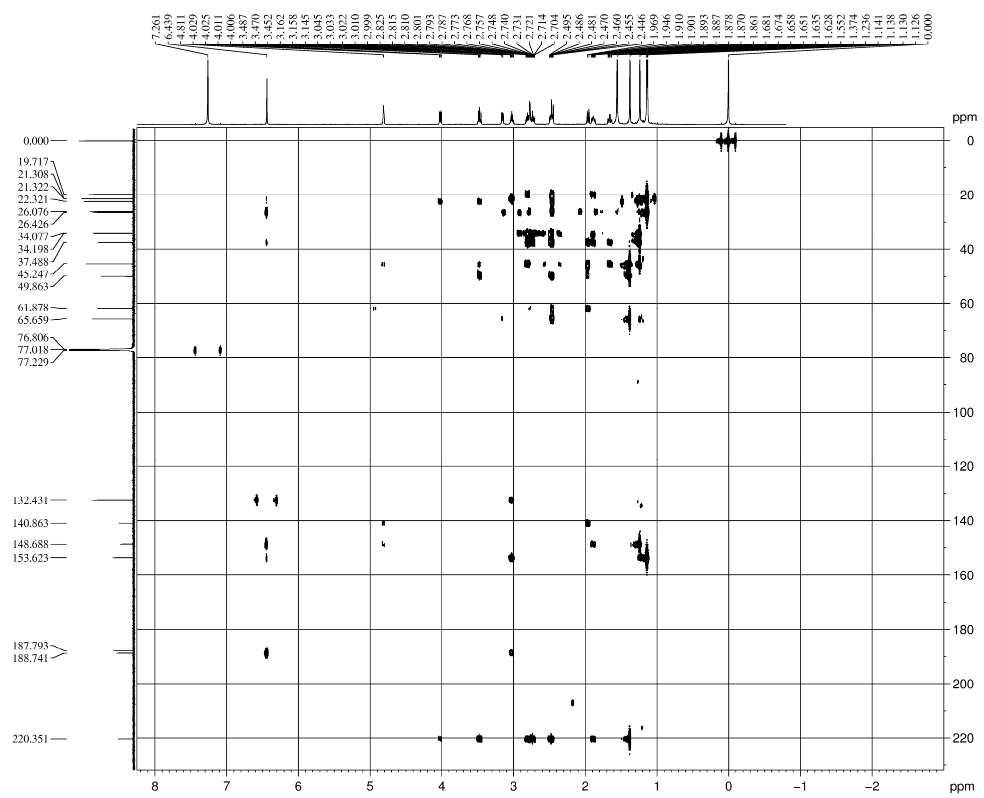

**Figure S29.** HMBC spectrum of compound **3** in $CDCl_3$.

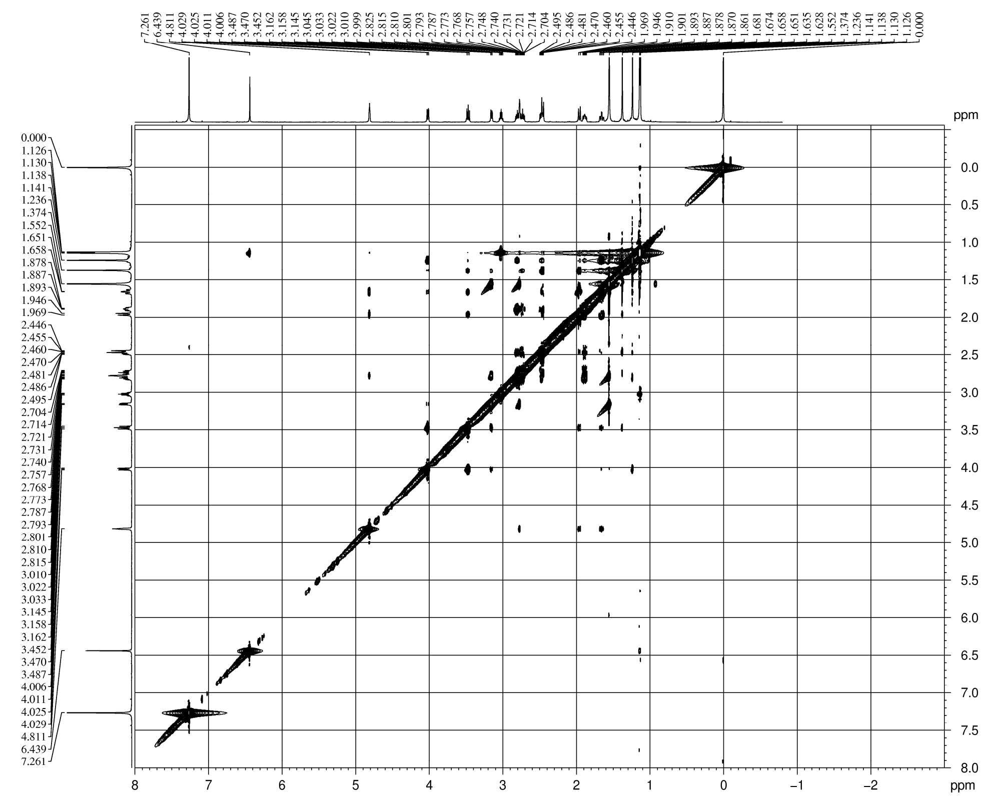

**Figure S30.** NOESY spectrum of compound **3** in $CDCl_3$.

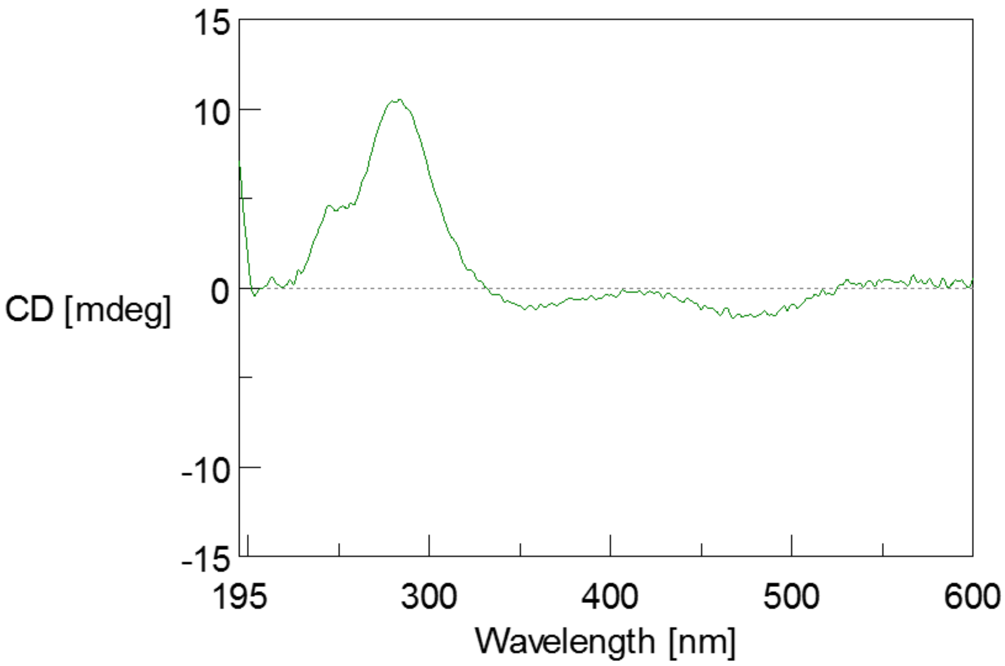


**Figure S31.** CD spectrum of compound **3** in MeOH.

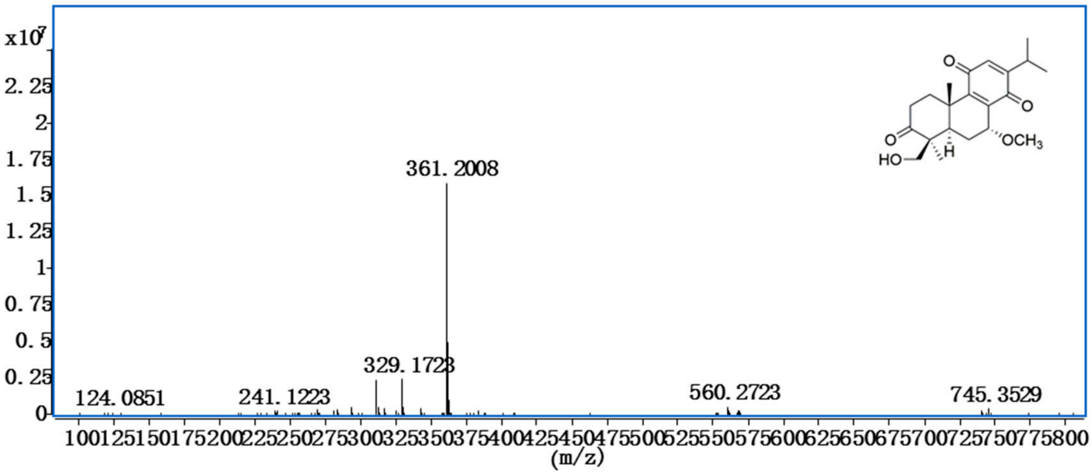


**Figure S32.** HRESIMS spectrum of compound **4**.

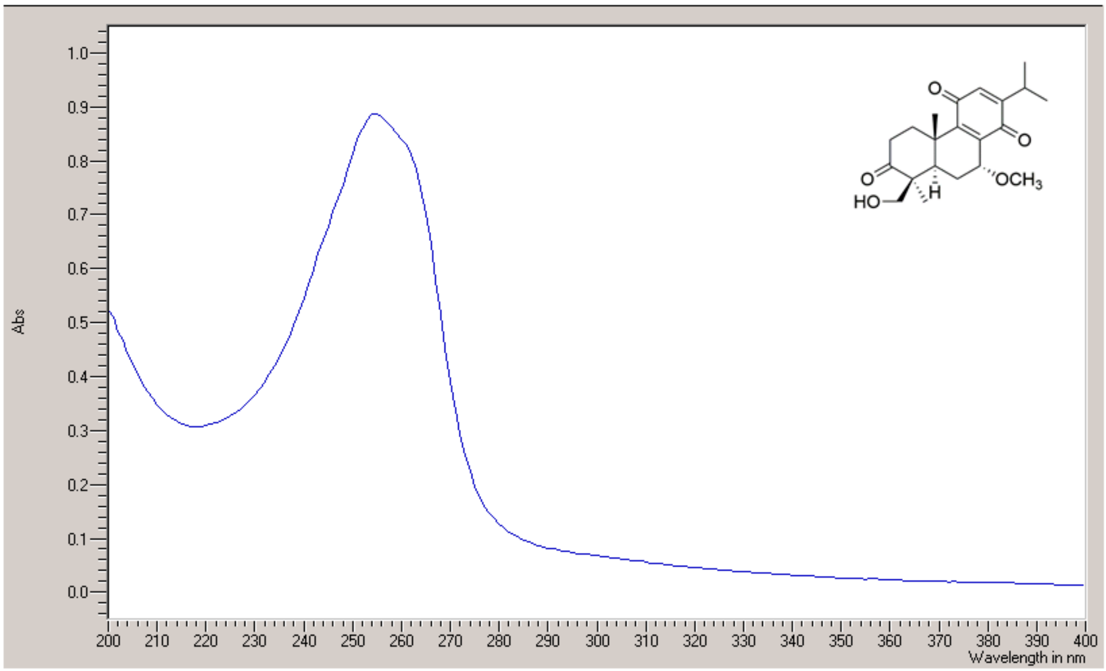


**Figure S33.** UV spectrum of compound **4** in MeOH.

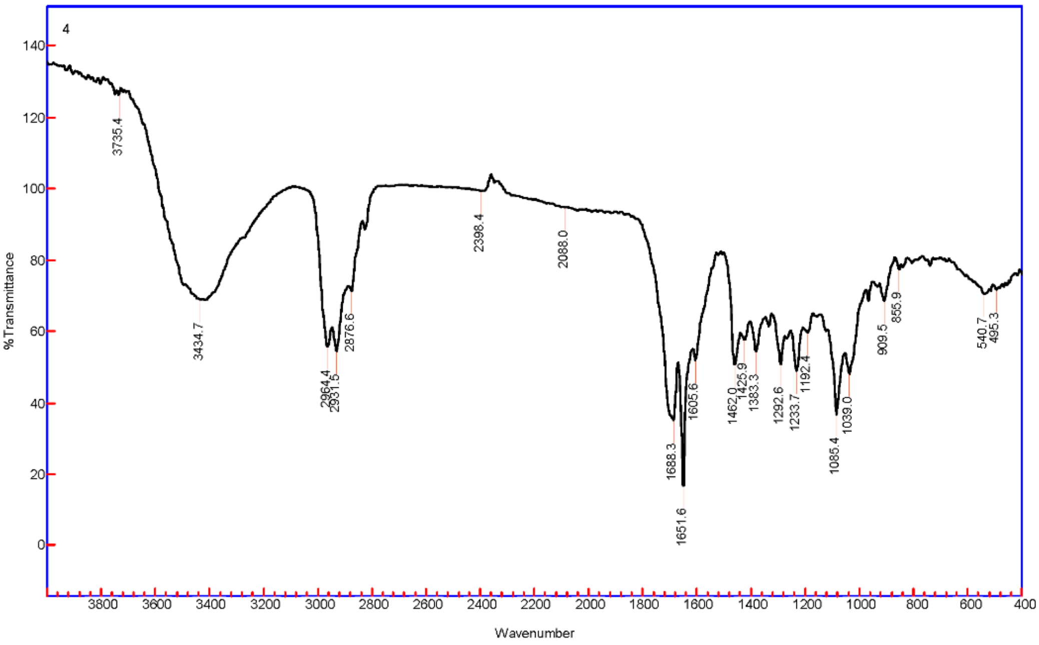


**Figure S34.** IR spectrum of compound **4**.

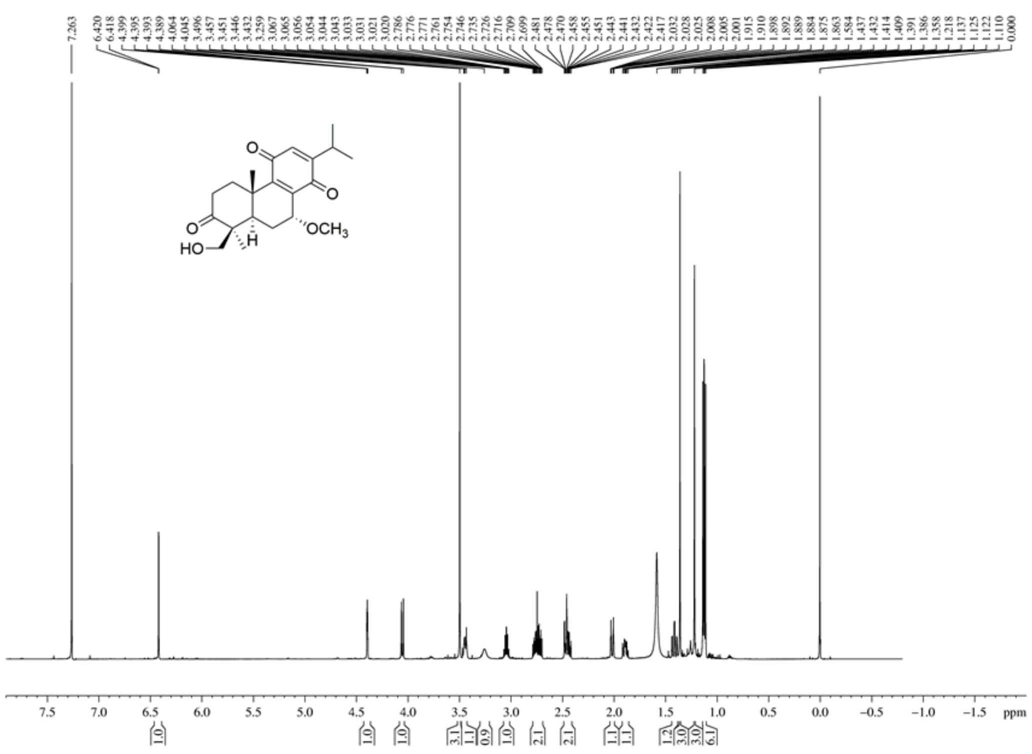

**Figure S35.** $^{1}H$ NMR spectrum of compound **4** in $CDCl_3$.

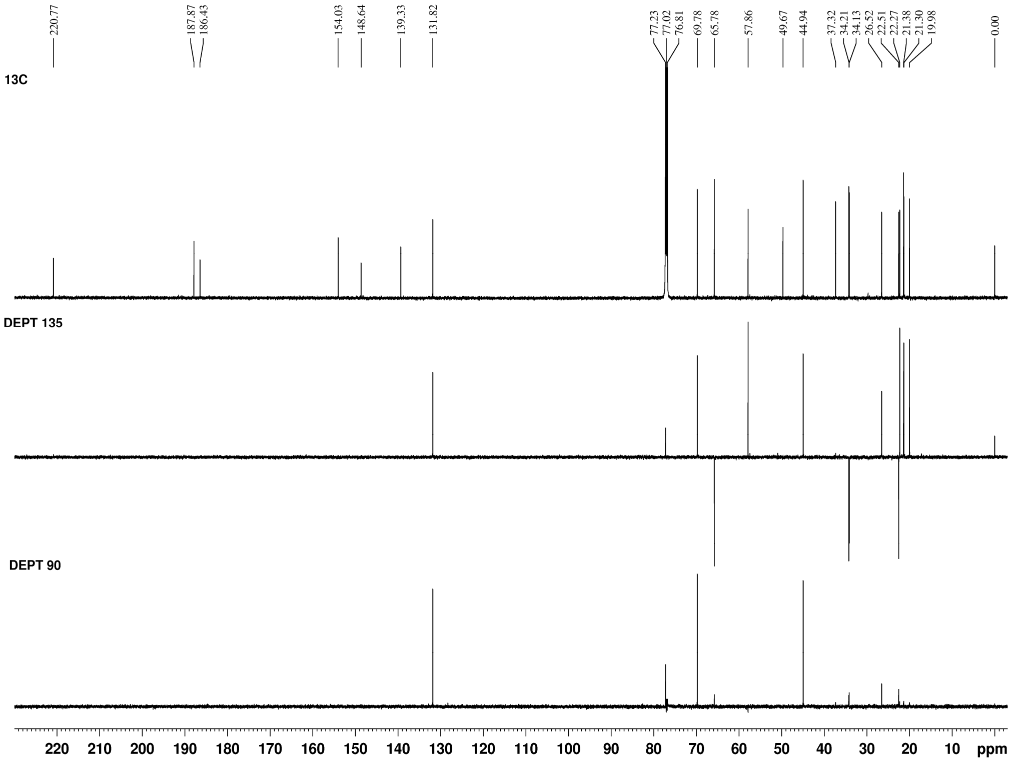


**Figure S36.** $^{13}C$ and DEPT NMR spectra of compound **4** in $CDCl_3$.

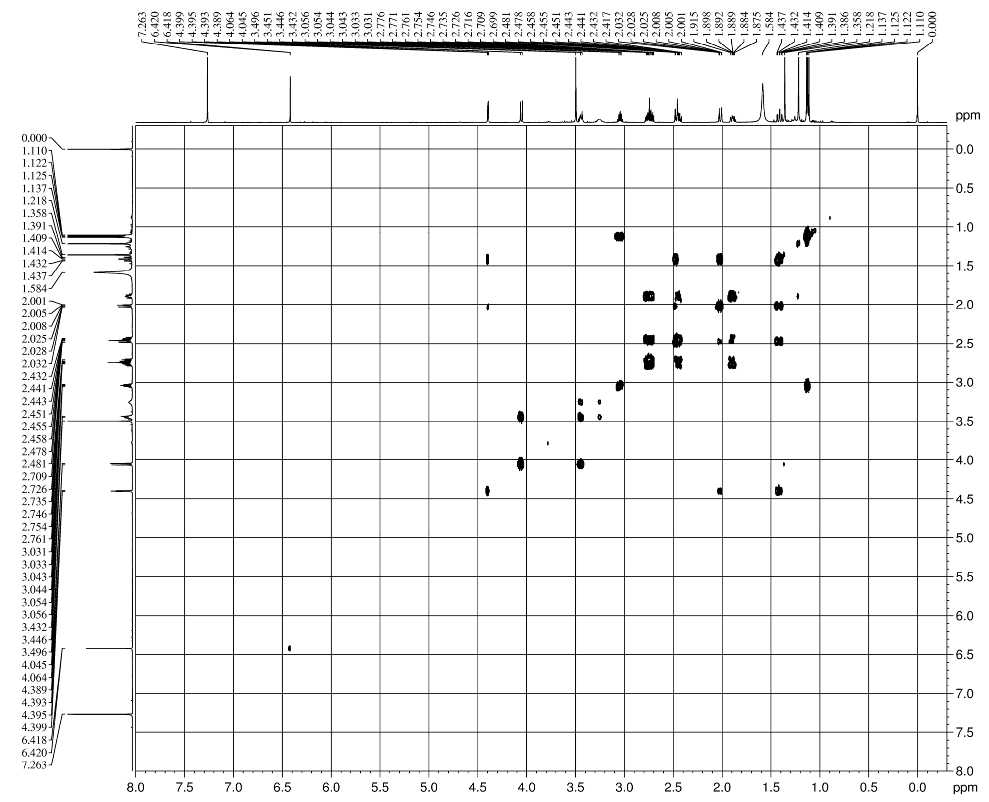

**Figure S37.** $^{1}$H–$^{1}$H COSY spectrum of compound **4** in $CDCl_3$.

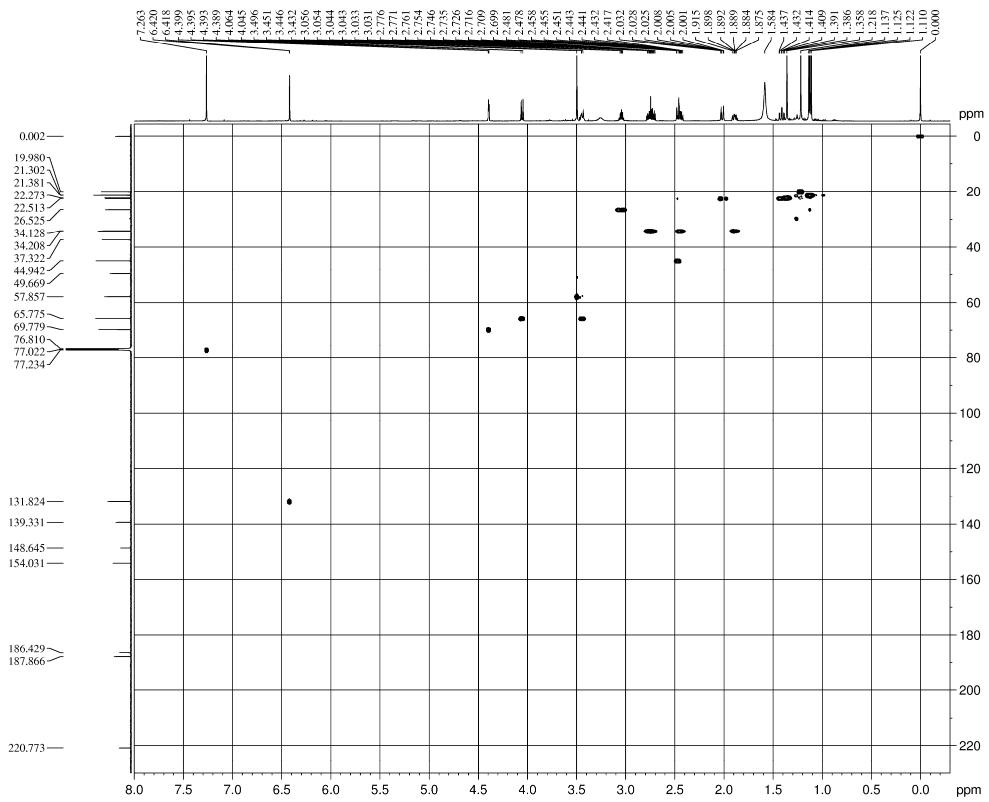

**Figure S38.** HSQC spectrum of compound **4** in $CDCl_3$.

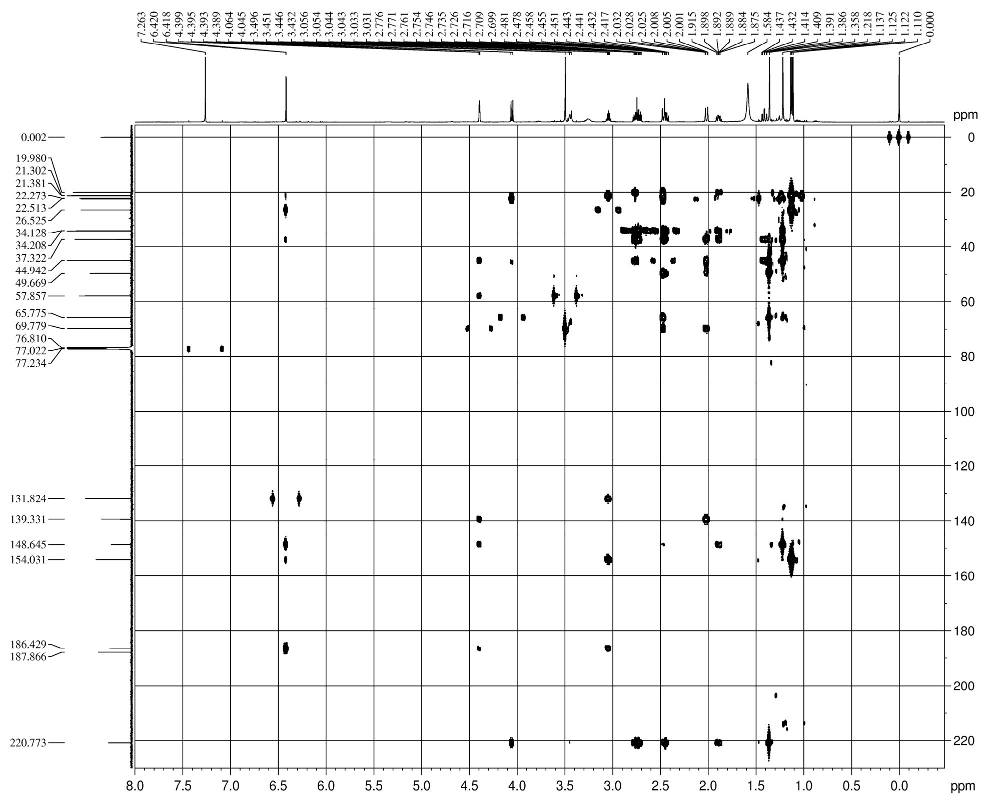

**Figure S39.** HMBC spectrum of compound **4** in $CDCl_3$.

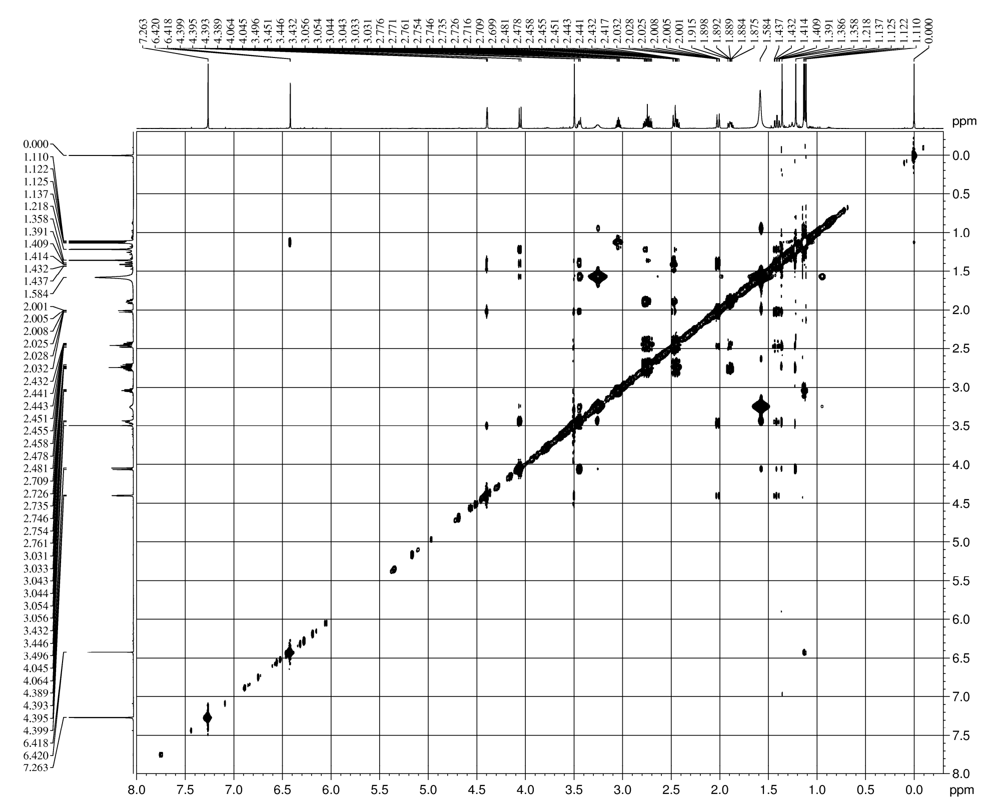

**Figure S40.** NOESY spectrum of compound **4** in $CDCl_3$

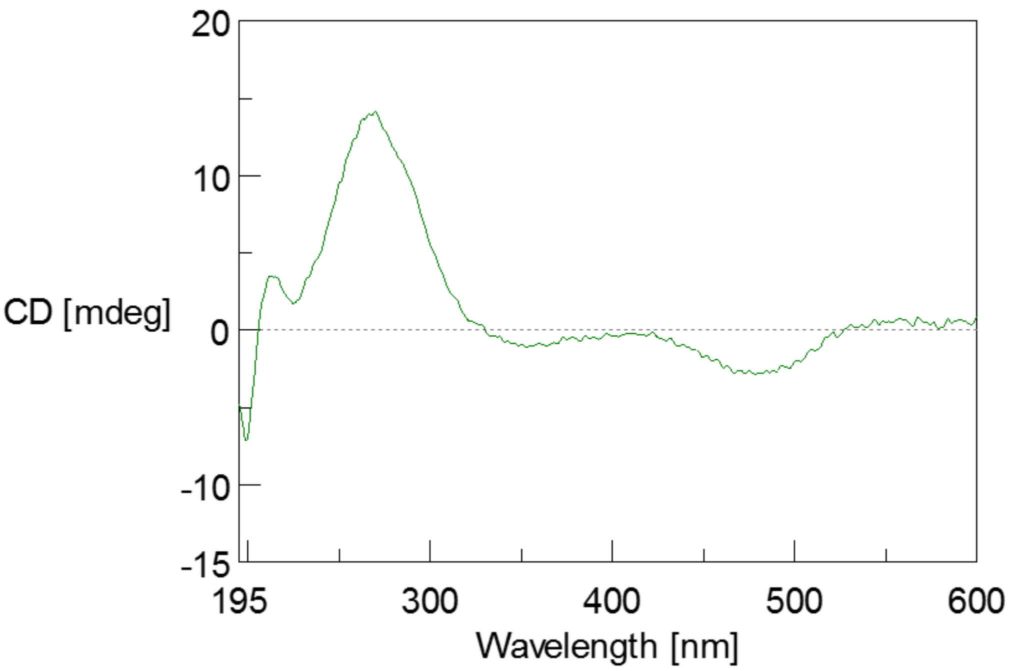


**Figure S41.** CD spectrum of compound **4** in MeOH.

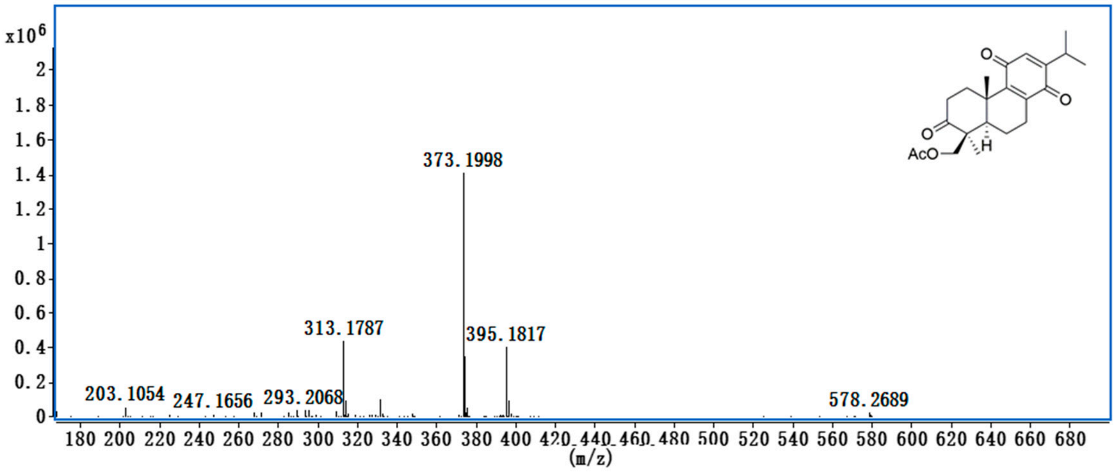


**Figure S42.** HRESIMS spectrum of compound **5**.

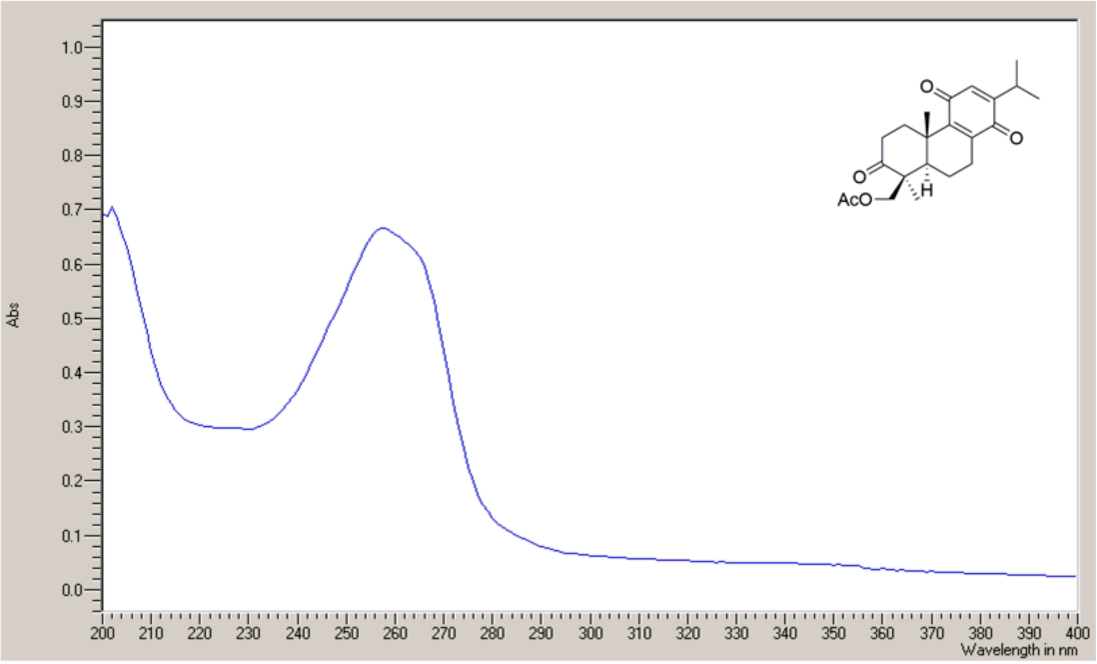


**Figure S43.** UV spectrum of compound **5** in MeOH.

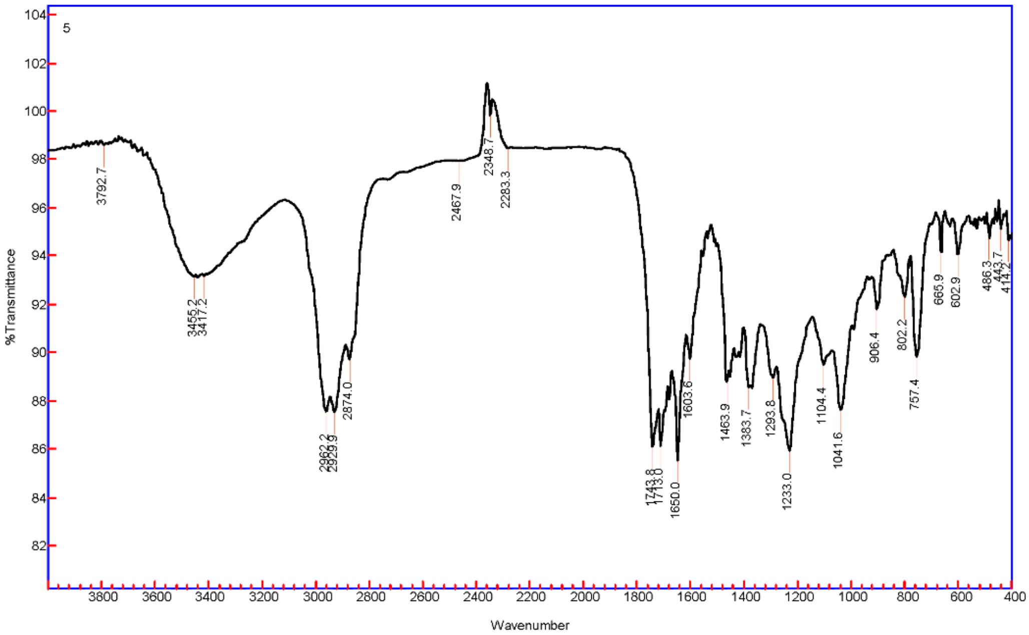


**Figure S44.** IR spectrum of compound **5**.

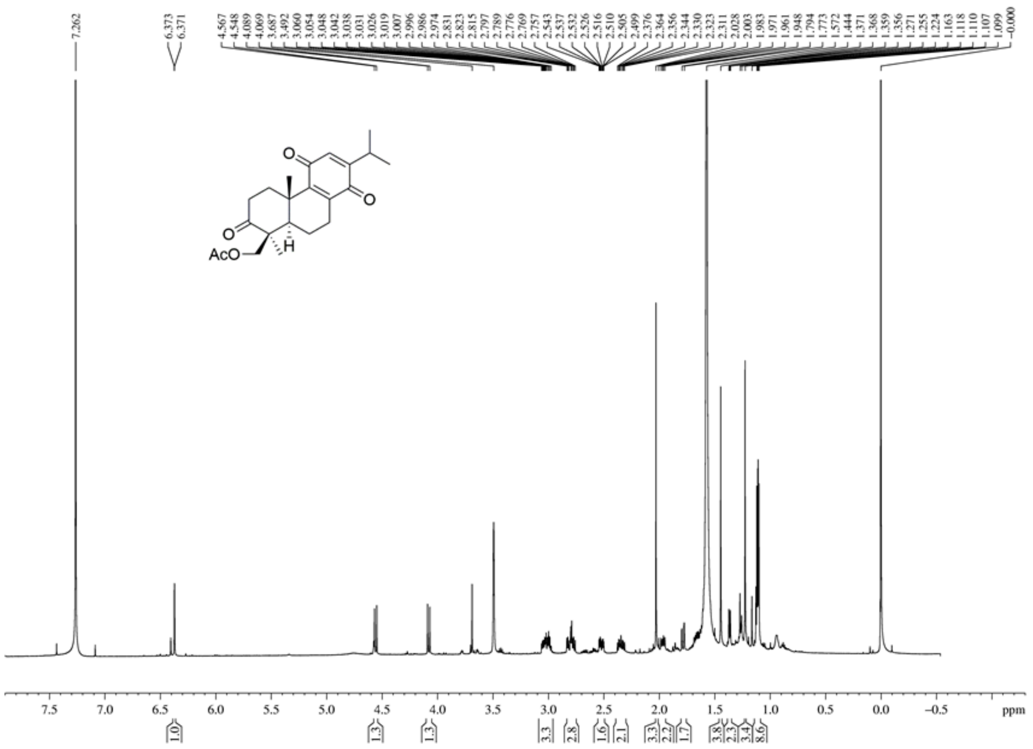


**Figure S45.** $^1$H NMR spectrum of compound **5** in $CDCl_3$.

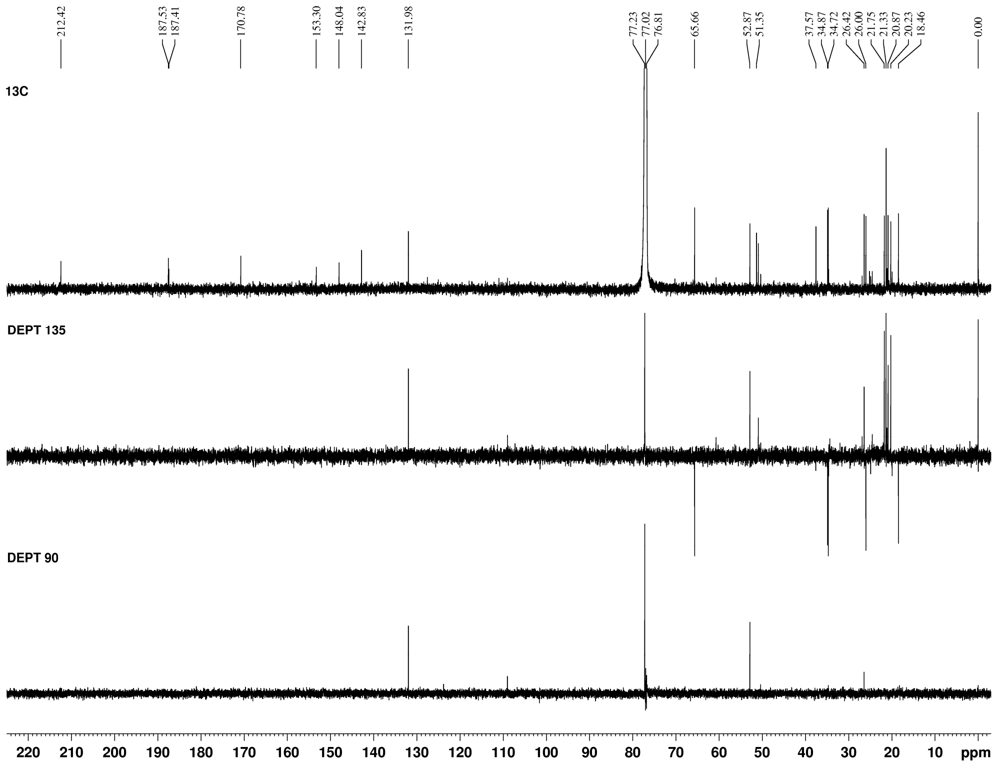


**Figure S46.** $^{13}$C and DEPT NMR spectra of compound **5** in $CDCl_3$.

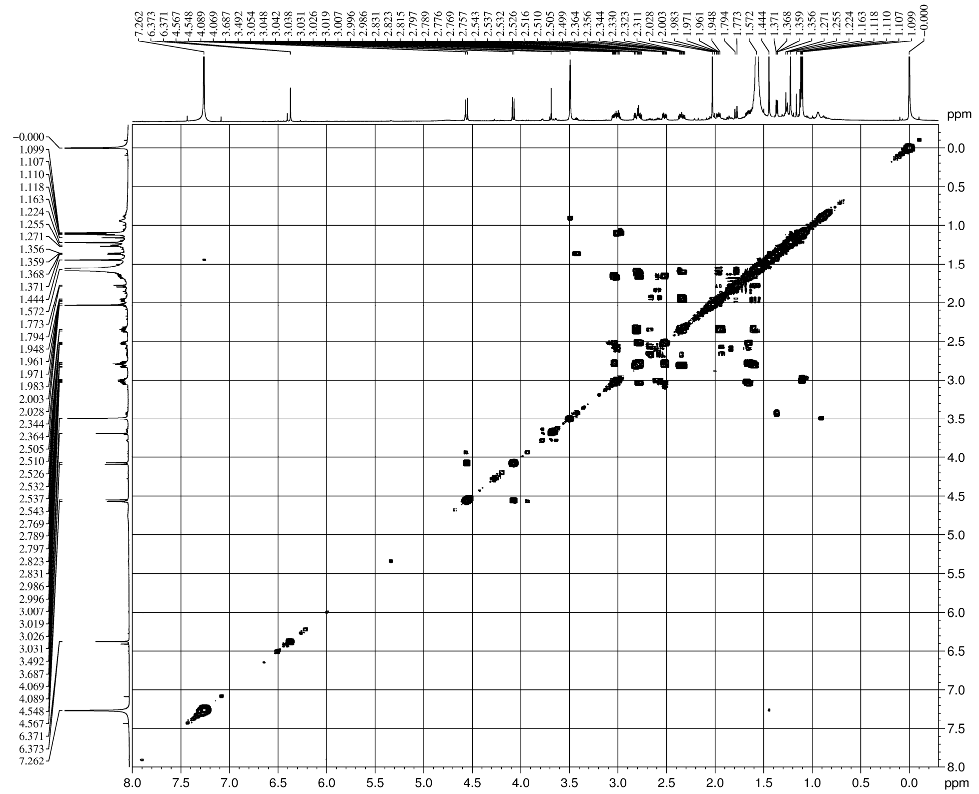


**Figure S47.** $^1H$–$^1H$ COSY spectrum of compound **5** in $CDCl_3$.

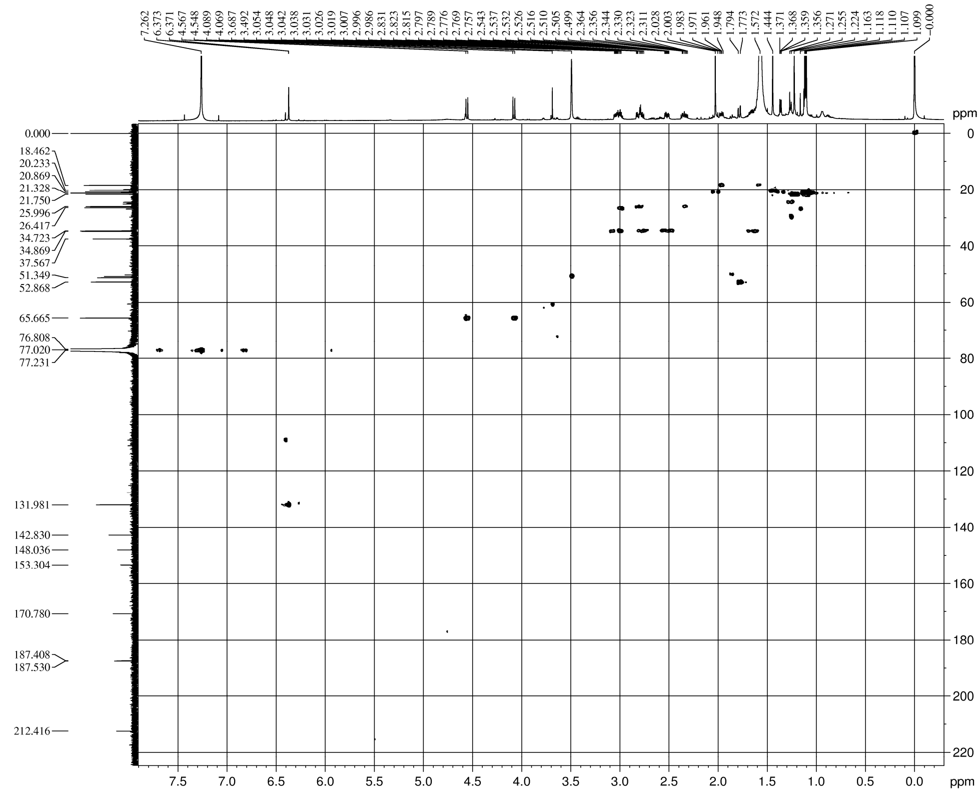


**Figure S48.** HSQC spectrum of compound **5** in $CDCl_3$.

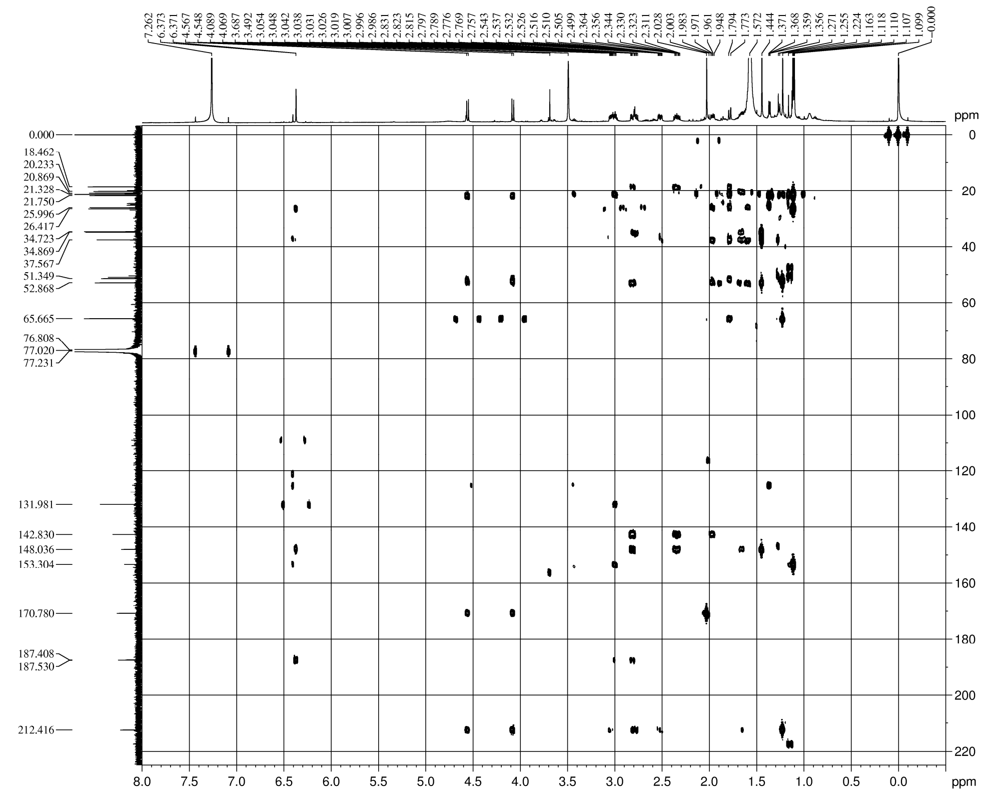

**Figure S49.** HMBC spectrum of compound **5** in $CDCl_3$.

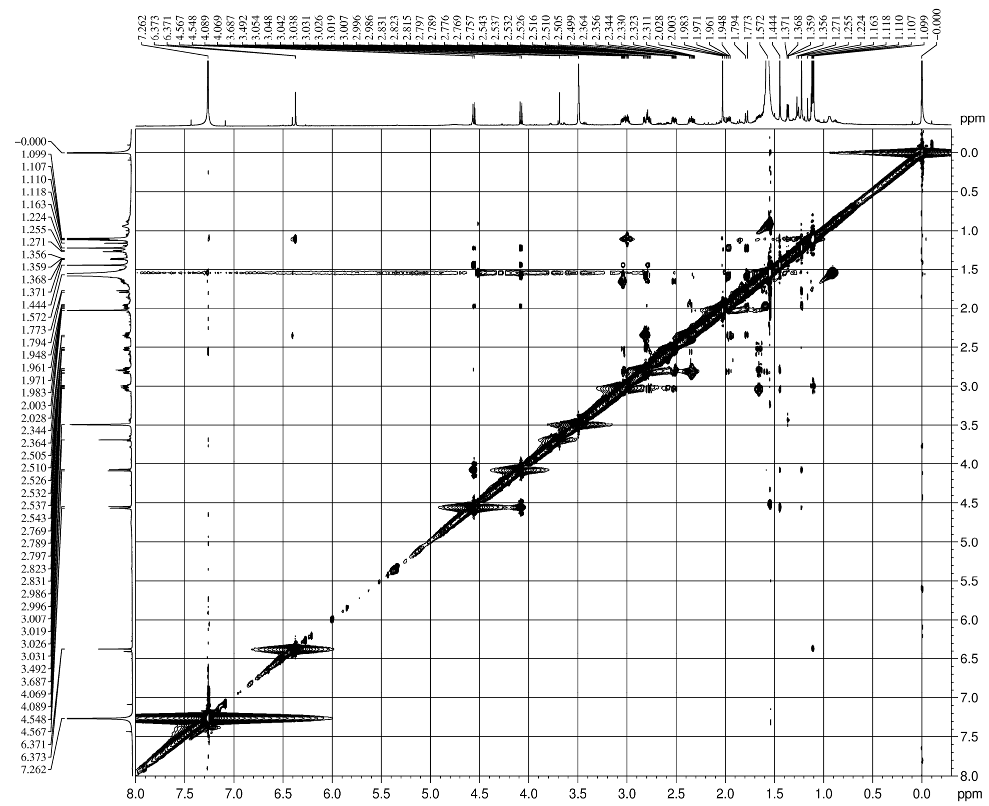

**Figure S50.** NOESY spectrum of compound **5** in $CDCl_3$.

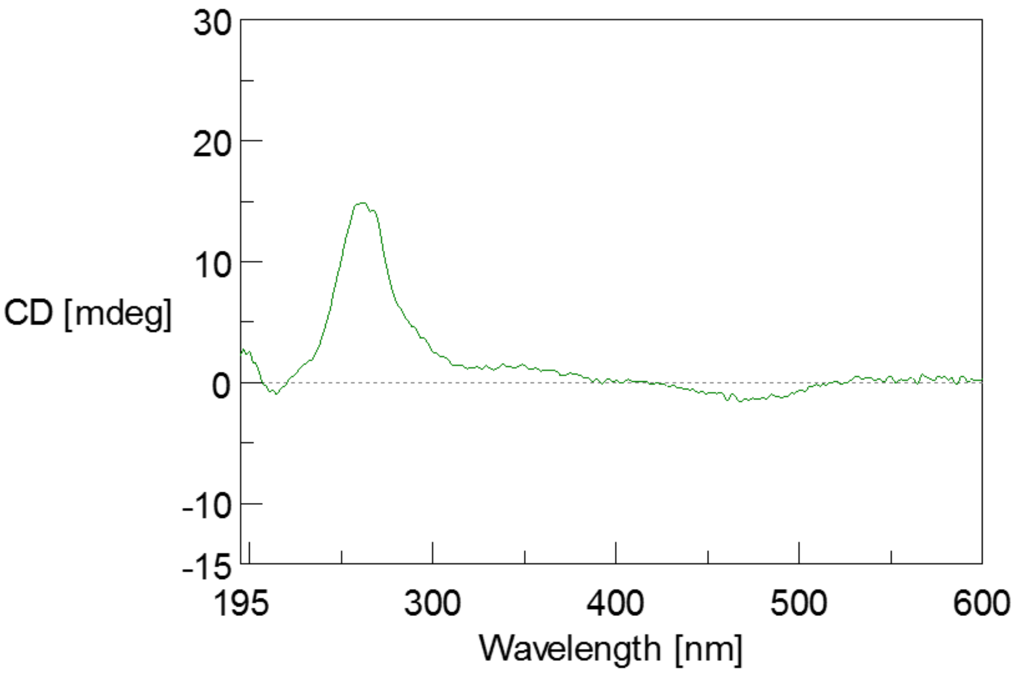


**Figure S51.** CD spectrum of compound **5** in MeOH.

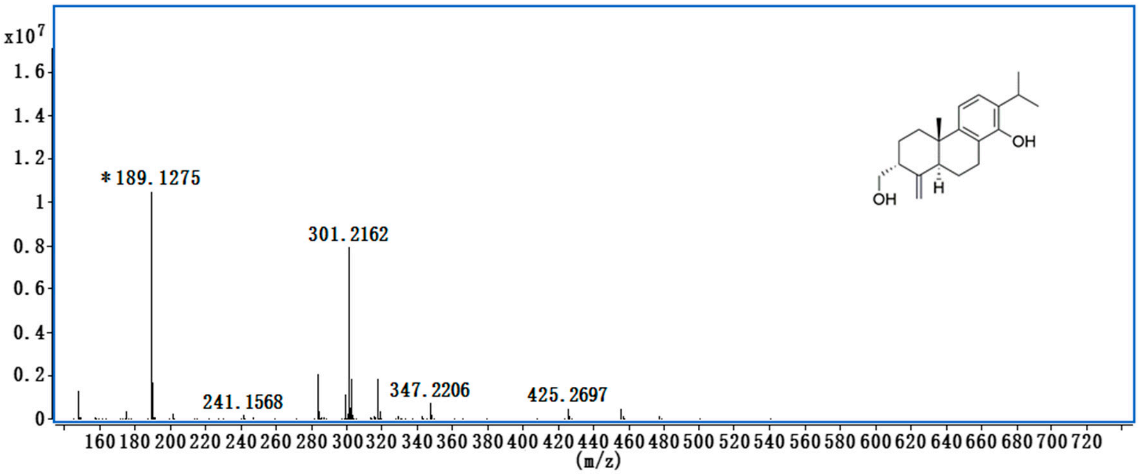


**Figure S52.** HRESIMS spectrum of compound **6**.

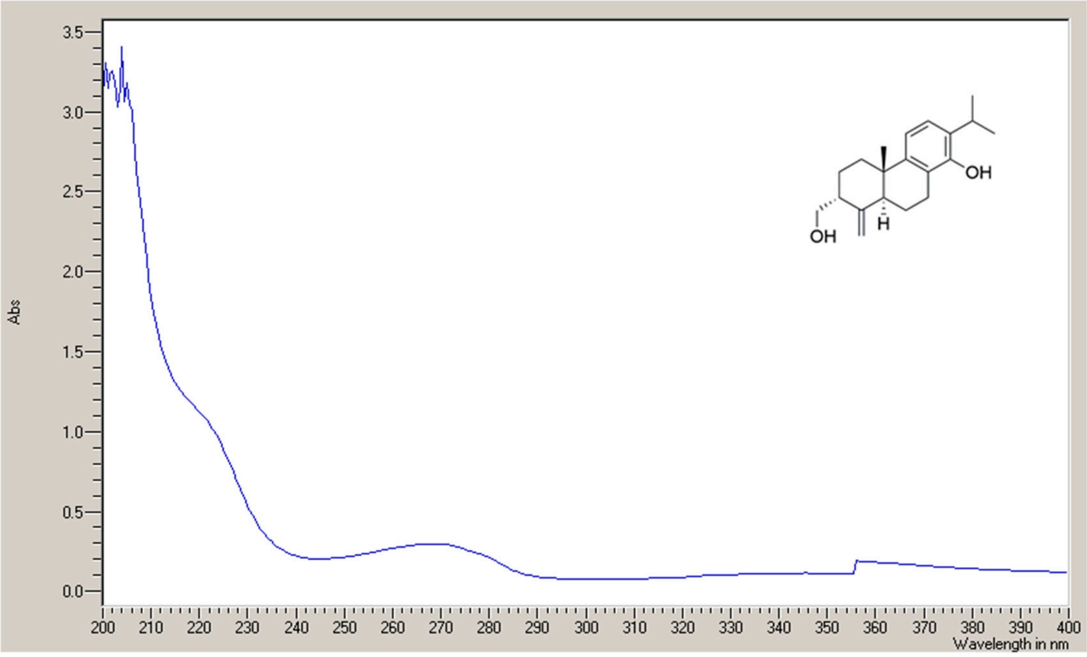


**Figure S53.** UV spectrum of compound **6** in MeOH.

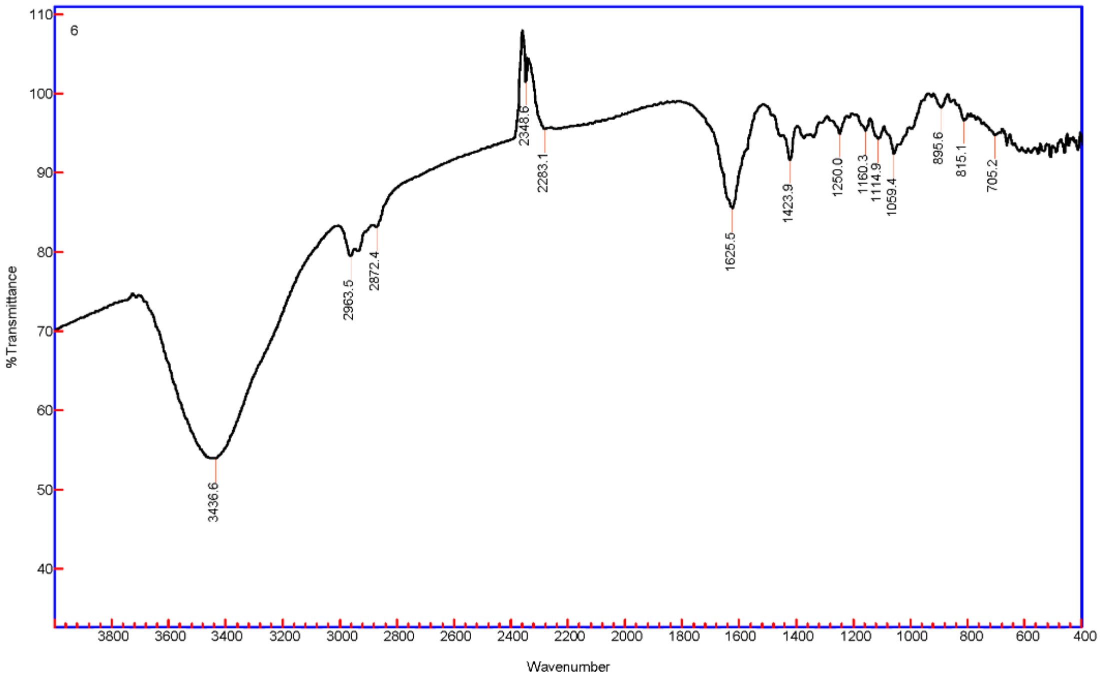


**Figure S54.** IR spectrum of compound **6**.

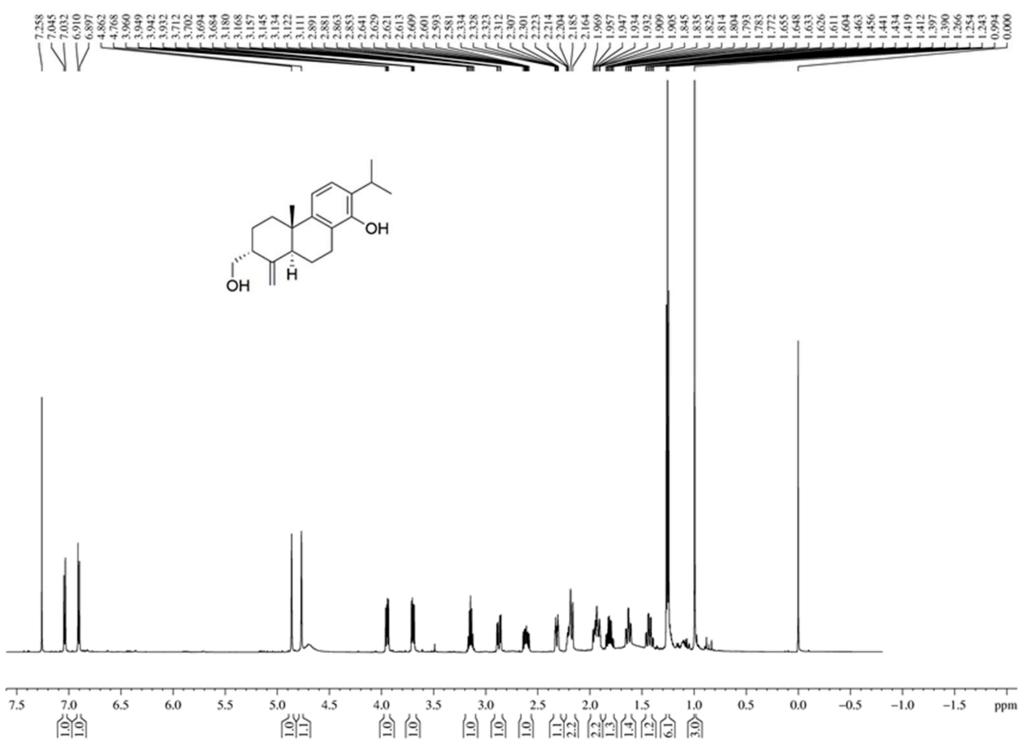


**Figure S55.** $^1H$ NMR spectrum of compound **6** in $CDCl_3$.

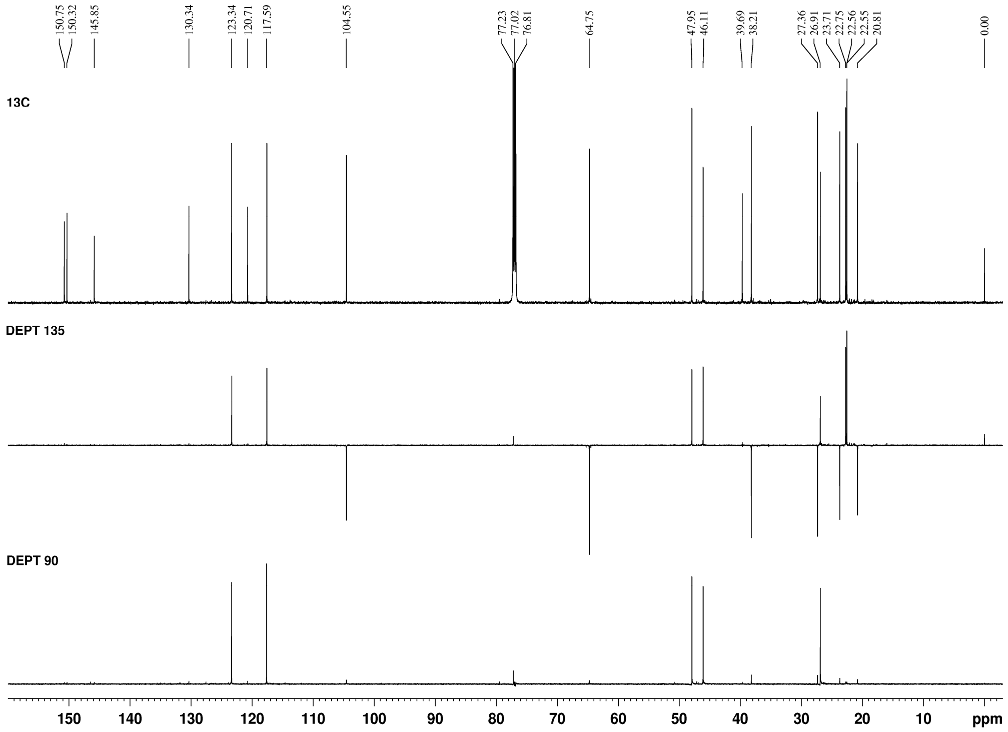


**Figure S56.** $^{13}C$ and DEPT NMR spectra of compound **6** in $CDCl_3$.

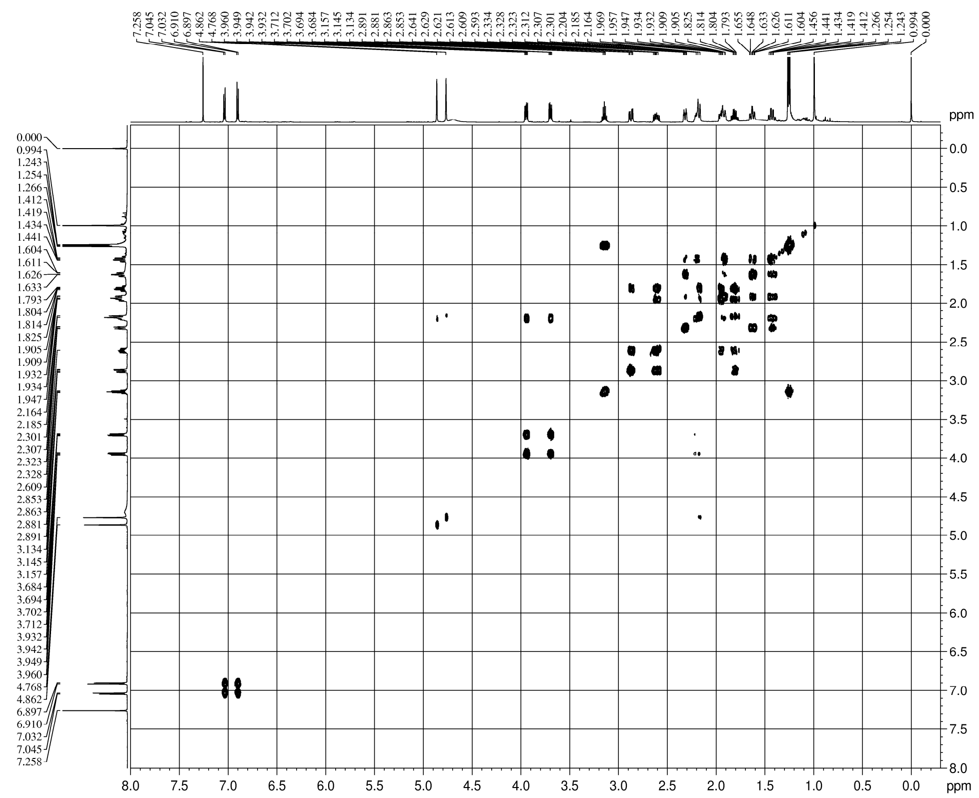


**Figure S57.** $^{1}$H–$^{1}$H COSY spectrum of compound **6** in $CDCl_3$.

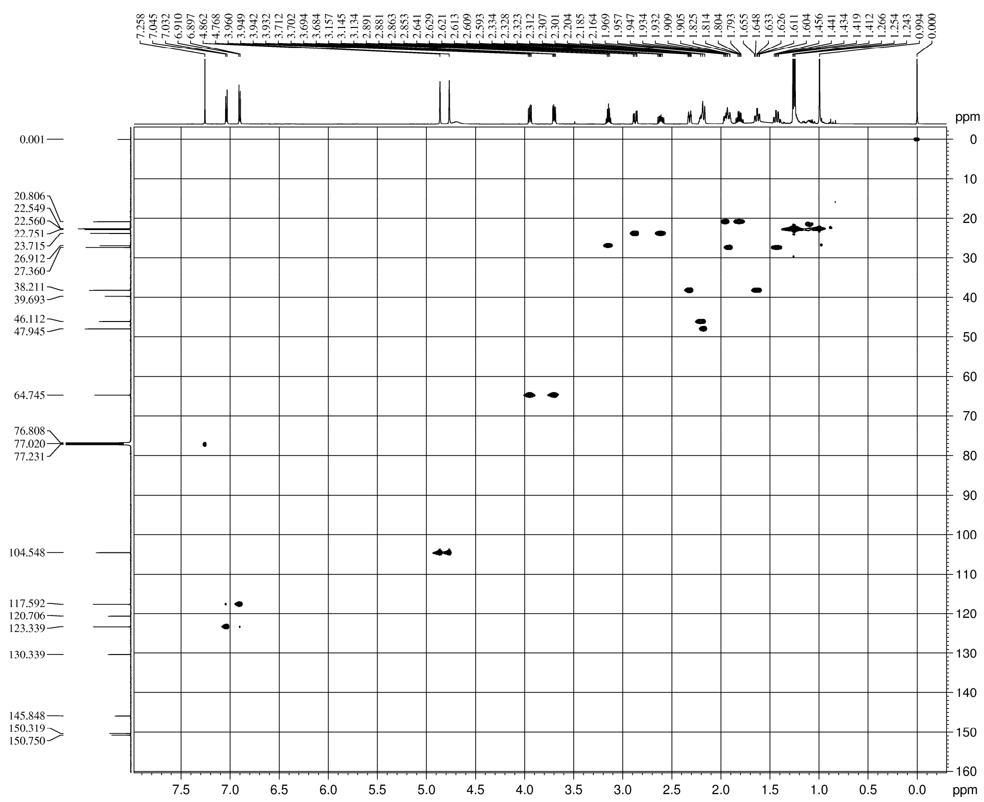


**Figure S58.** HSQC spectrum of compound **6** in $CDCl_3$.

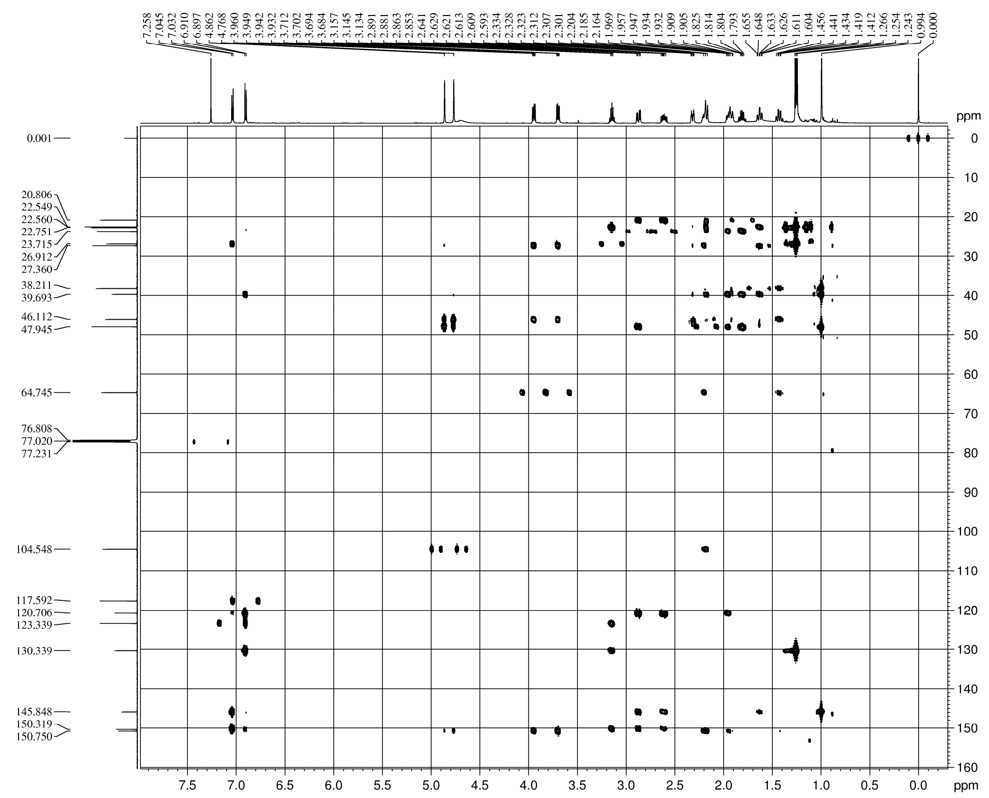

**Figure S59.** HMBC spectrum of compound **6** in $CDCl_3$.

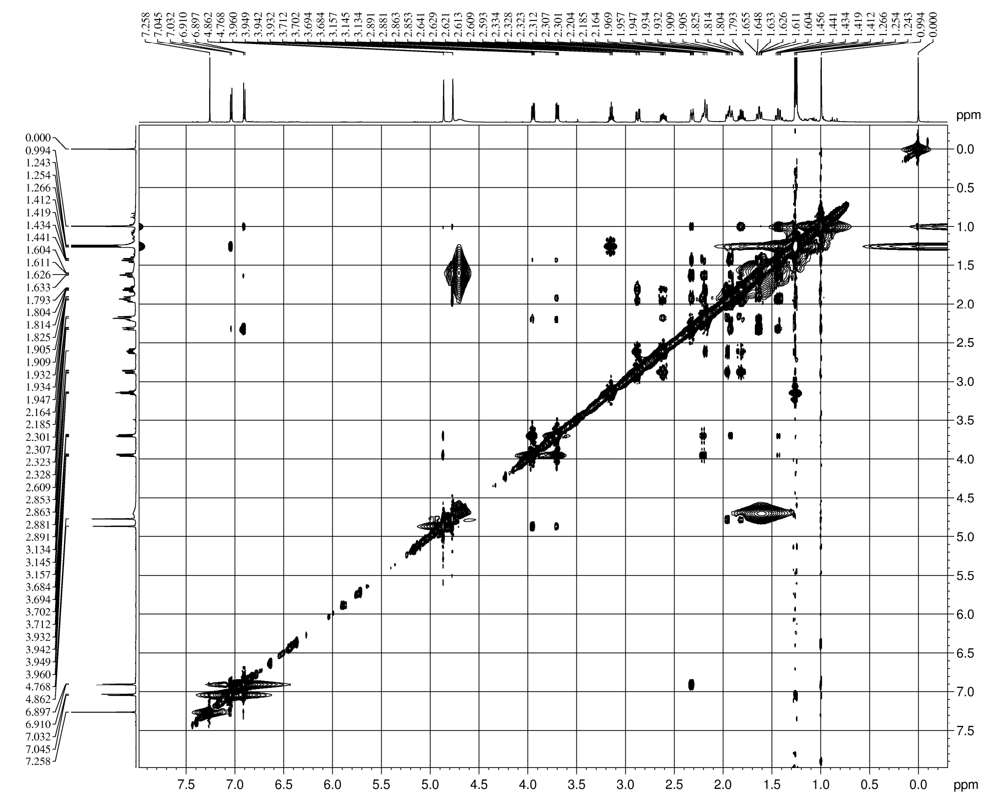

**Figure S60.** NOESY spectrum of compound **6** in $CDCl_3$.

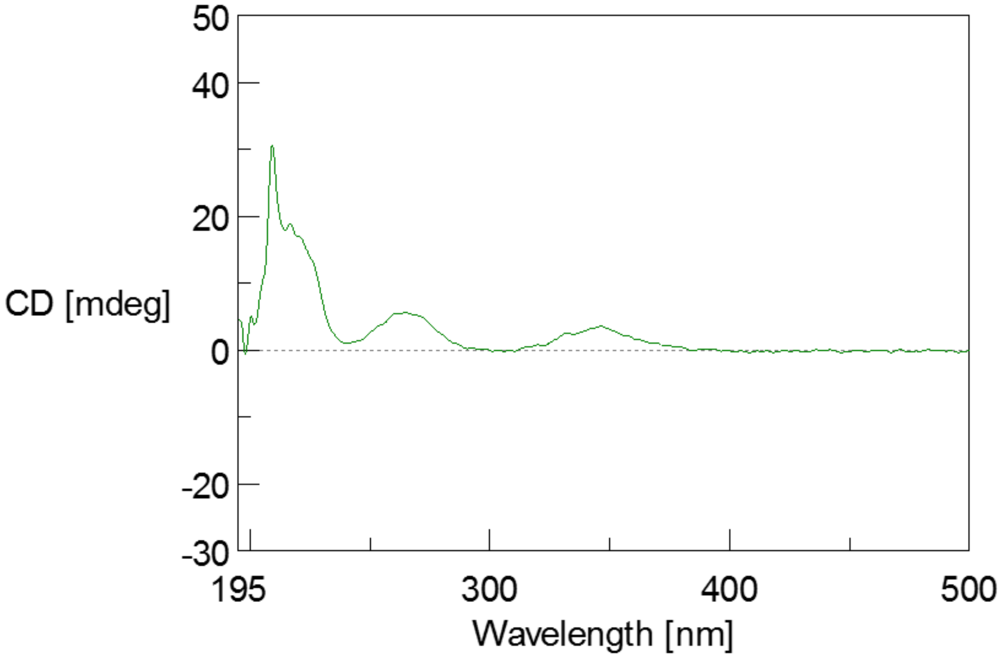


**Figure S61.** CD spectrum of compound **6** in MeOH.

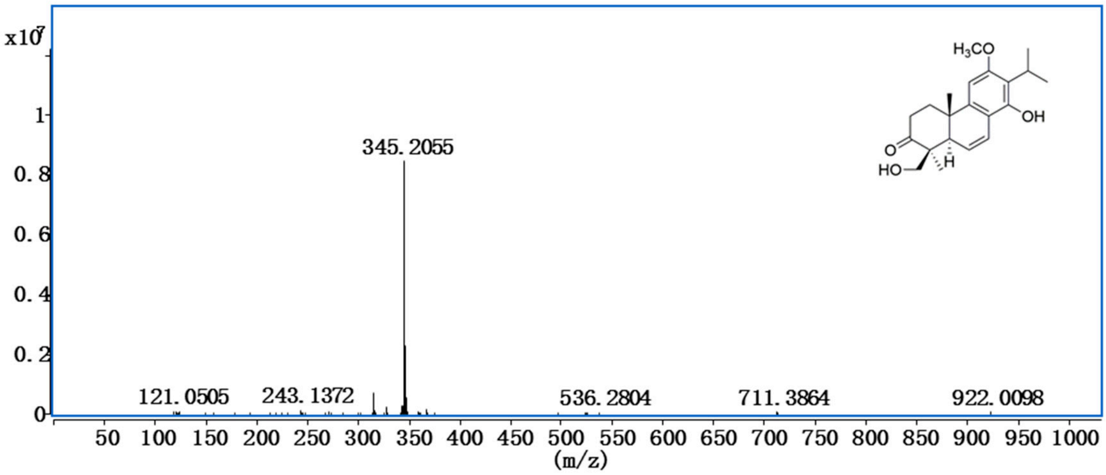


**Figure S62.** HRESIMS spectrum of compound **7**.

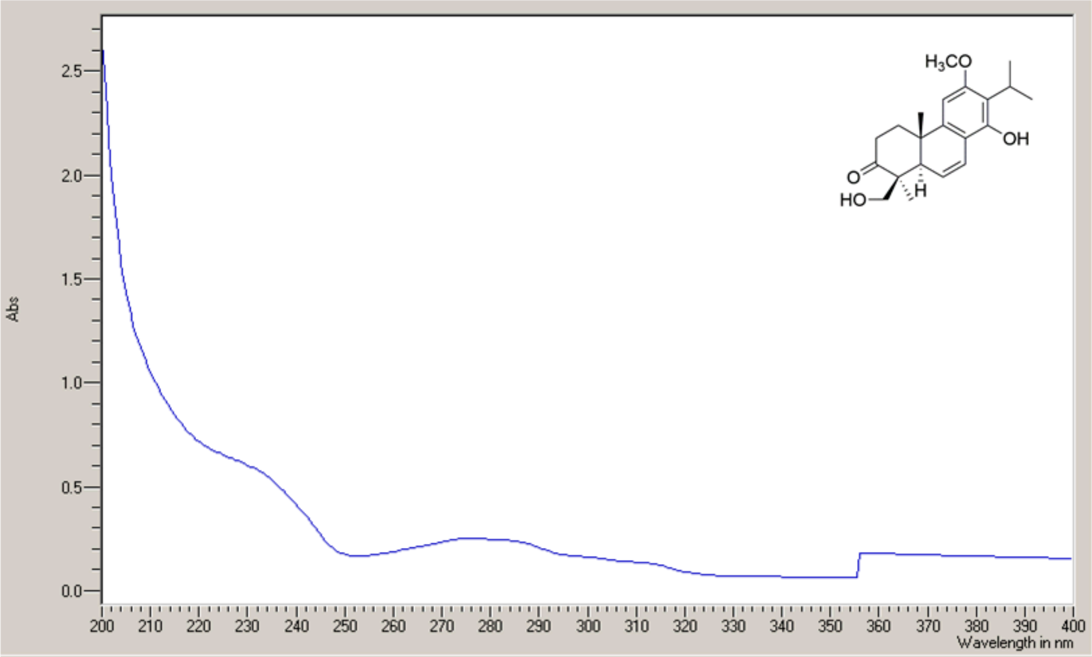


**Figure S63.** UV spectrum of compound **7** in MeOH.

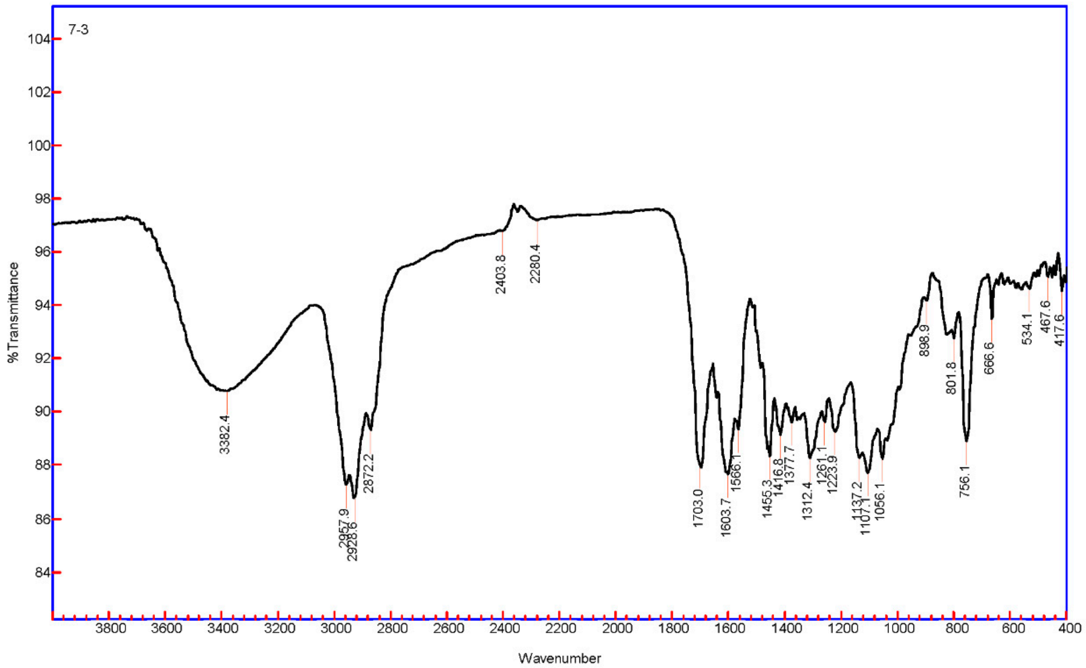


**Figure S64.** IR spectrum of compound **7**.

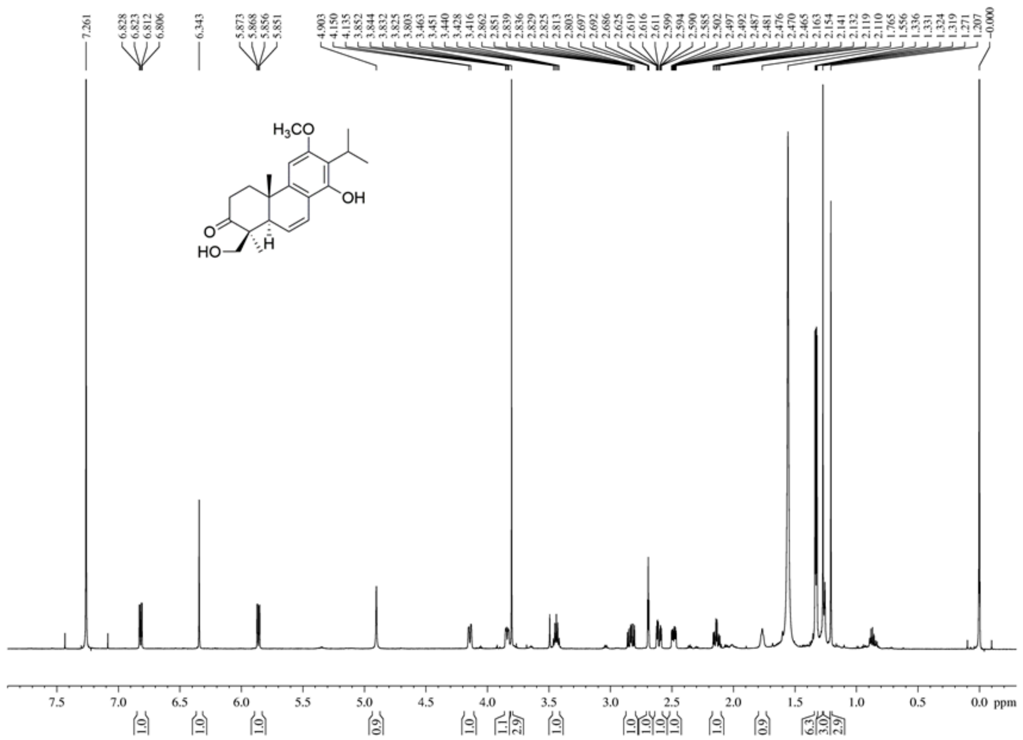

**Figure S65.** $^{1}H$ NMR spectrum of compound **7** in $CDCl_3$.

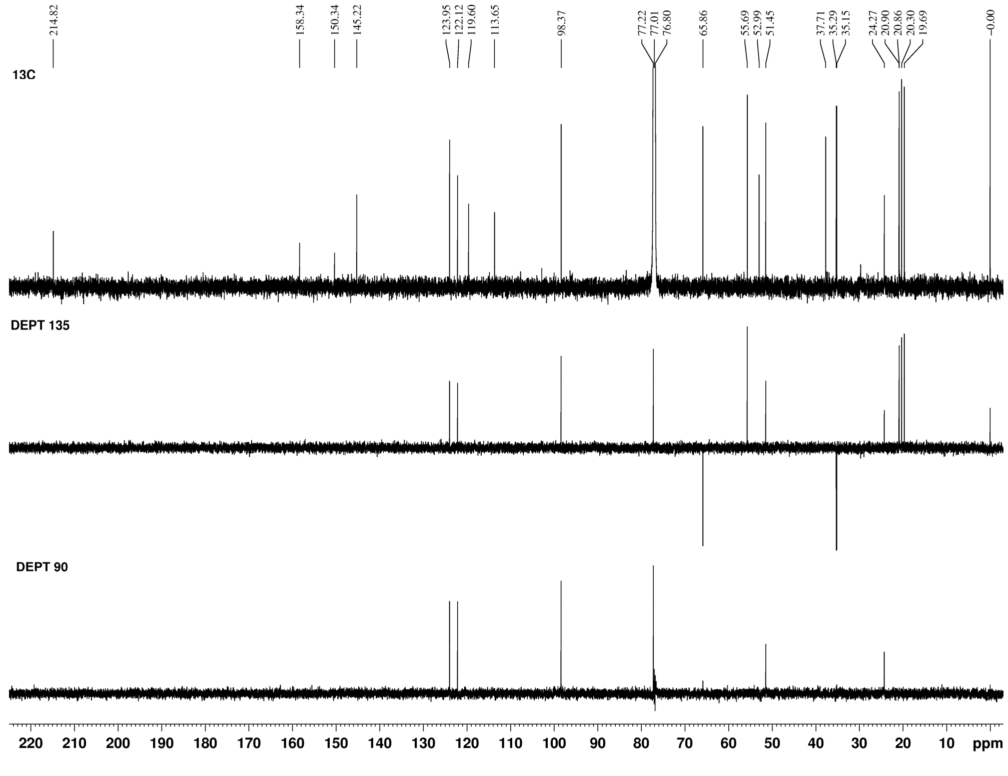

**Figure S66.** $^{13}C$ and DEPT NMR spectra of compound **7** in $CDCl_3$.

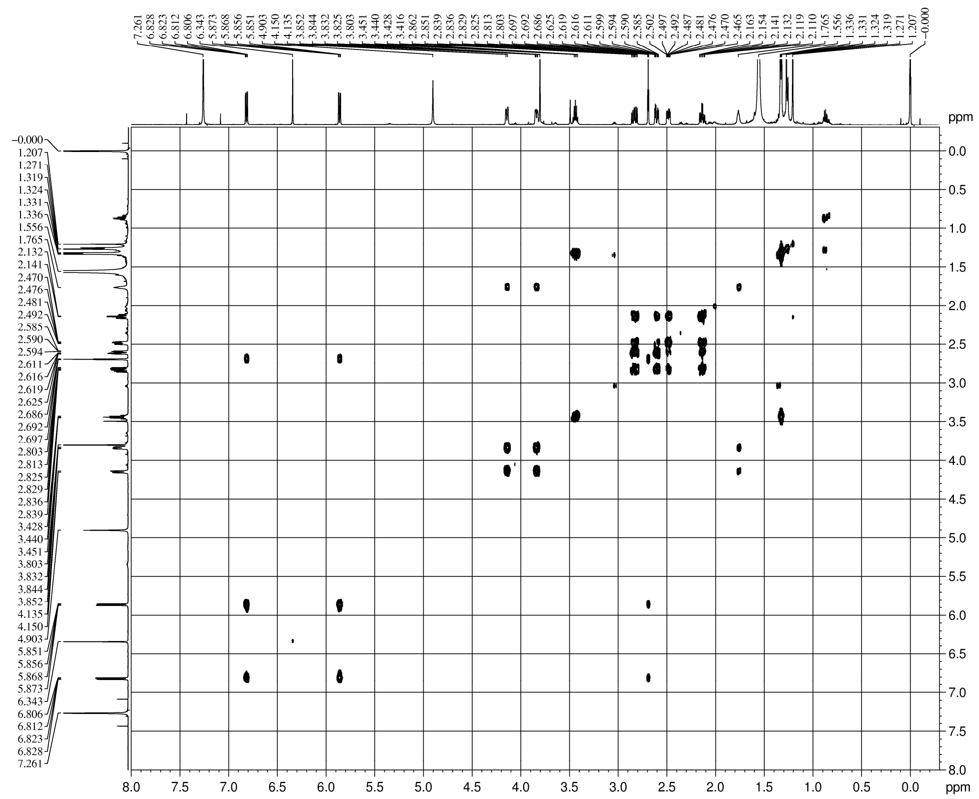

**Figure S67.** $^1$H–$^1$H COSY spectrum of compound **7** in $CDCl_3$.

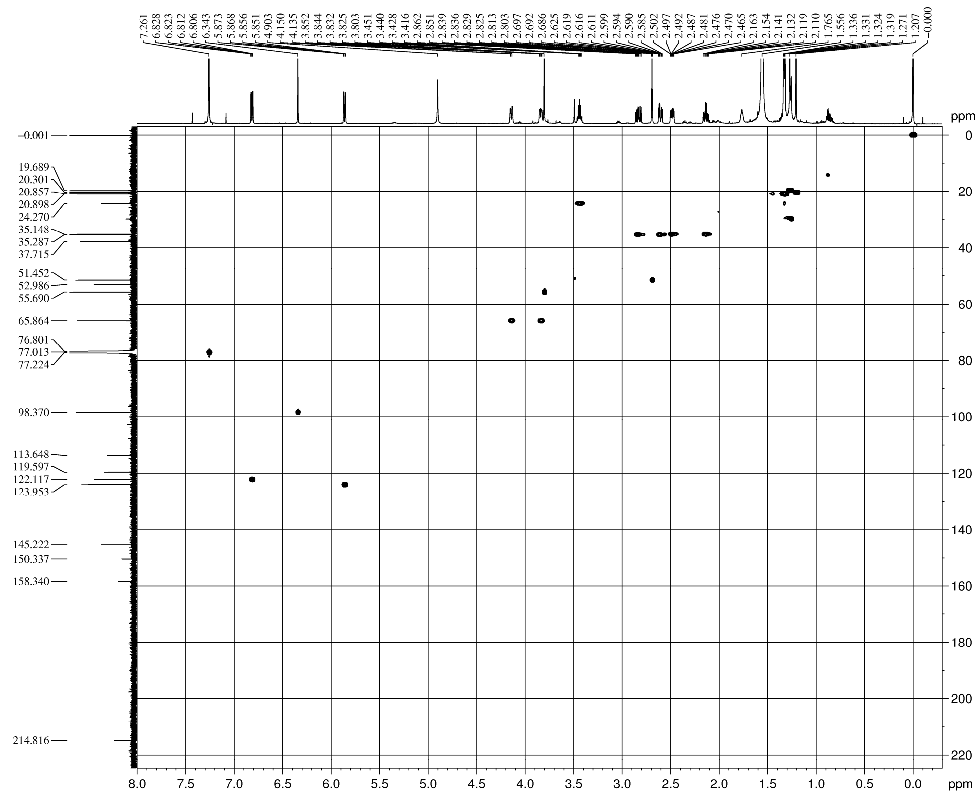

**Figure S68.** HSQC spectrum of compound **7** in $CDCl_3$.

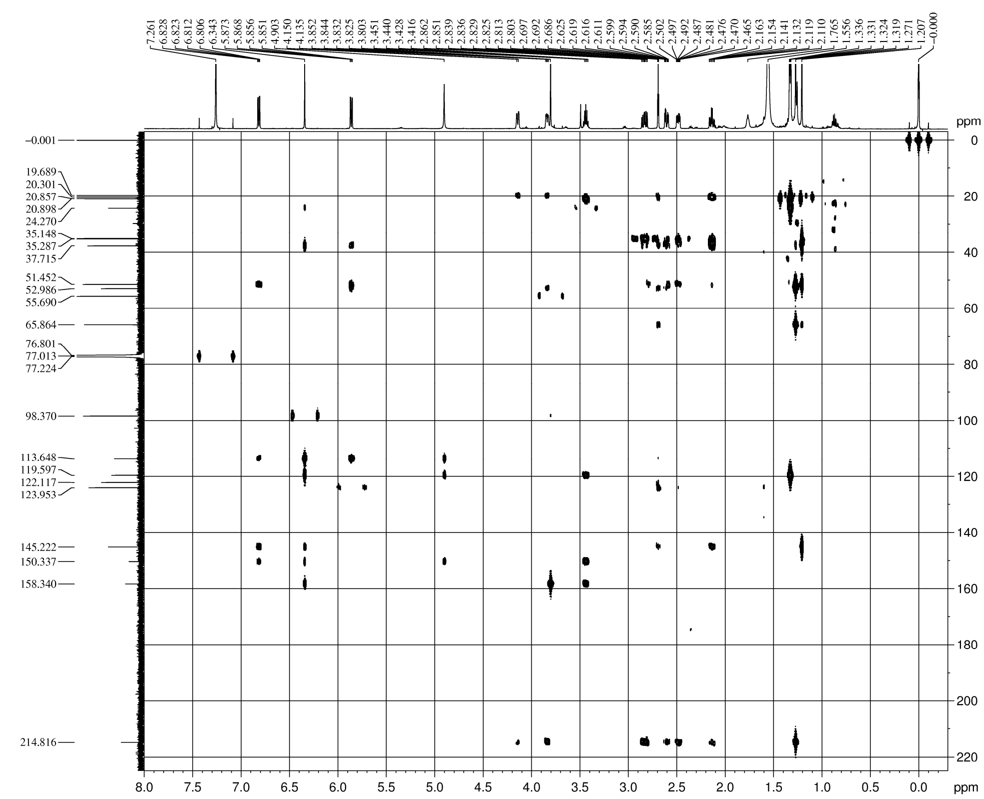

**Figure S69.** HMBC spectrum of compound **7** in $CDCl_3$.

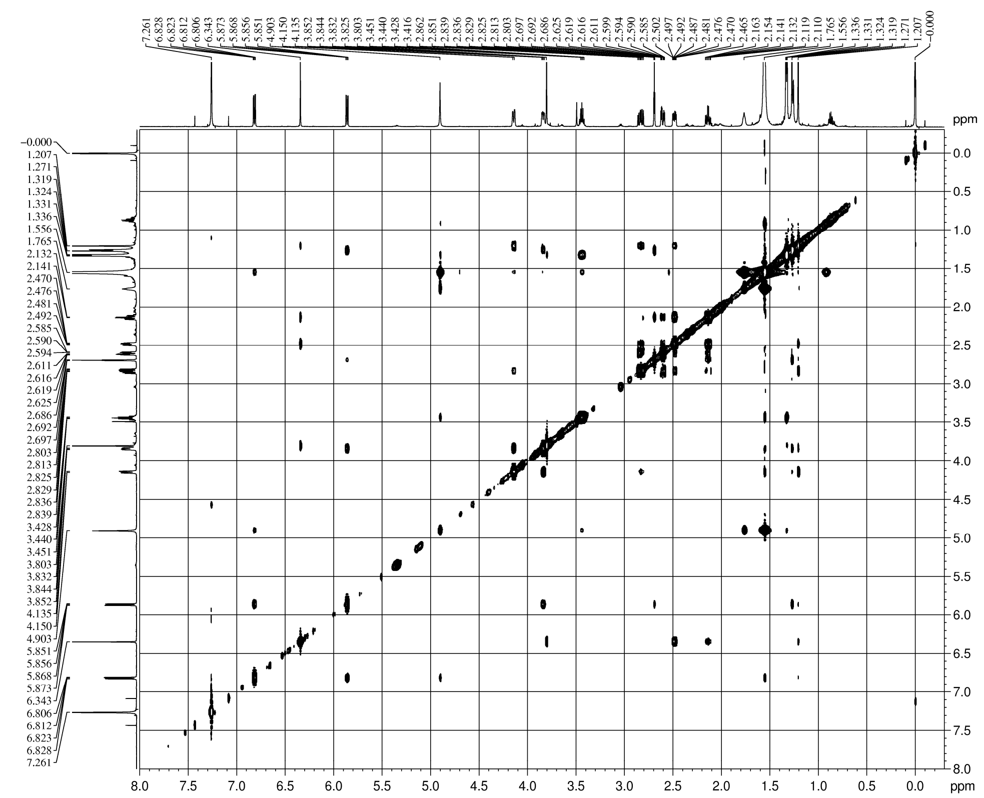

**Figure S70.** NOESY spectrum of compound **7** in $CDCl_3$.

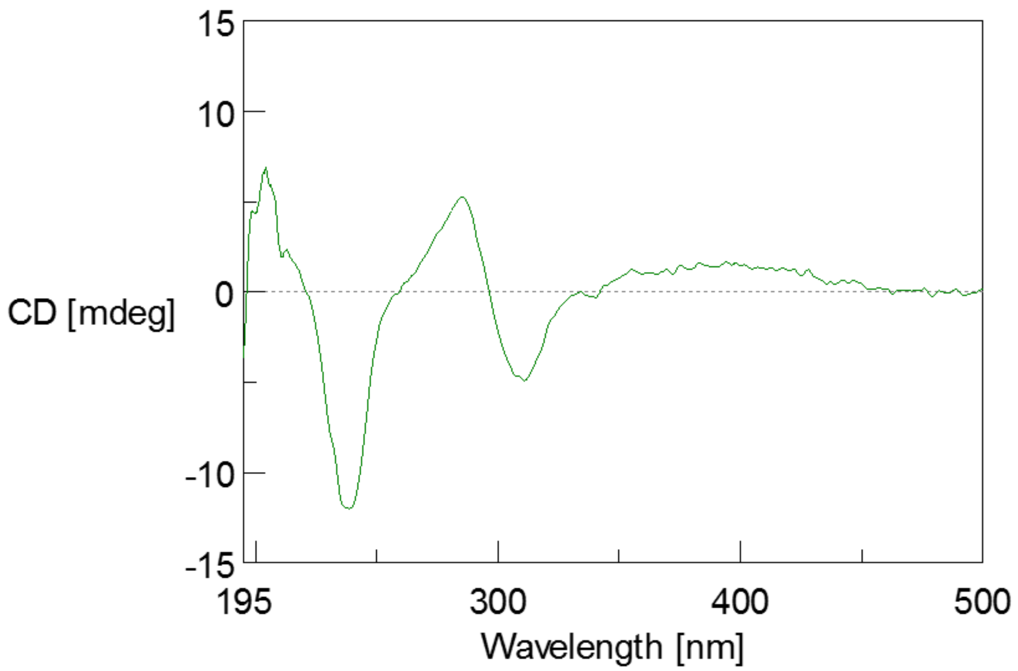


**Figure S71.** CD spectrum of compound **7** in MeOH.

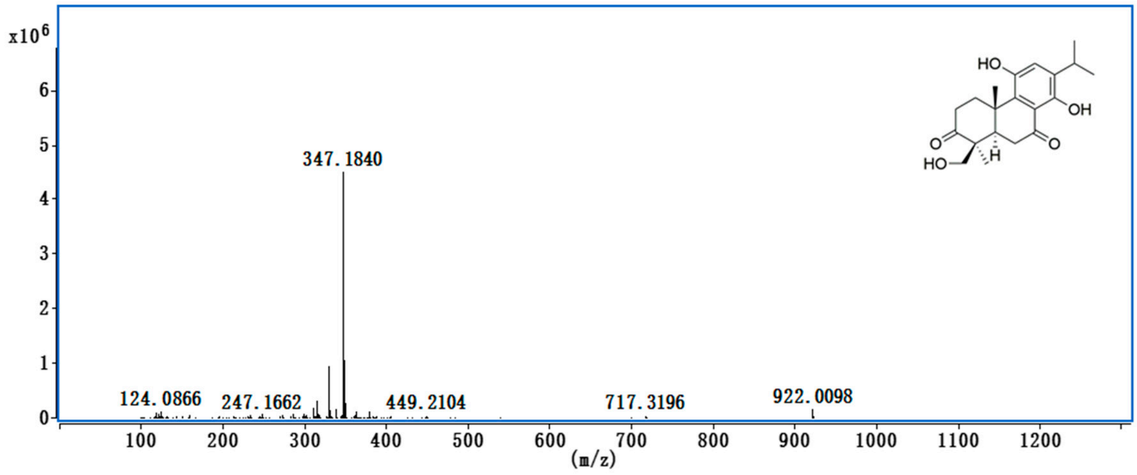


**Figure S72.** HRESIMS spectrum of compound **8**.

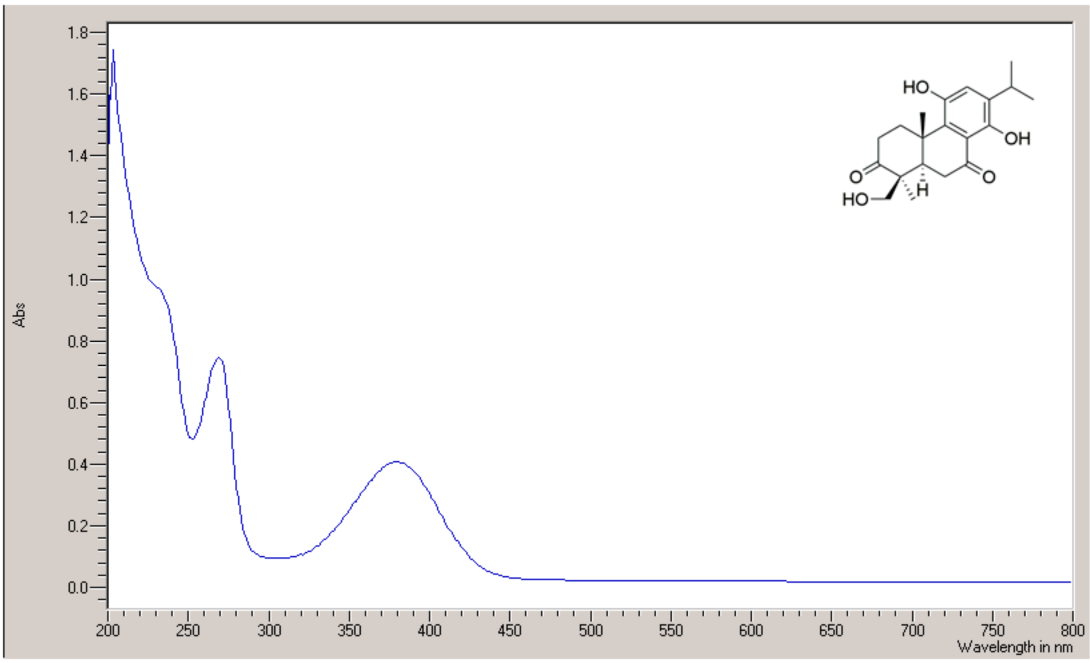


**Figure S73.** UV spectrum of compound **8** in MeOH.

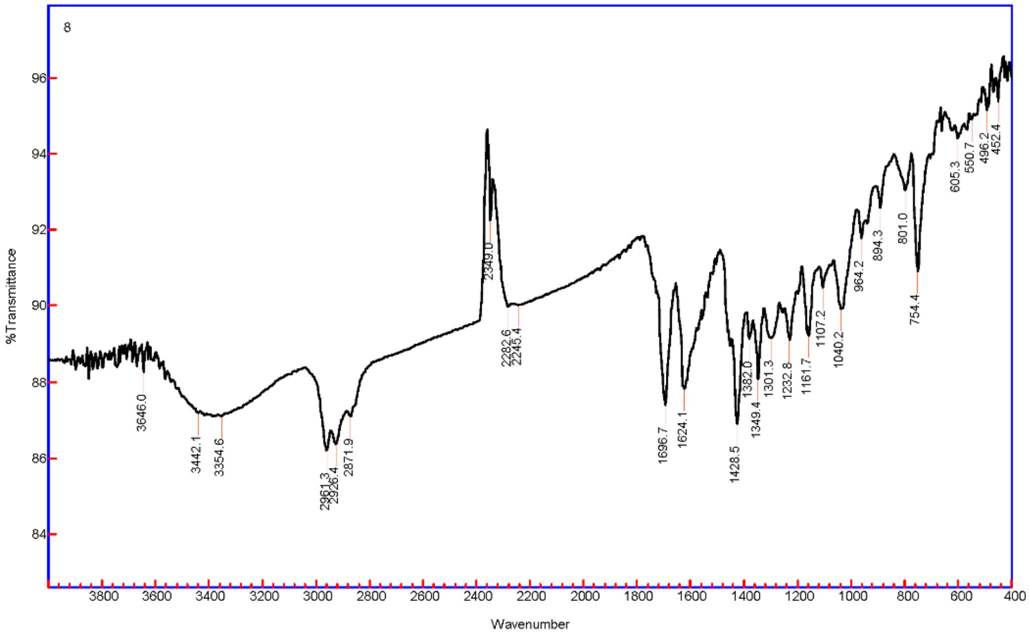


**Figure S74.** IR spectrum of compound **8**.

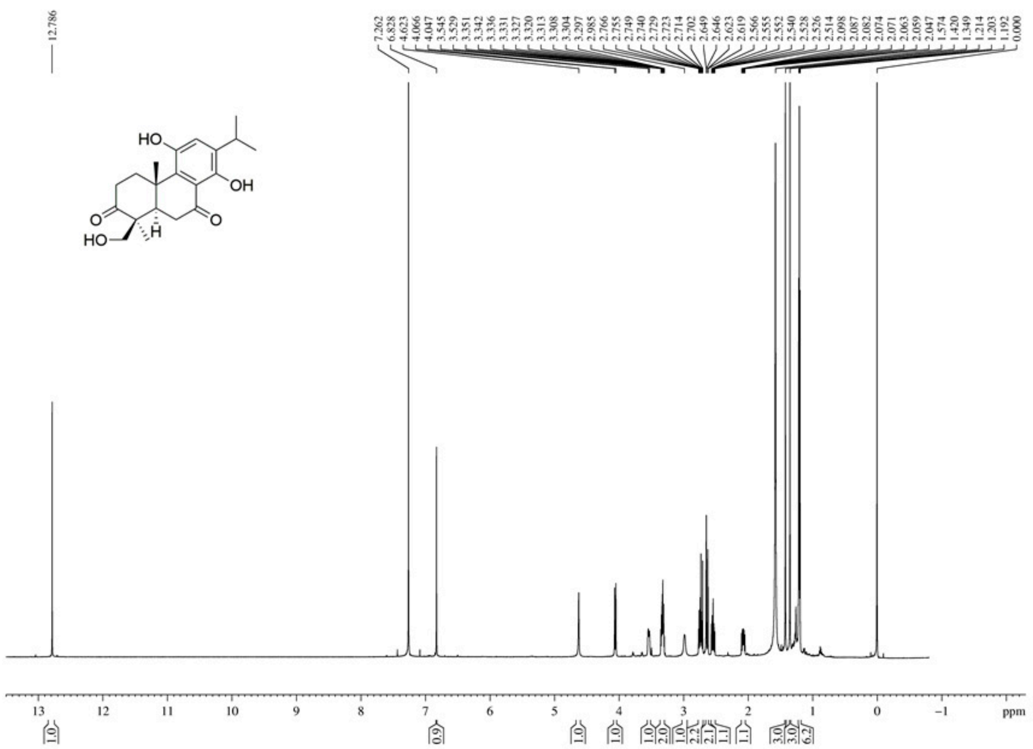

**Figure S75.** $^{1}$H NMR spectrum of compound **8** in $CDCl_3$.

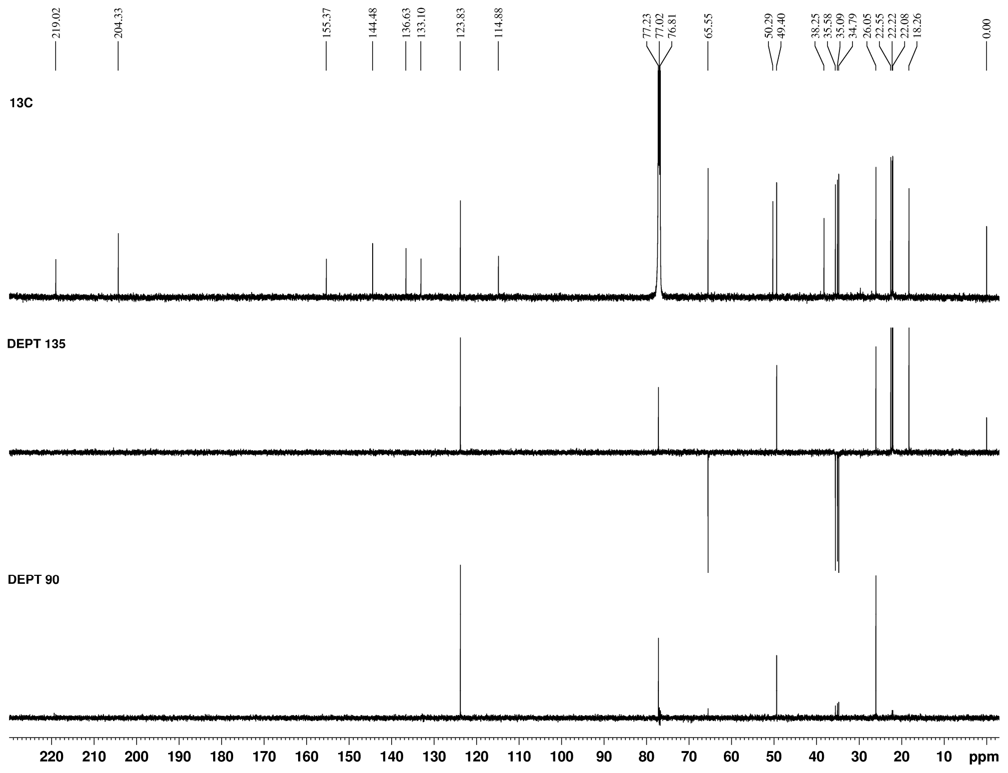

**Figure S76.** $^{13}$C and DEPT NMR spectra of compound **8** in $CDCl_3$.

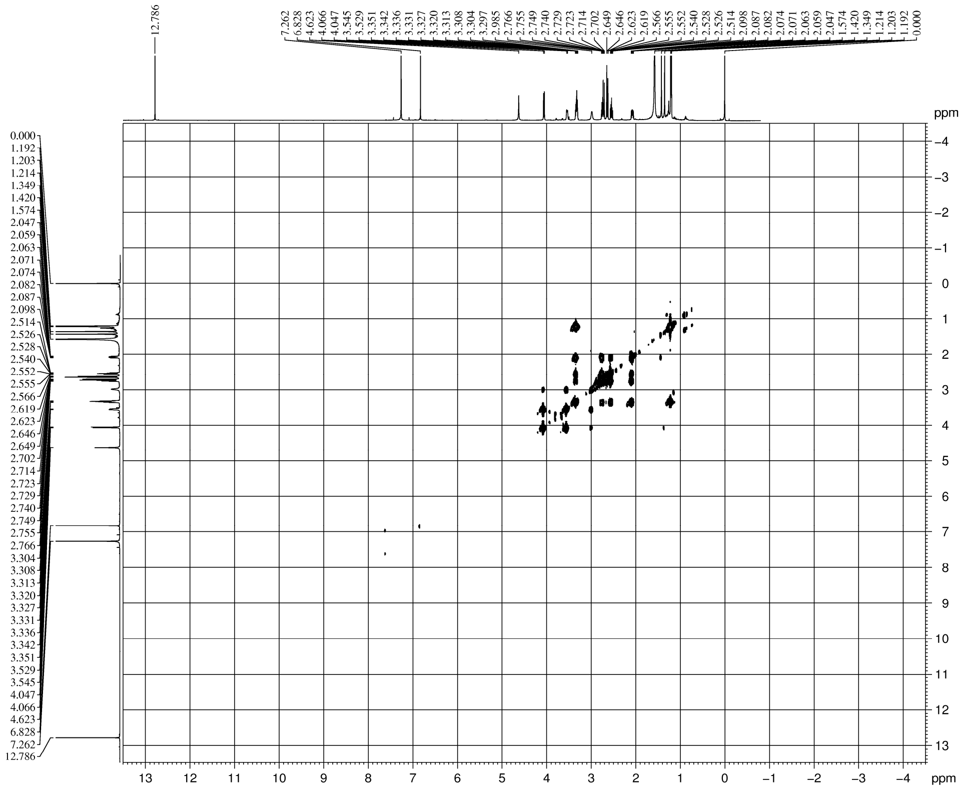

**Figure S77.** $^{1}$H–$^{1}$H COSY spectrum of compound **8** in CDCl3.

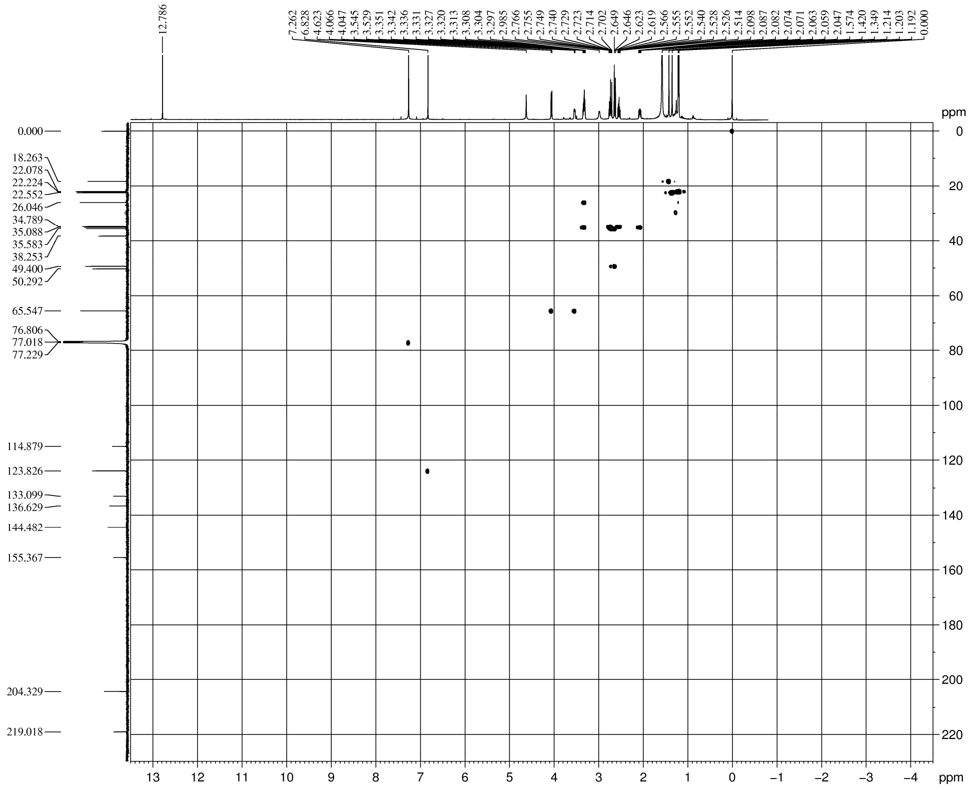

**Figure S78.** HSQC spectrum of compound **8** in CDCl3.

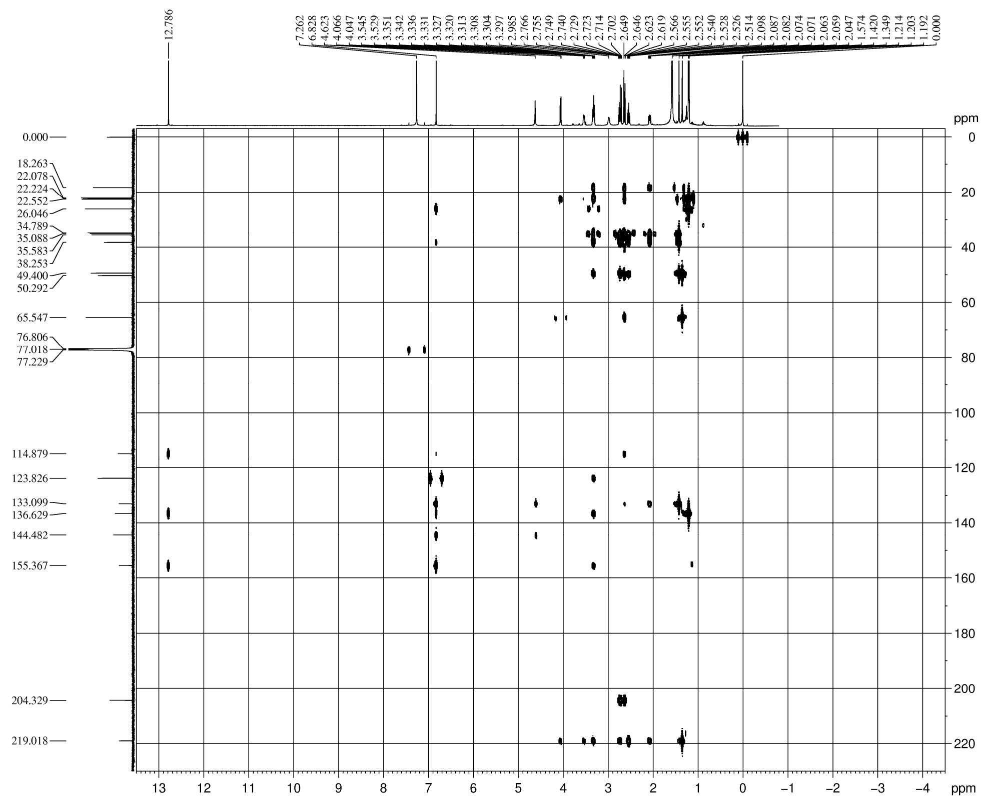

**Figure S79.** HMBC spectrum of compound **8** in $CDCl_3$.

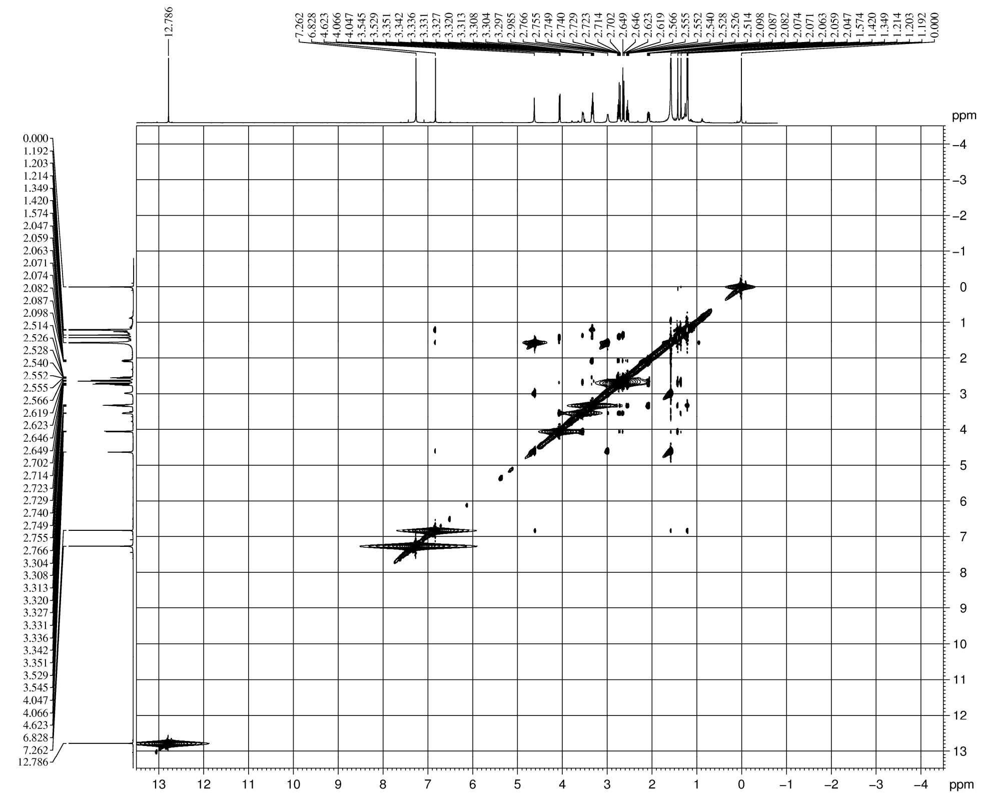

**Figure S80.** NOESY spectrum of compound **8** in $CDCl_3$.

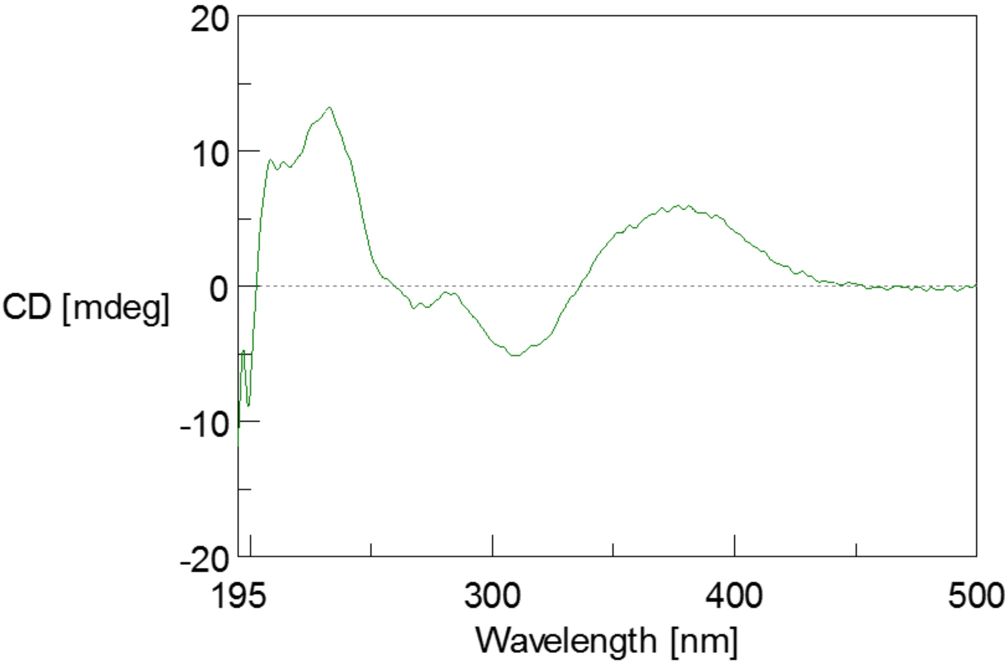


**Figure S81.** CD spectrum of compound **8** in MeOH.

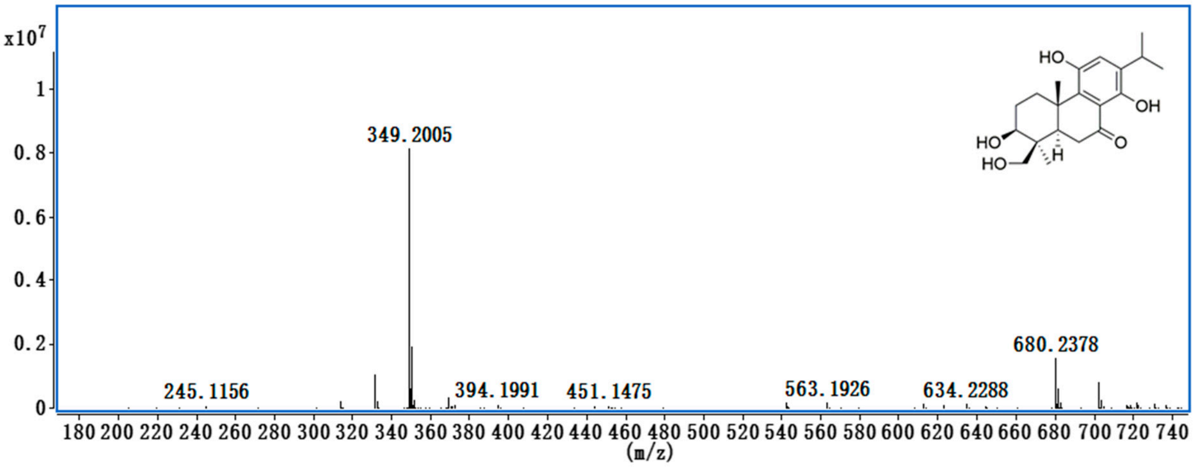


**Figure S82.** HRESIMS spectrum of compound **9**.

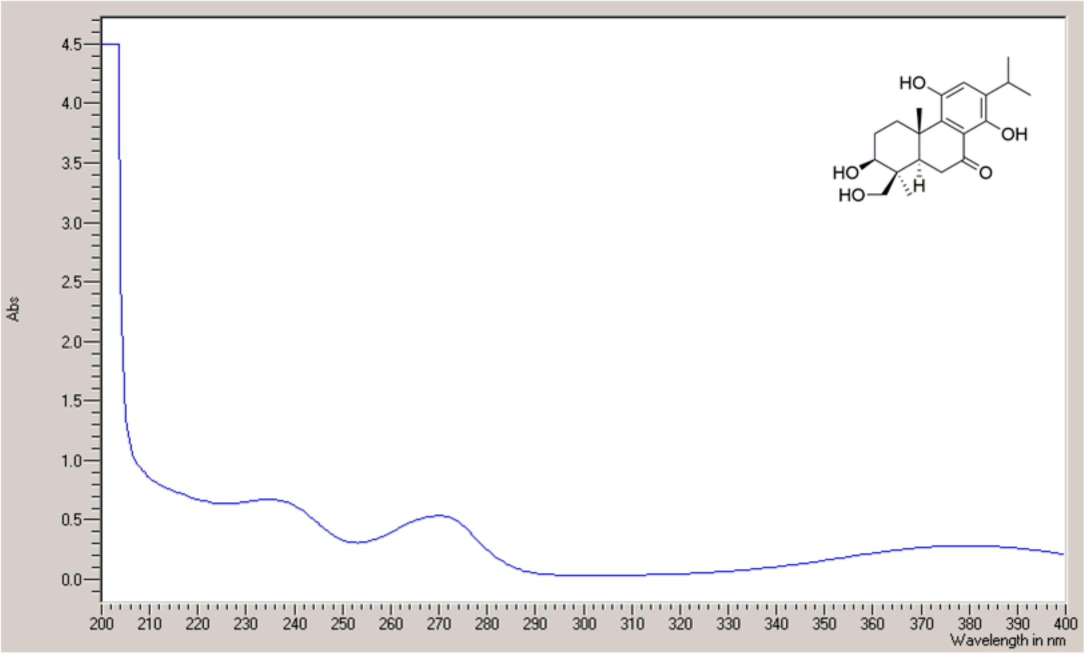


**Figure S83.** UV spectrum of compound **9** in MeOH.

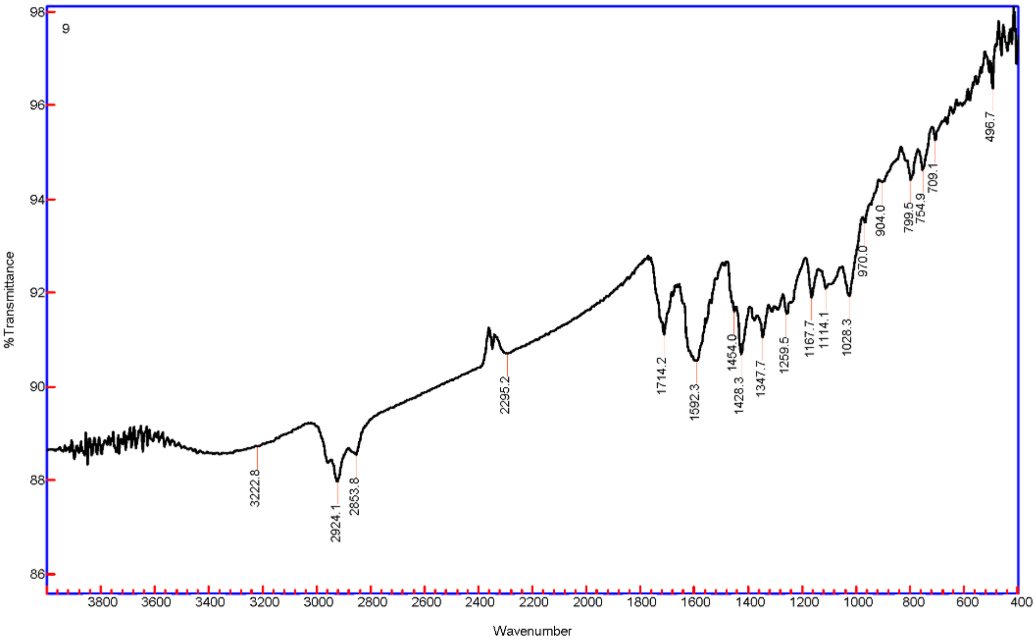


**Figure S84.** IR spectrum of compound **9**.

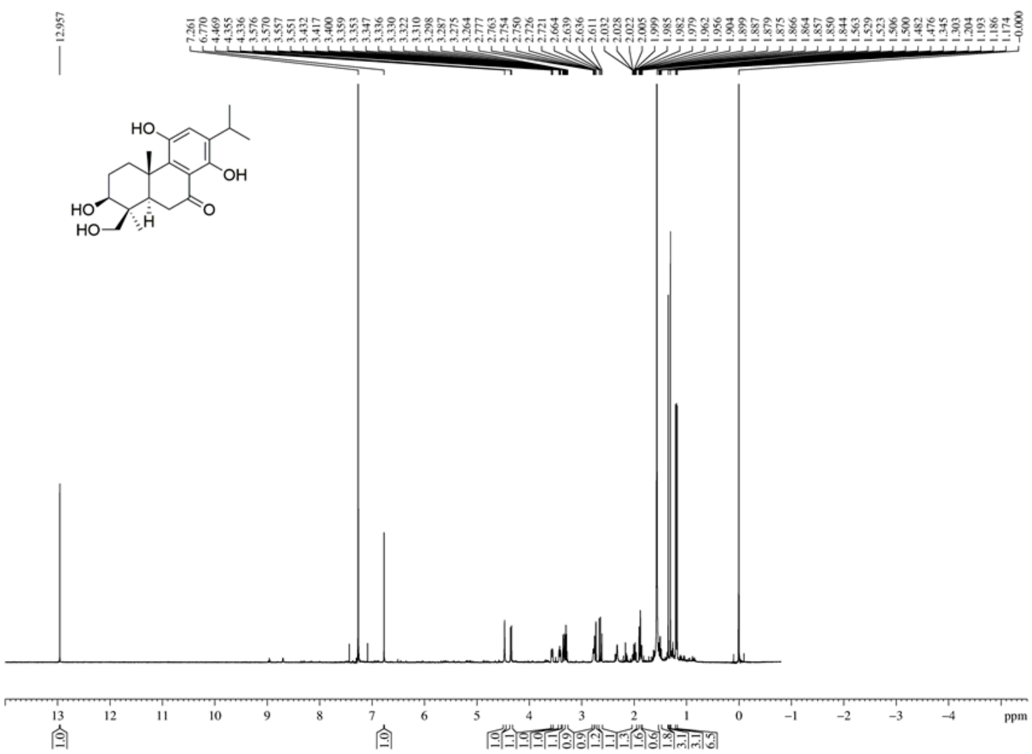

**Figure S85.** $^{1}H$ NMR spectrum of compound **9** in $CDCl_3$.

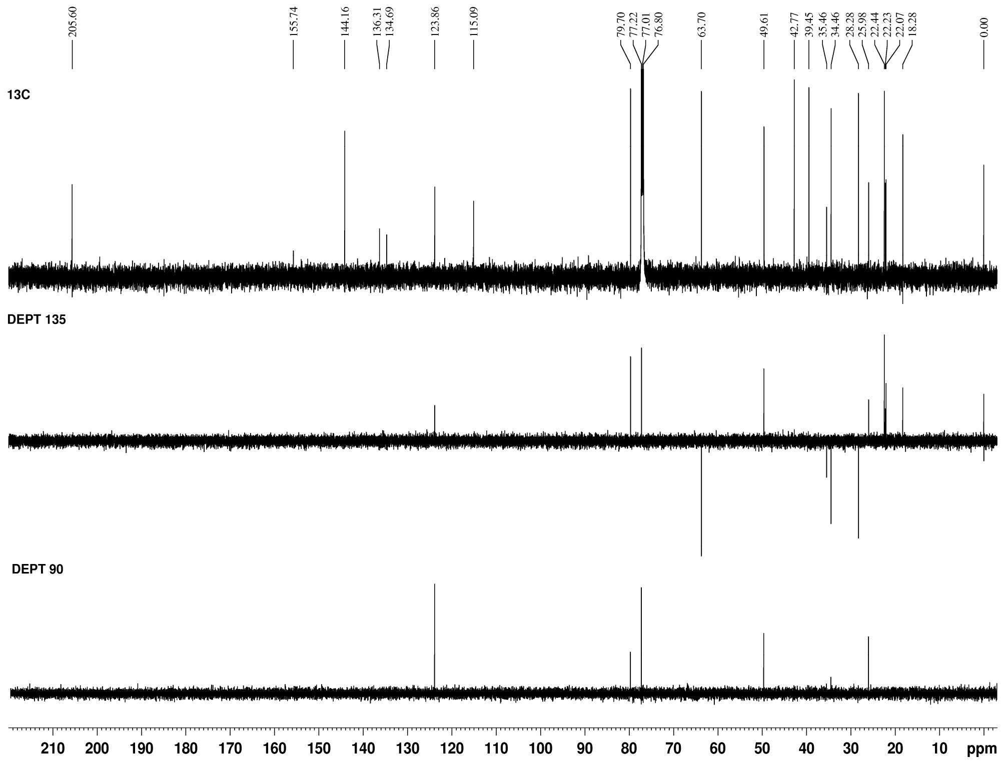

**Figure S86.** $^{13}C$ and DEPT NMR spectra of compound **9** in $CDCl_3$.

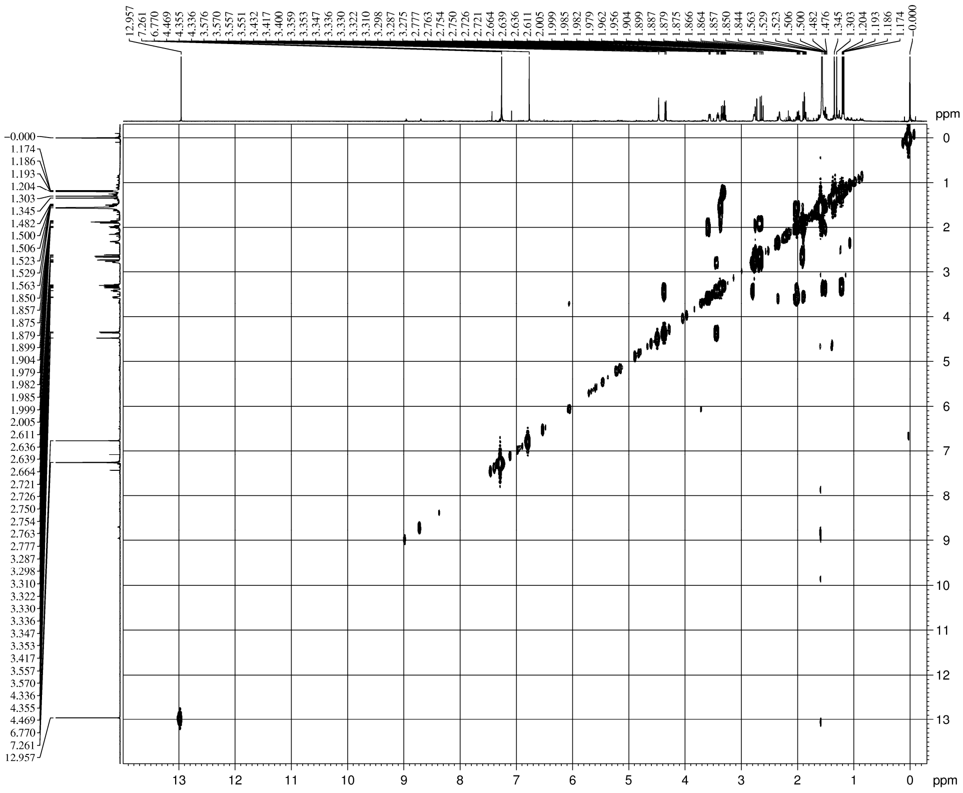

**Figure S87.** $^1H$–$^1H$ COSY spectrum of compound **9** in $CDCl_3$.

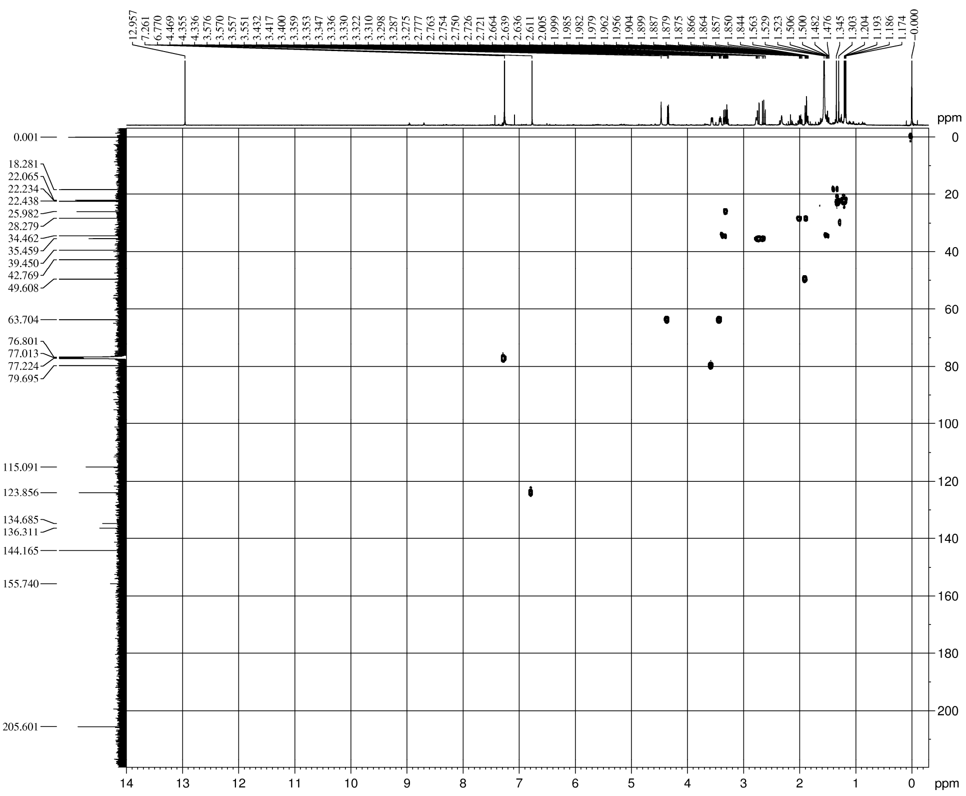

**Figure S88.** HSQC spectrum of compound **9** in $CDCl_3$.

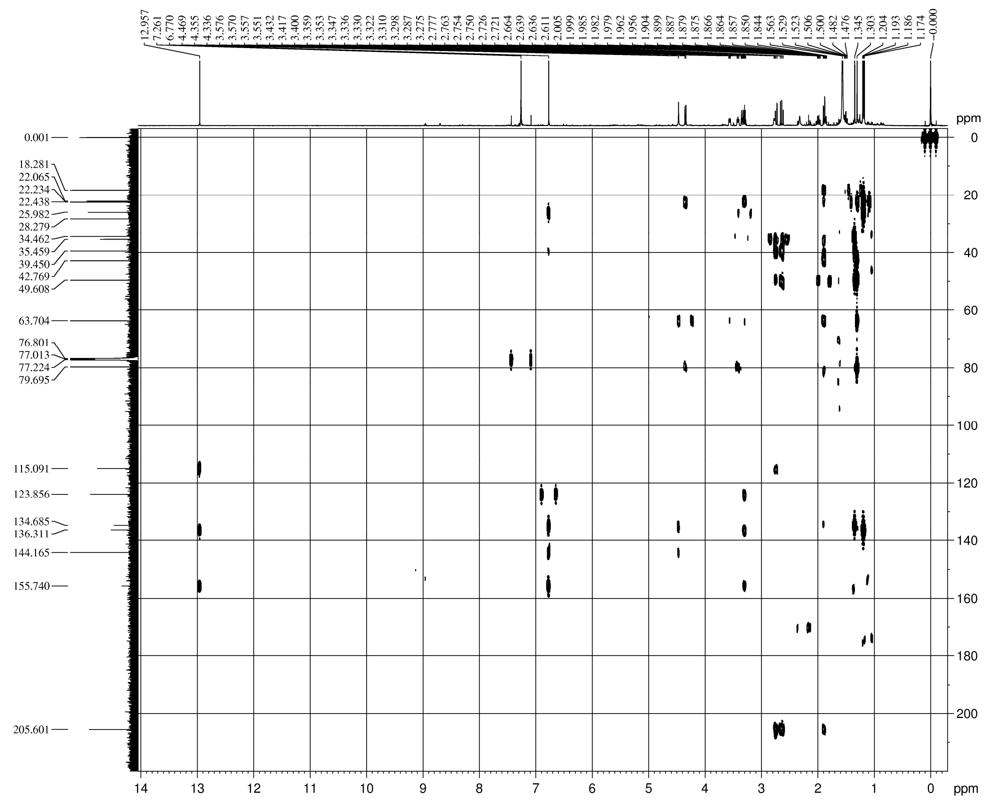


**Figure S89.** HMBC spectrum of compound **9** in CDCl3.

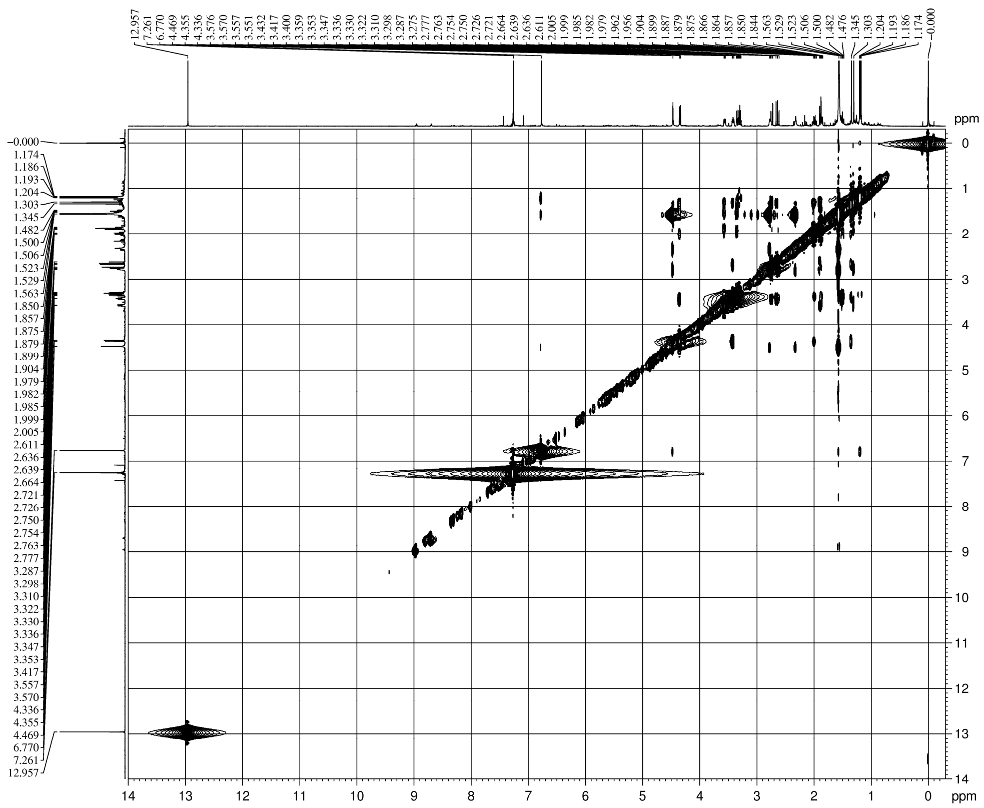


**Figure S90.** NOESY spectrum of compound **9** in CDCl3.

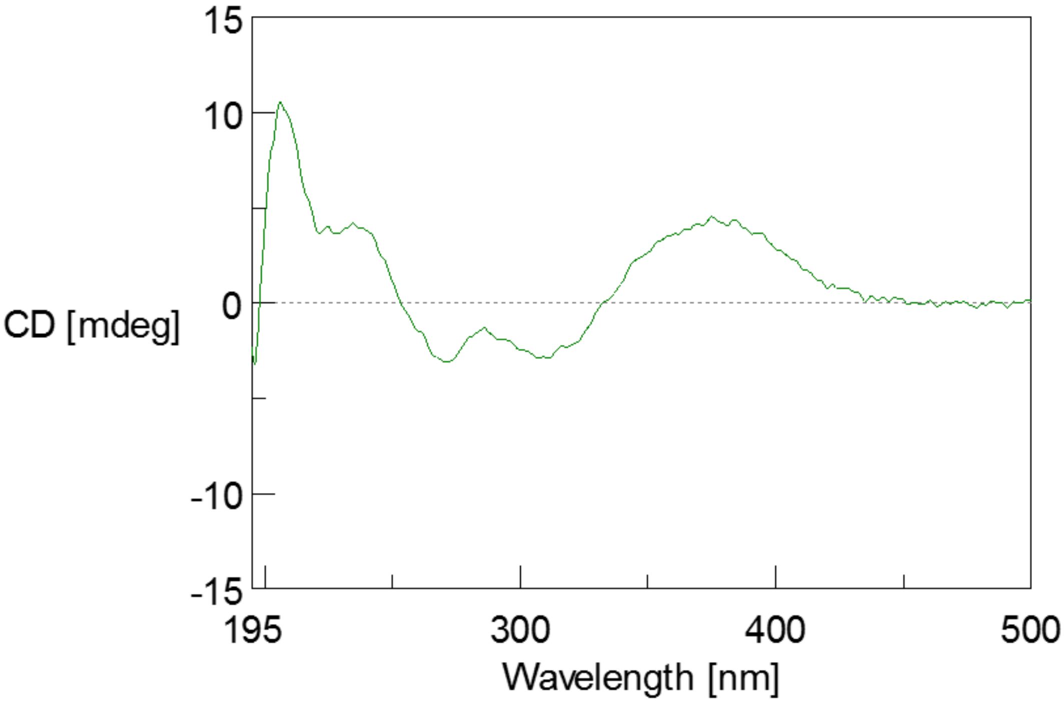


**Figure S91.** CD spectrum of compound **9** in MeOH.

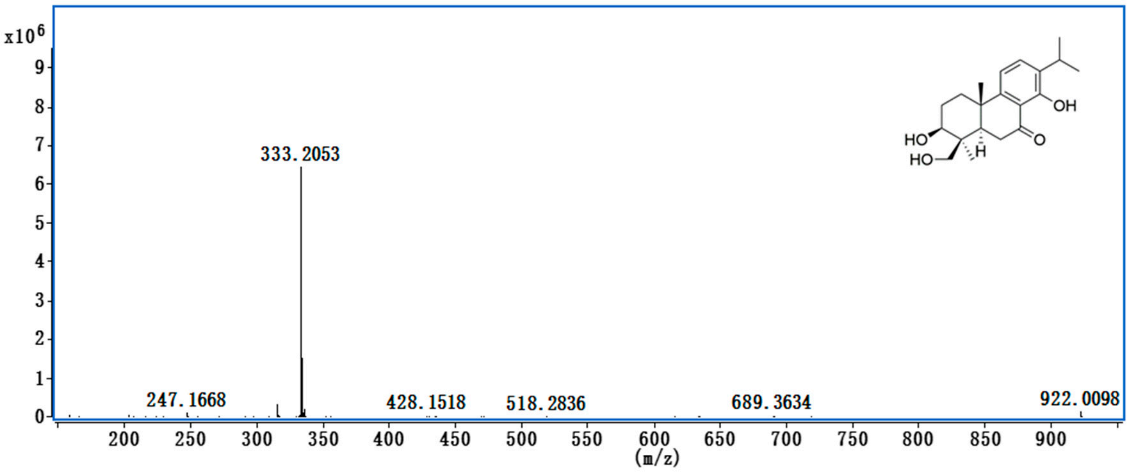


**Figure S92.** HRESIMS spectrum of compound **10**.

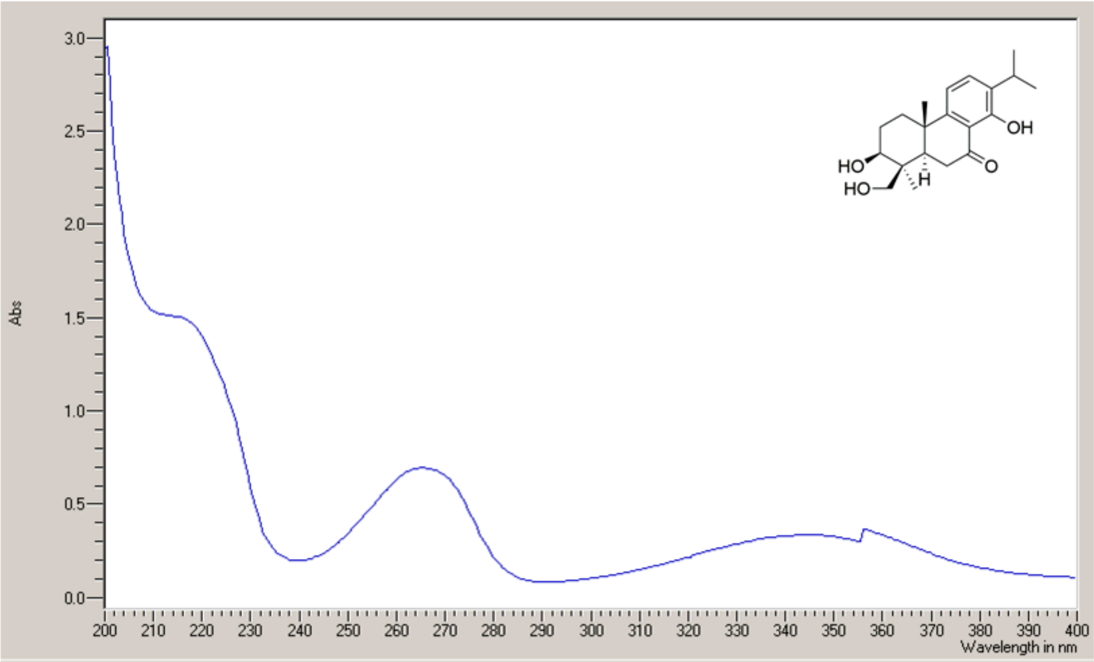


**Figure S93.** UV spectrum of compound **10** in MeOH.

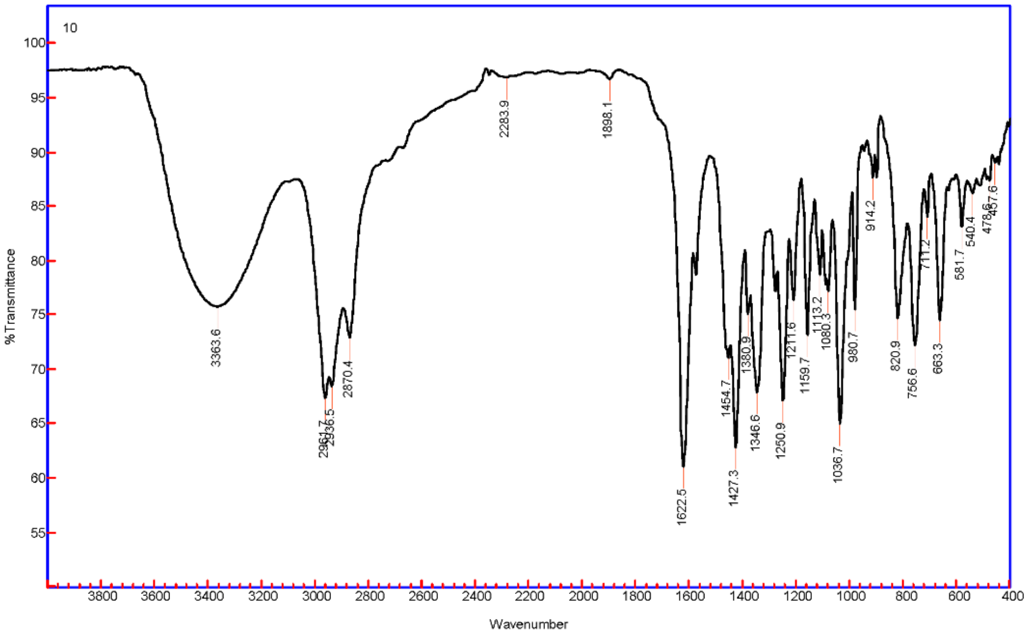


**Figure S94.** IR spectrum of compound **10**.

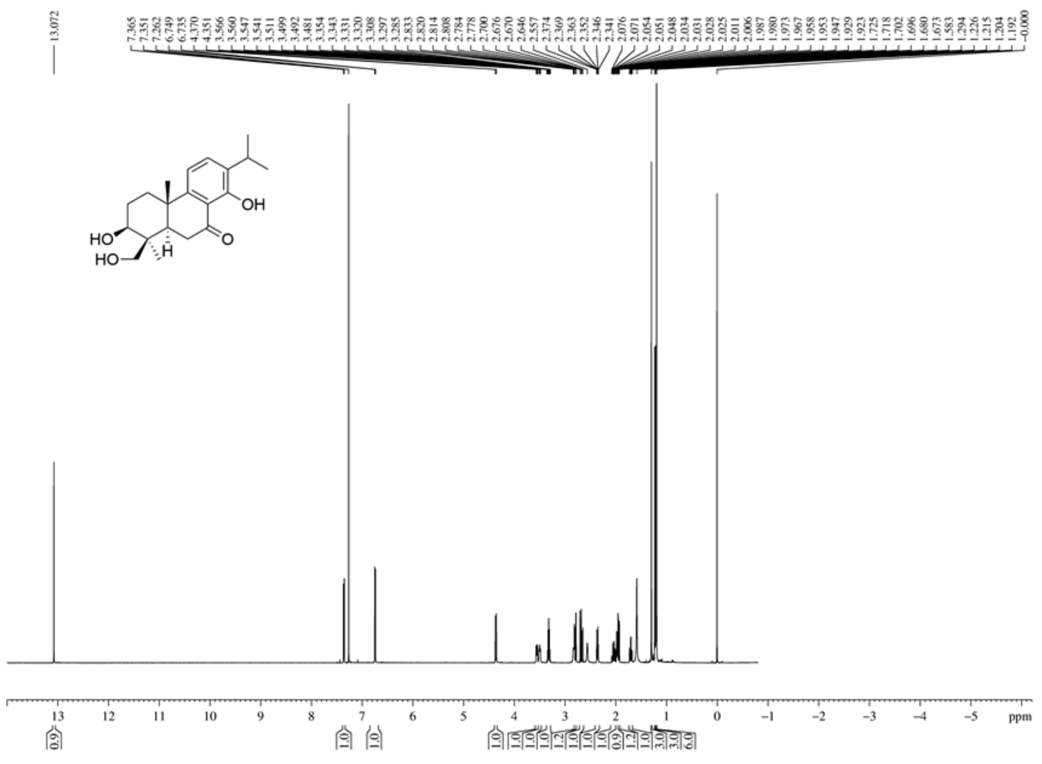

**Figure S95.** $^{1}H$ NMR spectrum of compound **10** in $CDCl_3$.

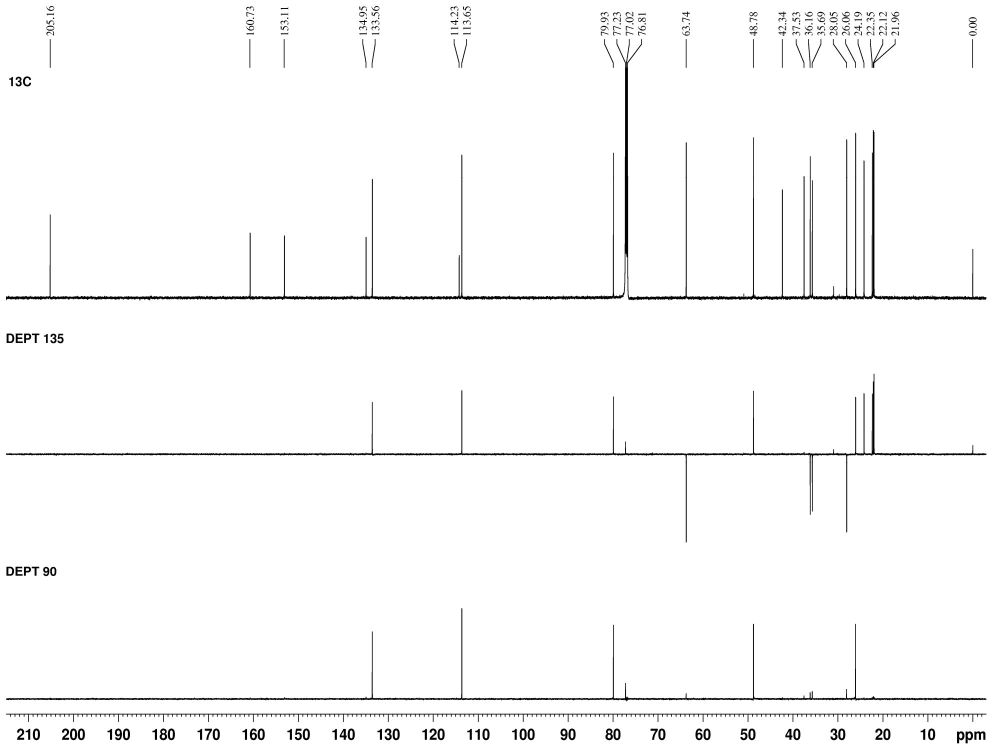


**Figure S96.** $^{13}C$ and DEPT NMR spectra of compound **10** in $CDCl_3$.

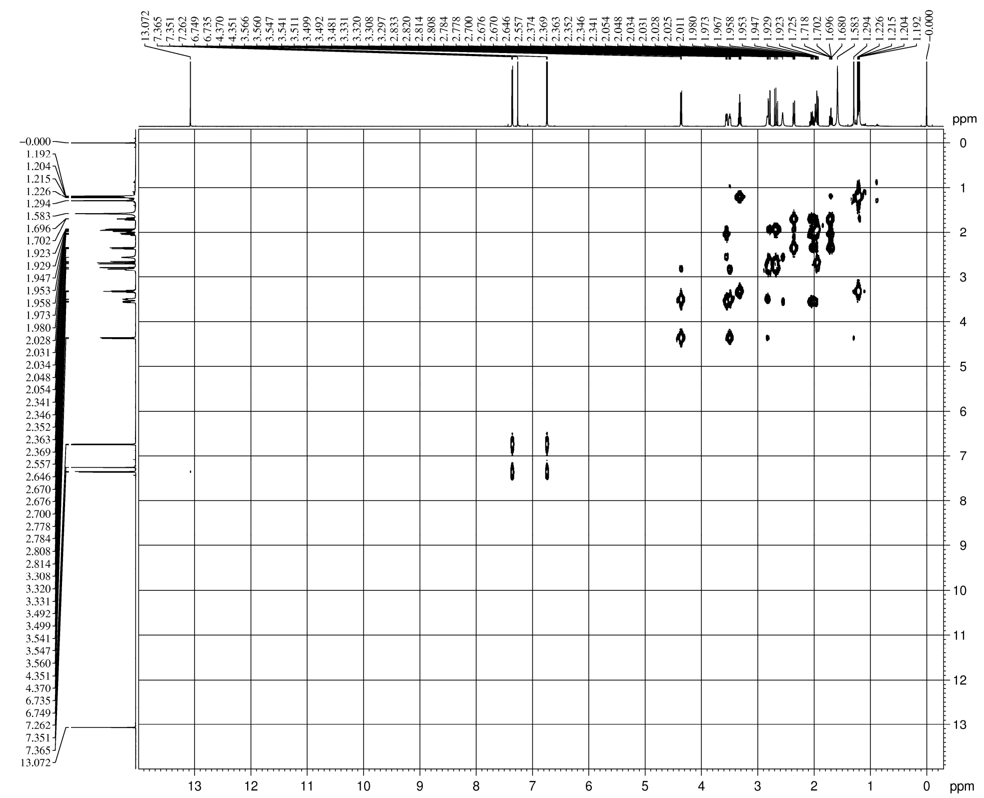

**Figure S97.** $^{1}$H–$^{1}$H COSY spectrum of compound **10** in $CDCl_3$.

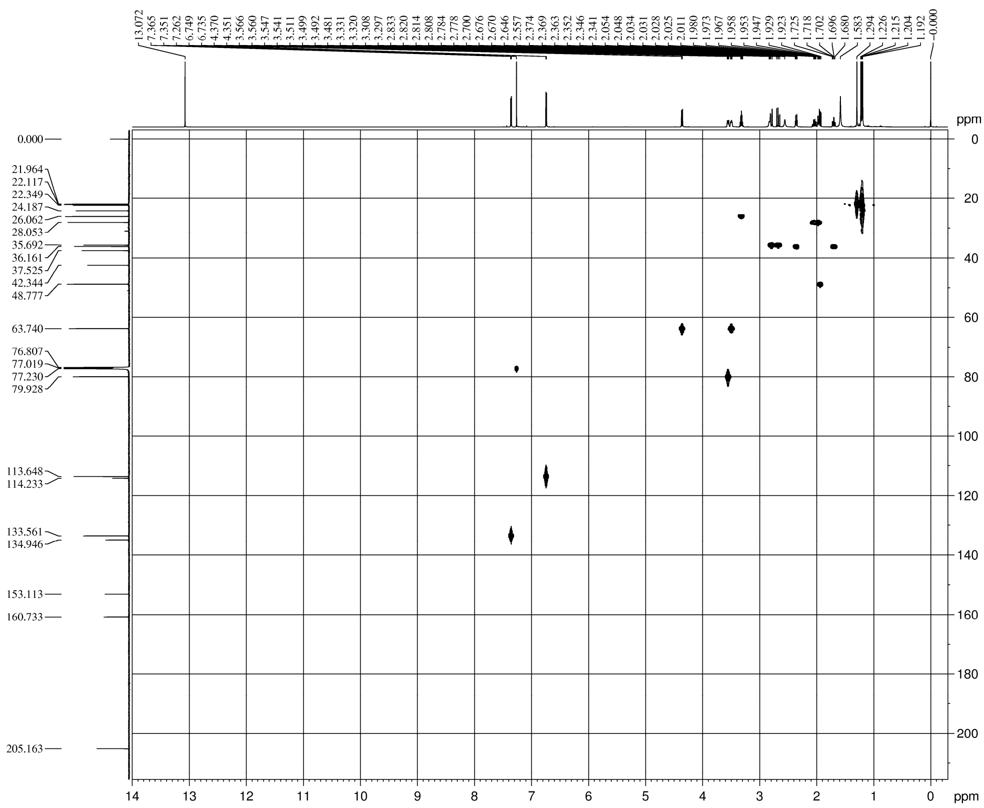

**Figure S98.** HSQC spectrum of compound **10** in $CDCl_3$.

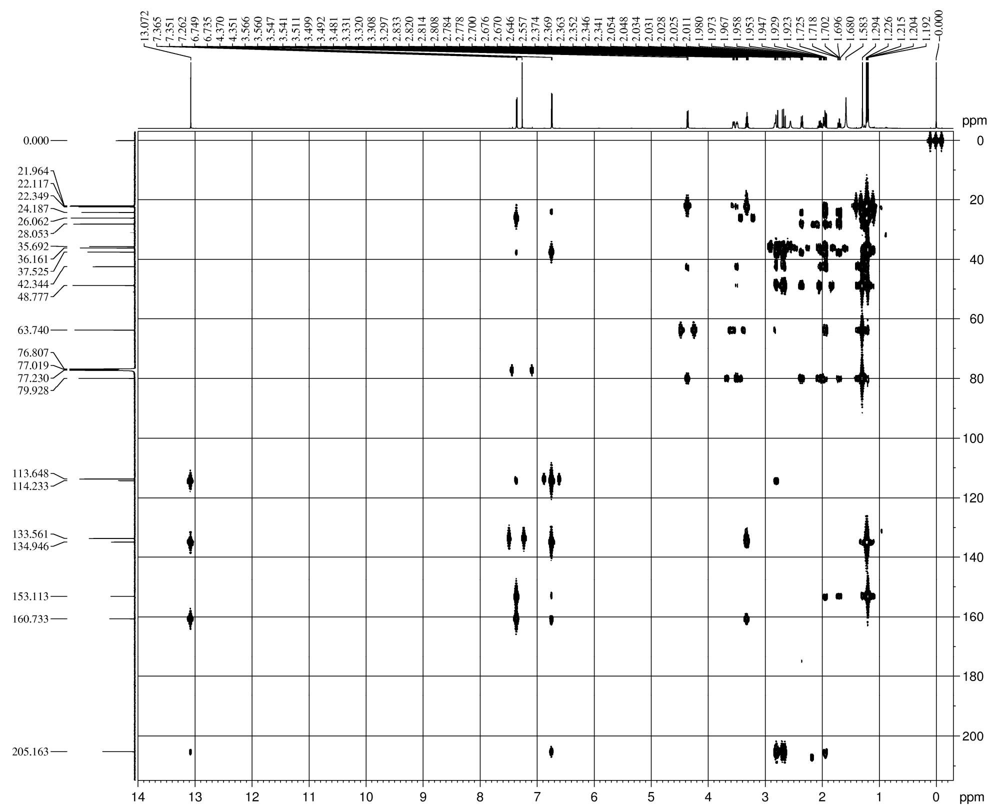

**Figure S99.** HMBC spectrum of compound **10** in $CDCl_3$.

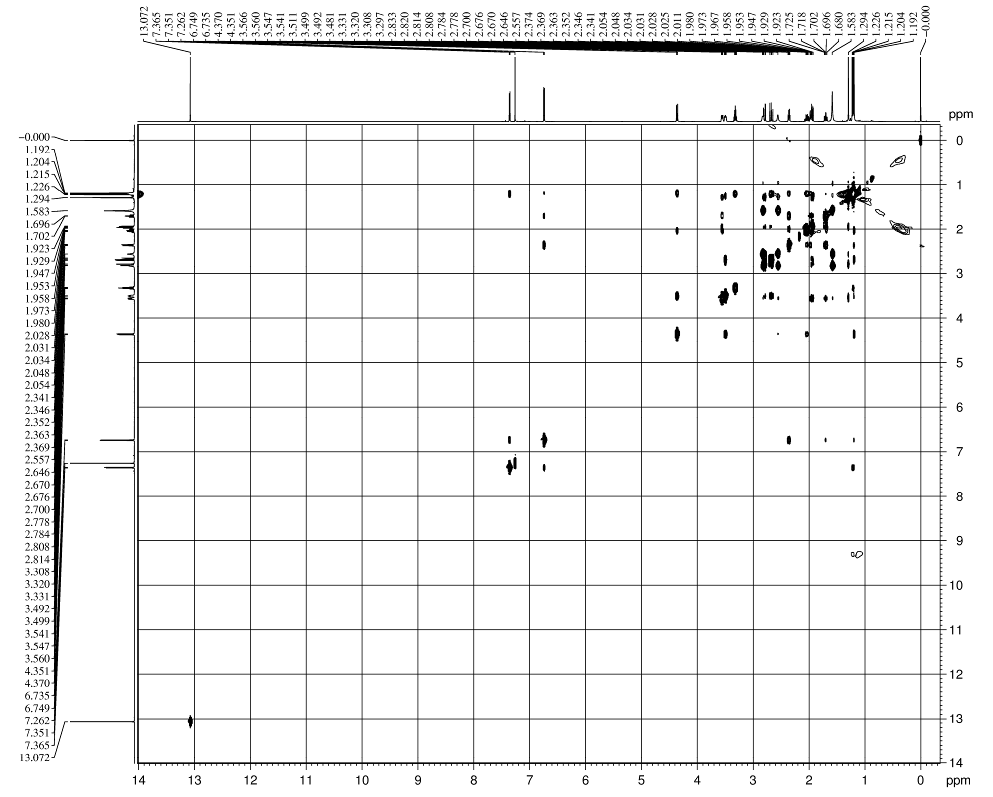

**Figure S100.** NOESY spectrum of compound **10** in $CDCl_3$.

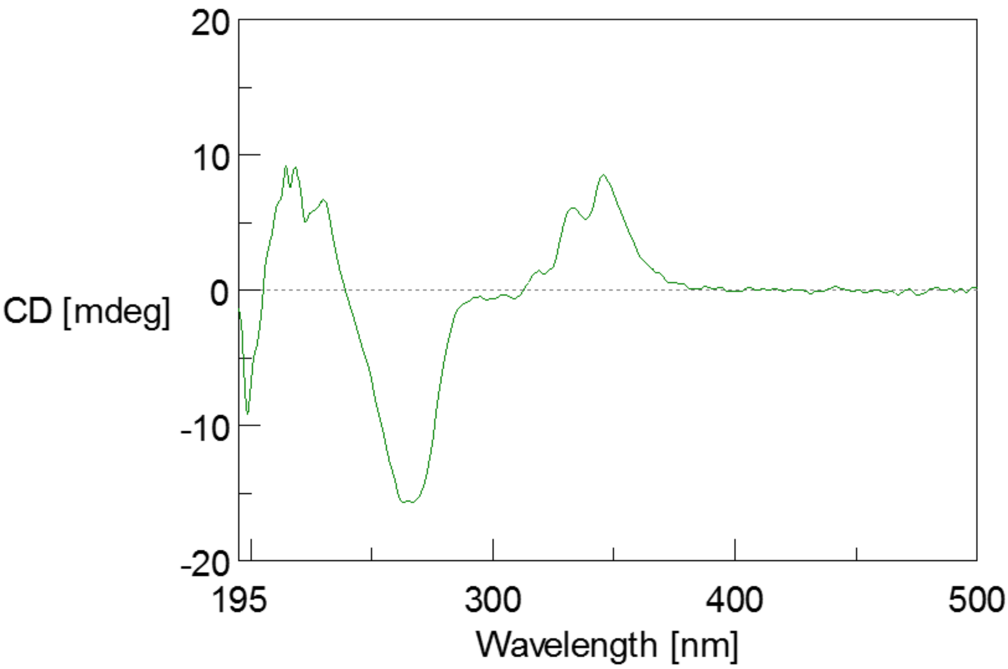


**Figure S101.** CD spectrum of compound **10** in MeOH.

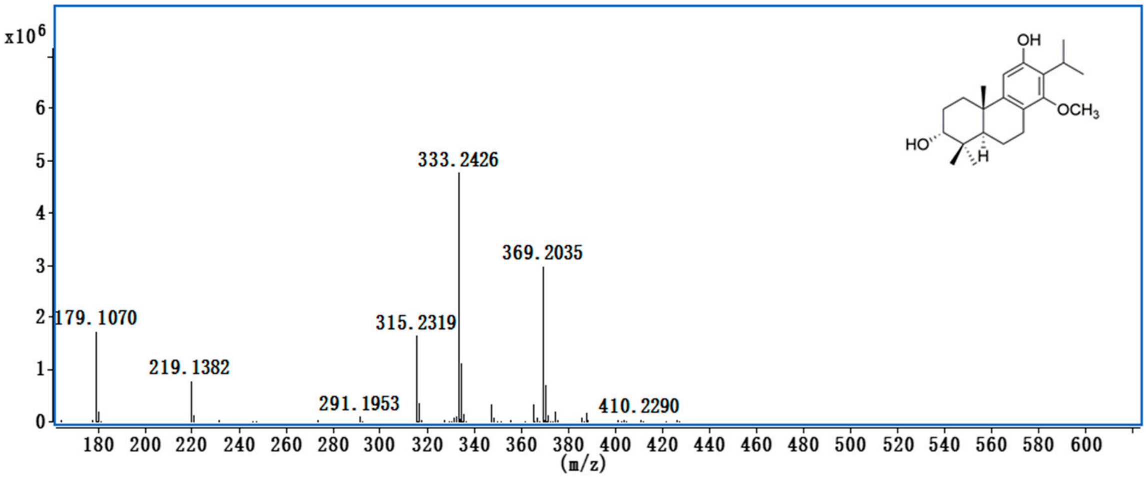


**Figure S102.** HRESIMS spectrum of compound **11**.

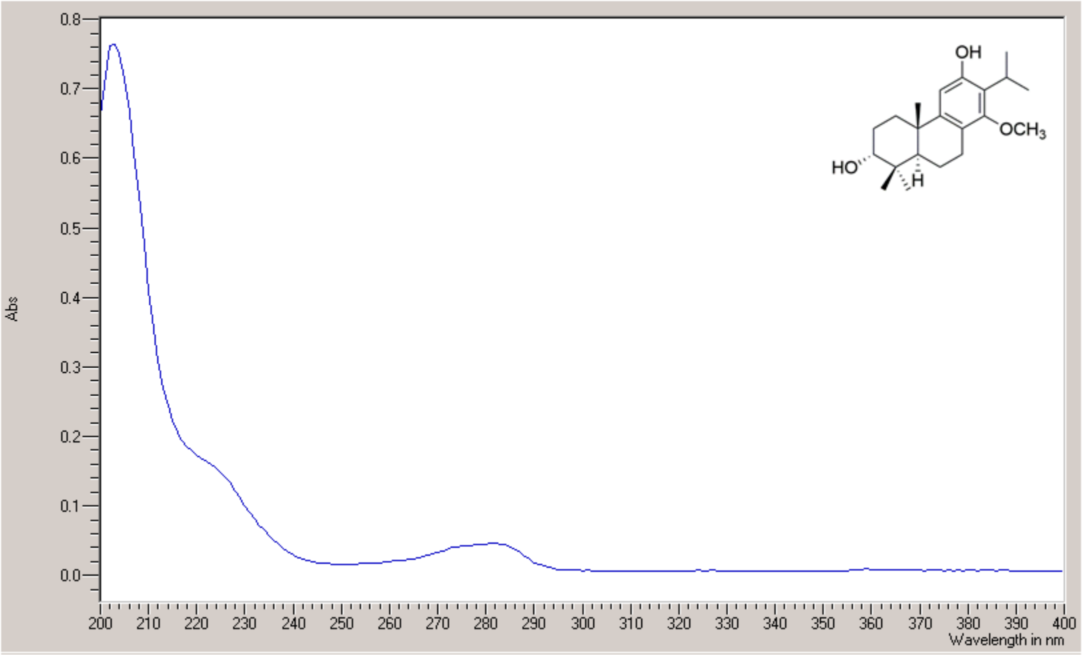


**Figure S103.** UV spectrum of compound **11** in MeOH.

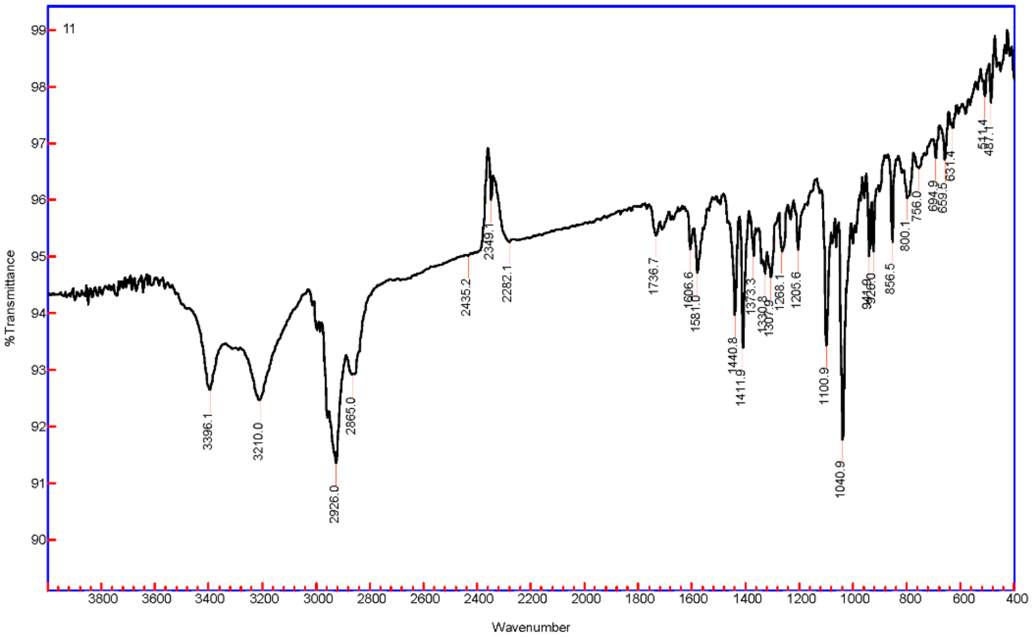


**Figure S104.** IR spectrum of compound **11**.

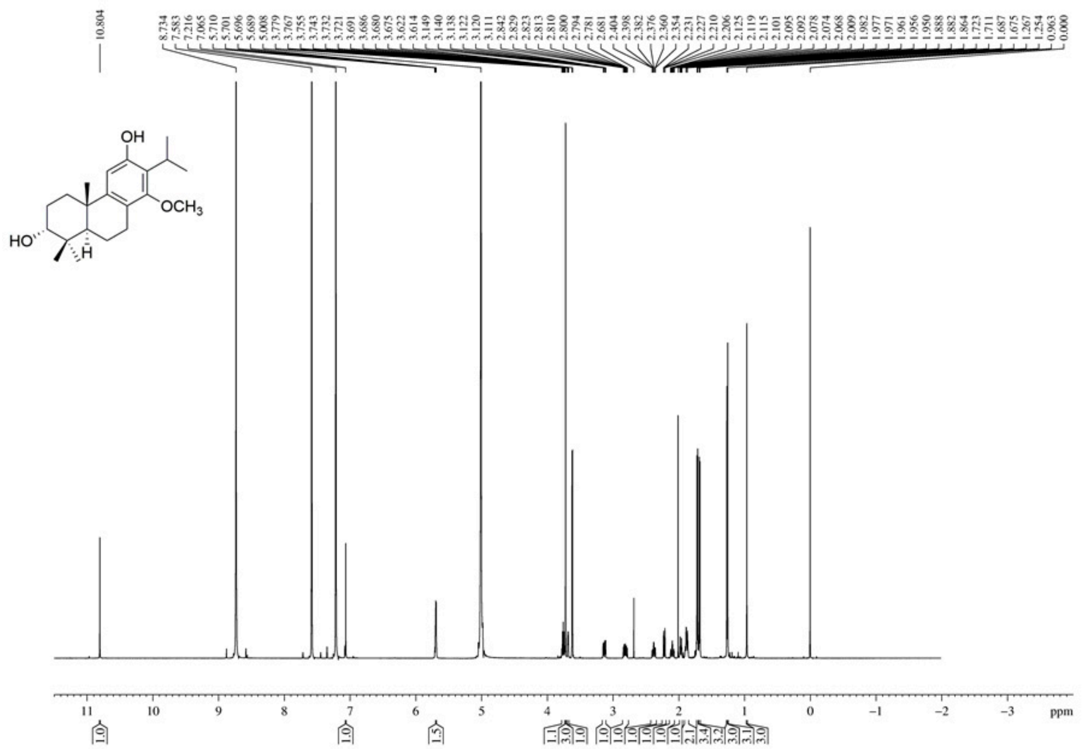

**Figure S105.** $^{1}$H NMR spectrum of compound **11** in pyridine-$d_5$.

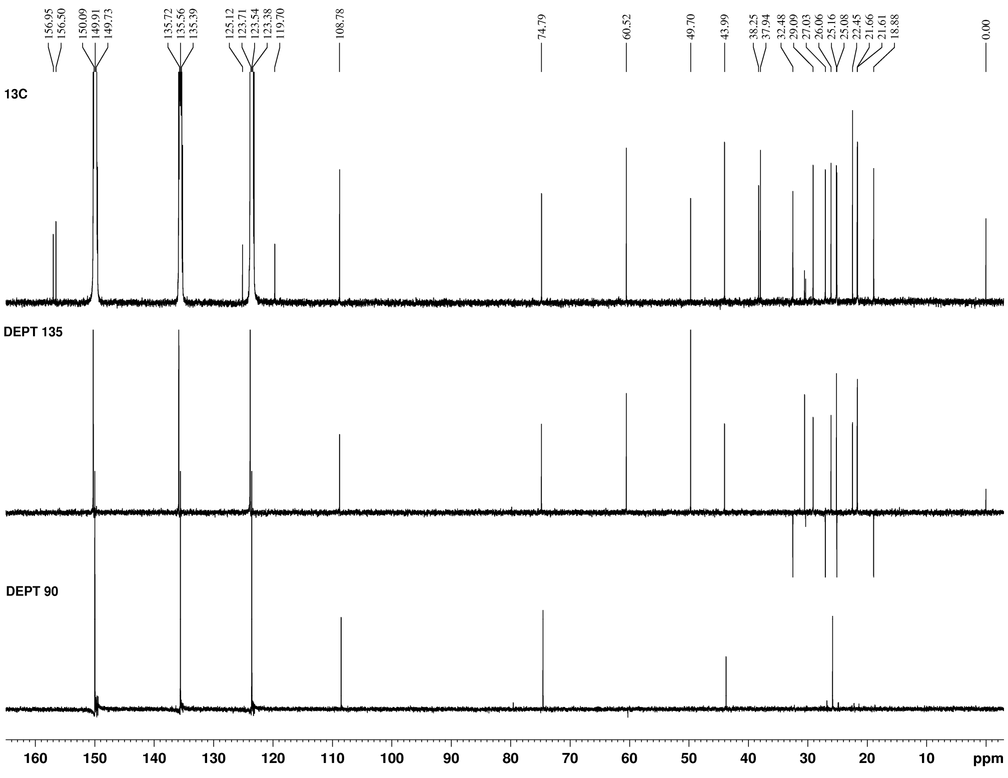


**Figure S106.** $^{13}$C and DEPT NMR spectra of compound **11** in pyridine-$d_5$.

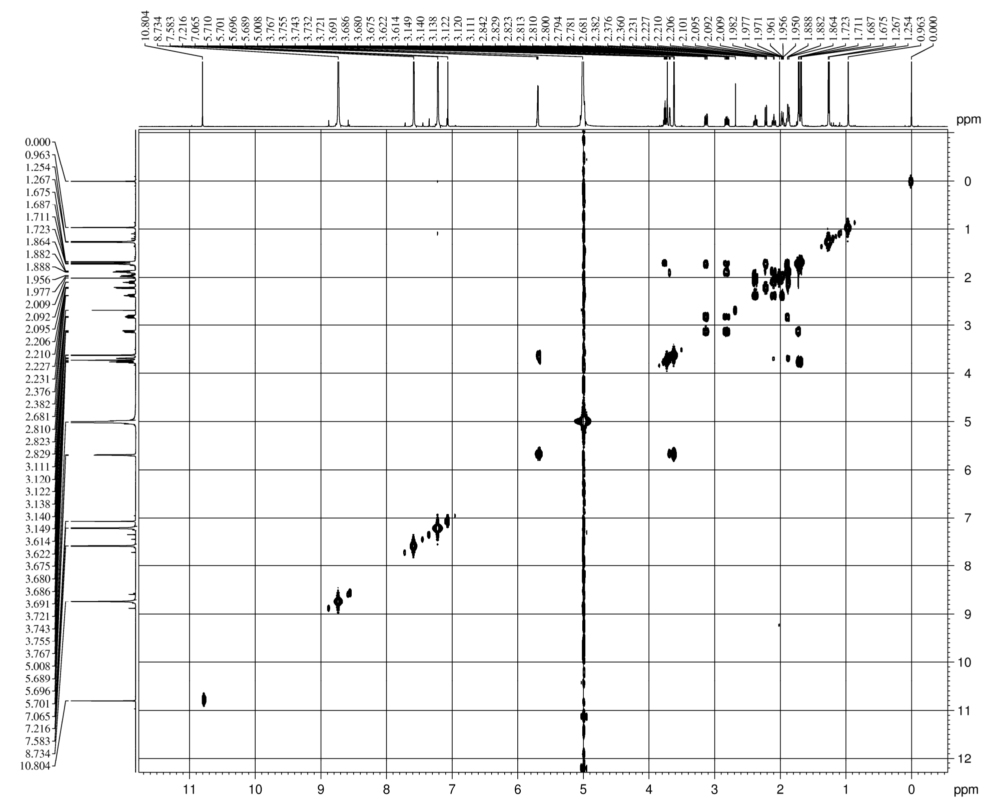

**Figure S107.** $^1H$–$^1H$ COSY spectrum of compound **11** in pyridine-$d_5$.

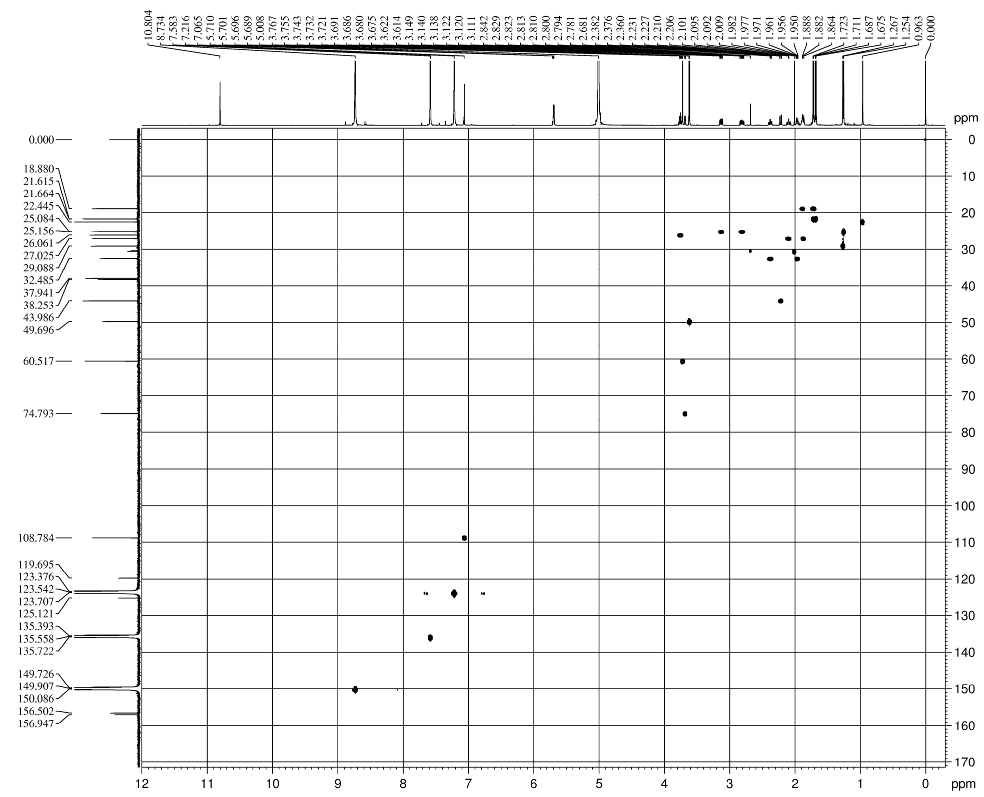

**Figure S108.** HSQC spectrum of compound **11** in pyridine-$d_5$.

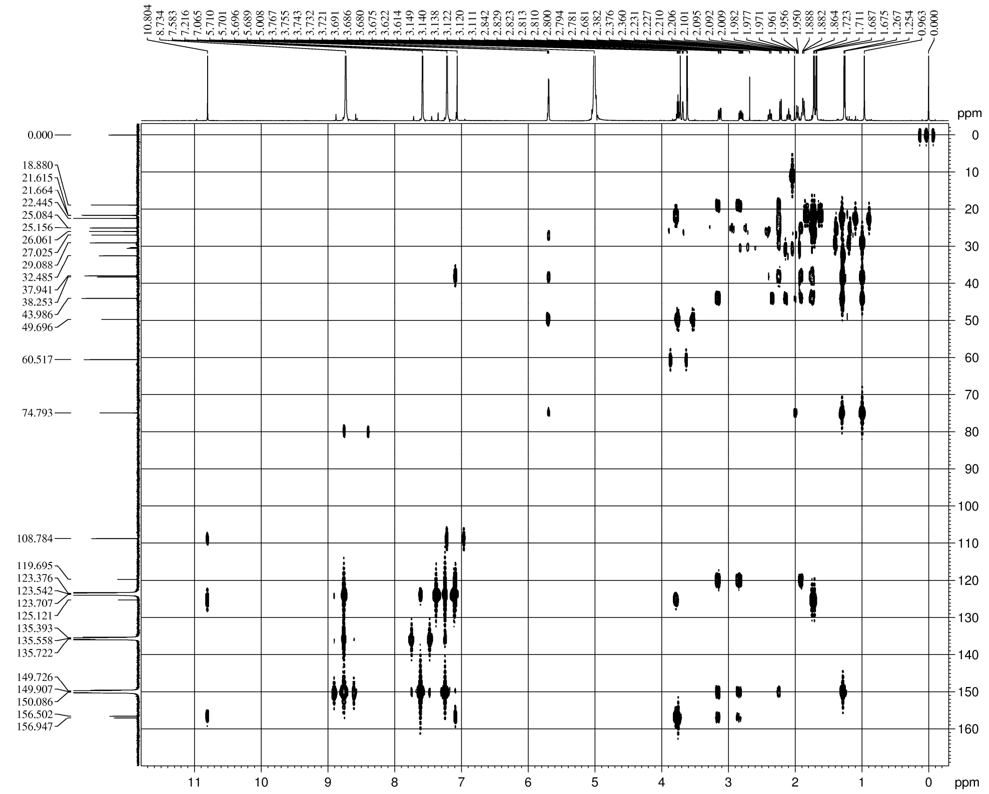

**Figure S109.** HMBC spectrum of compound **11** in pyridine-*d*5.

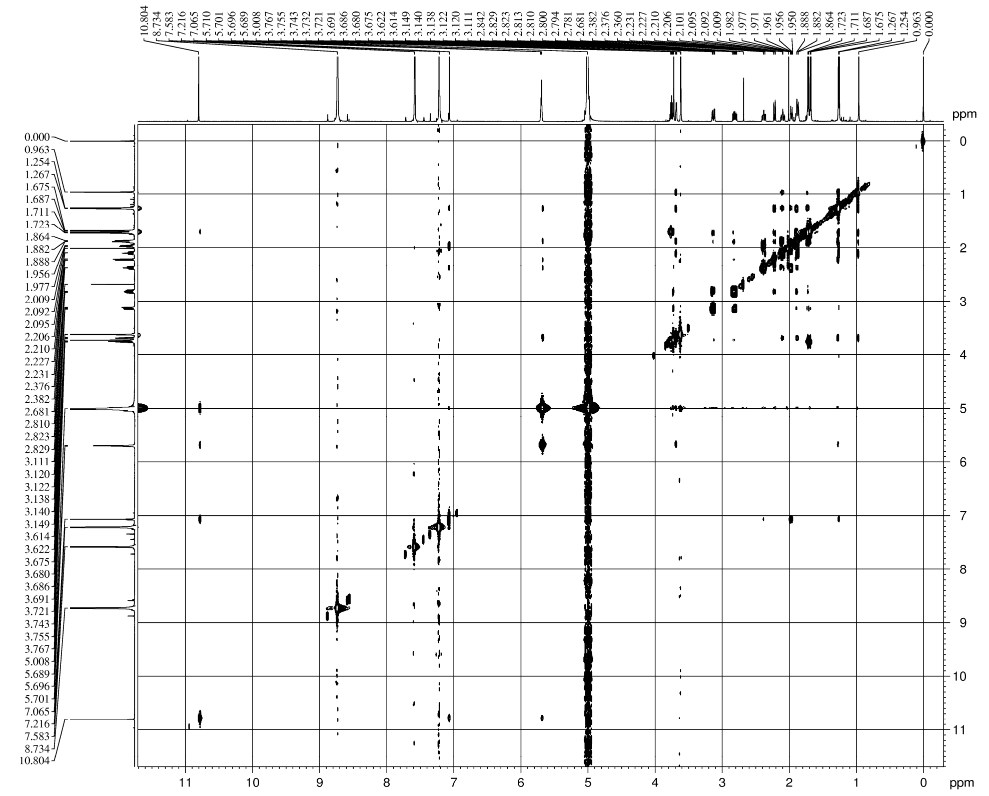

**Figure S110.** NOESY spectrum of compound **11** in pyridine-*d*5.

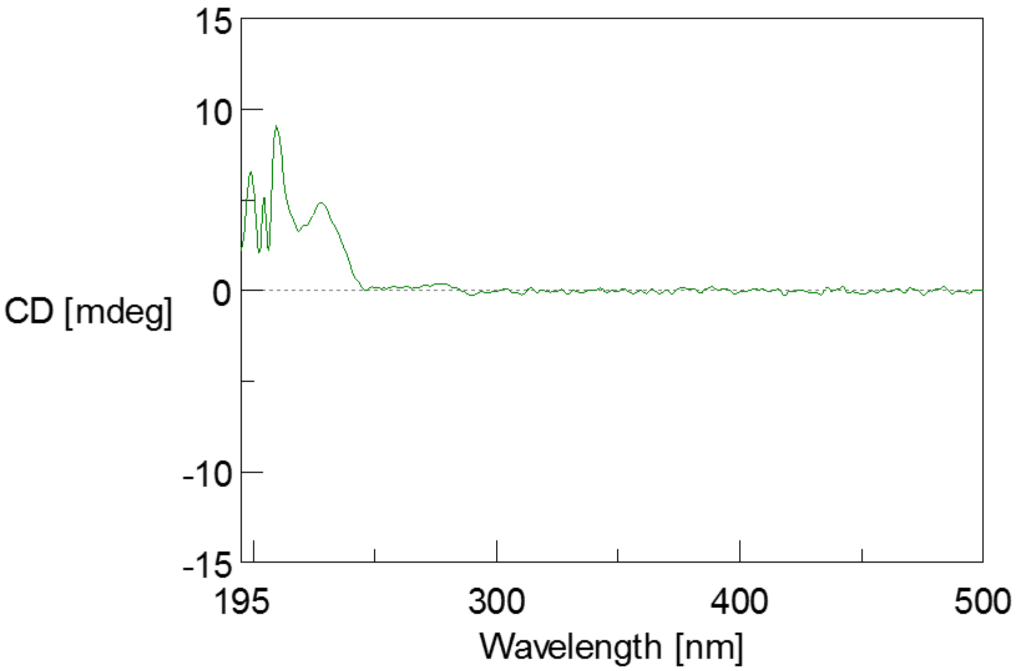


**Figure S111.** CD spectrum of compound **11** in MeOH.

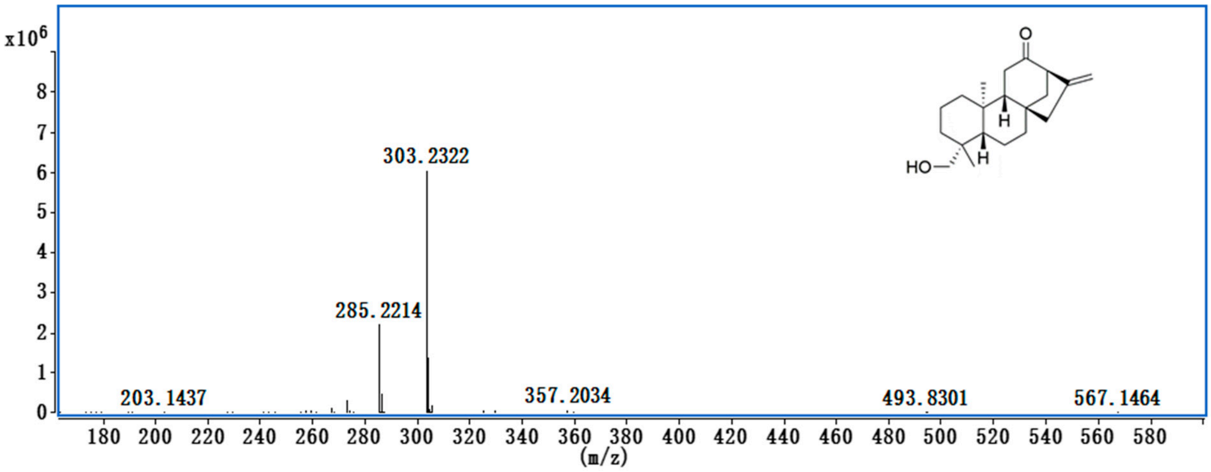


**Figure S112.** HRESIMS spectrum of compound **12**.

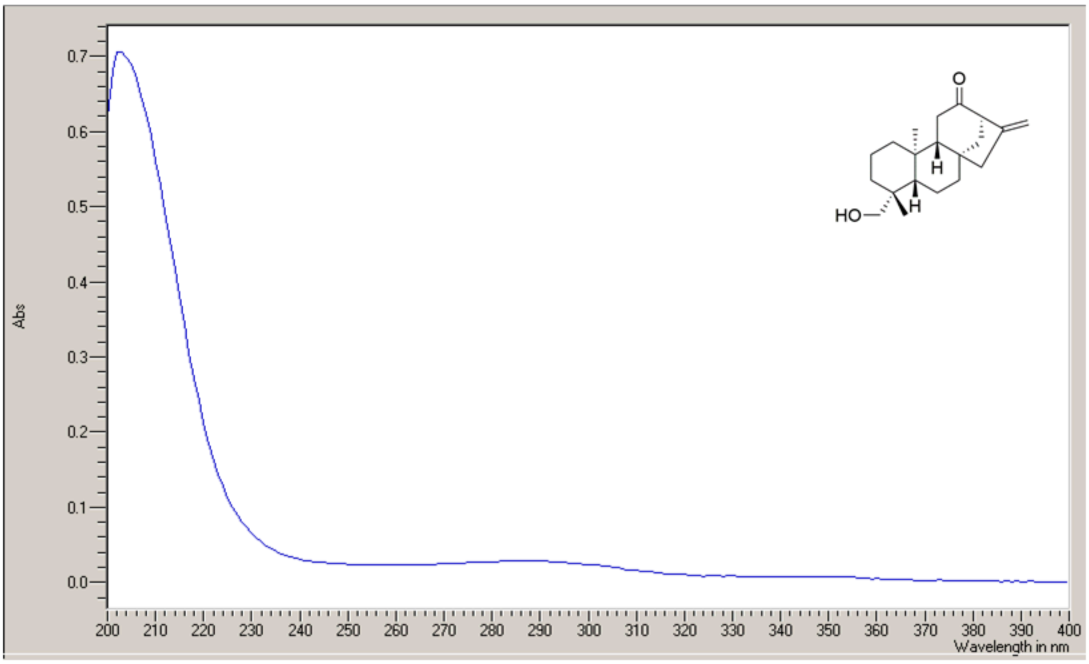


**Figure S113.** UV spectrum of compound **12** in MeOH.

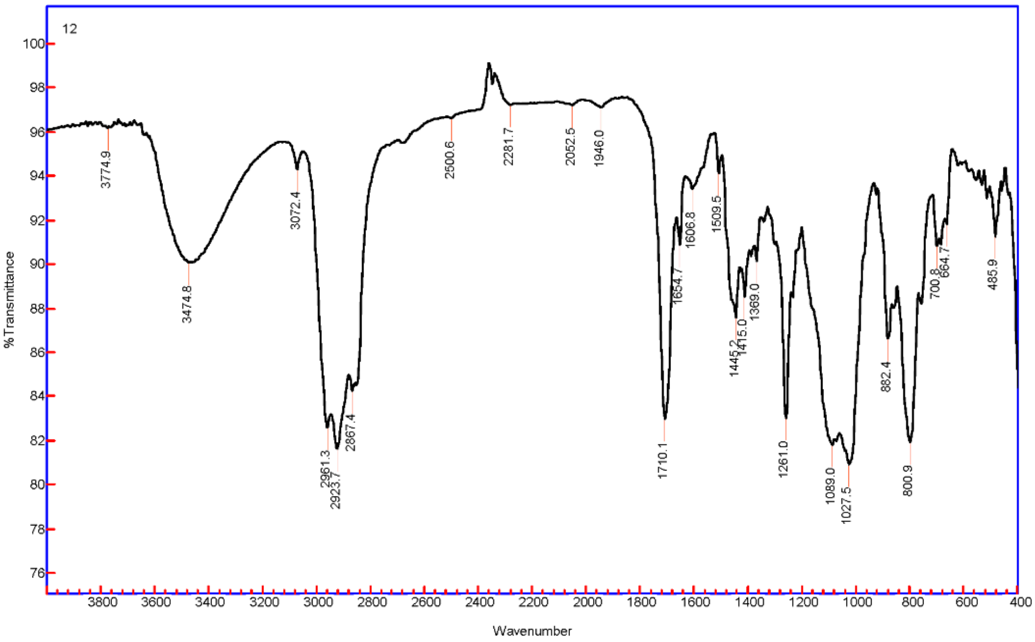


**Figure S114.** IR spectrum of compound **12**.

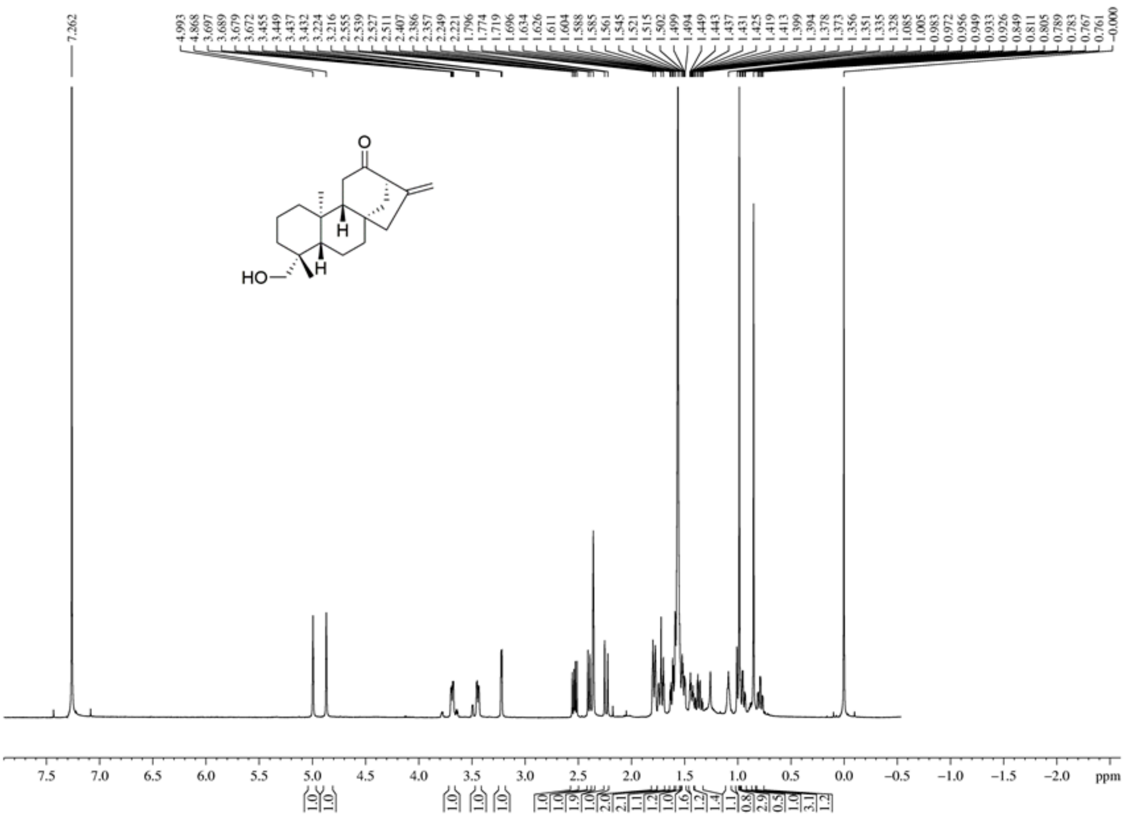


**Figure S115.** $^{1}$H NMR spectrum of compound **12** in $CDCl_3$.

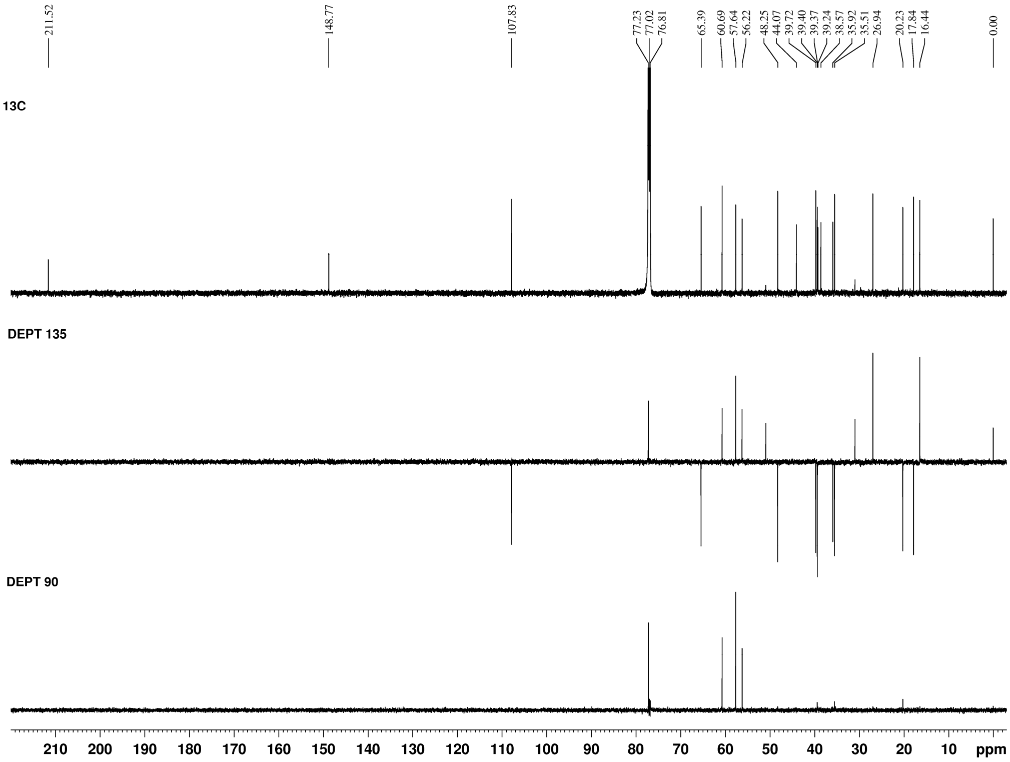


**Figure S116.** $^{13}$C and DEPT NMR spectra of compound **12** in $CDCl_3$.

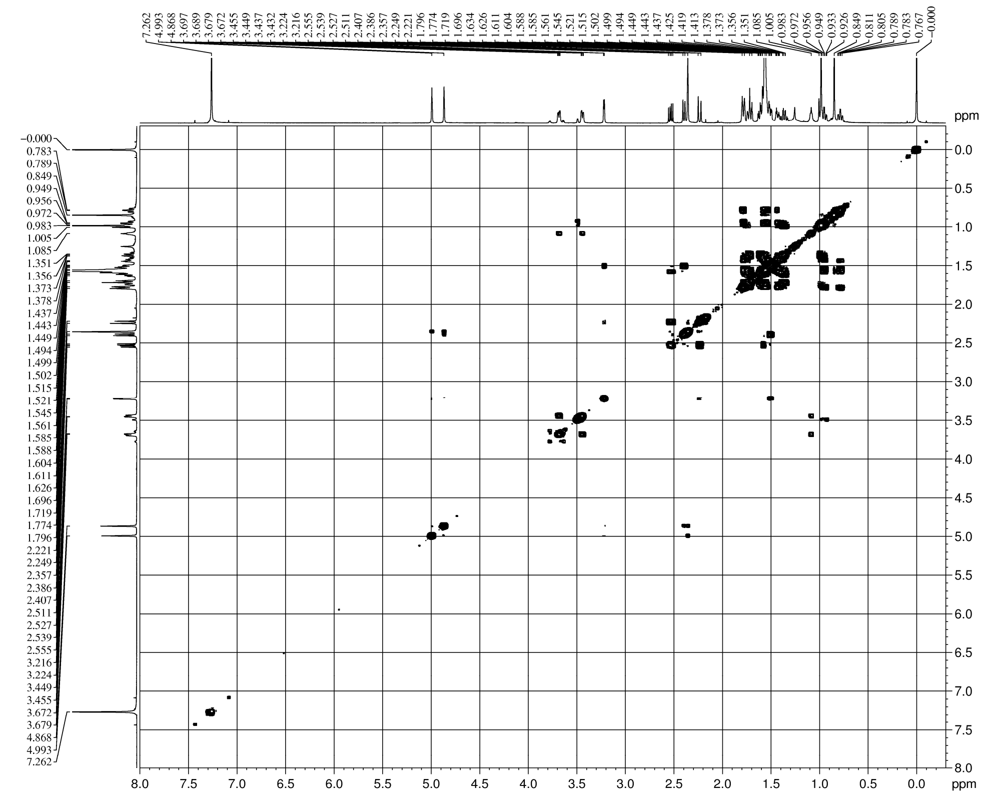

**Figure S117.** $^{1}H$–$^{1}H$ COSY spectrum of compound **12** in $CDCl_3$.

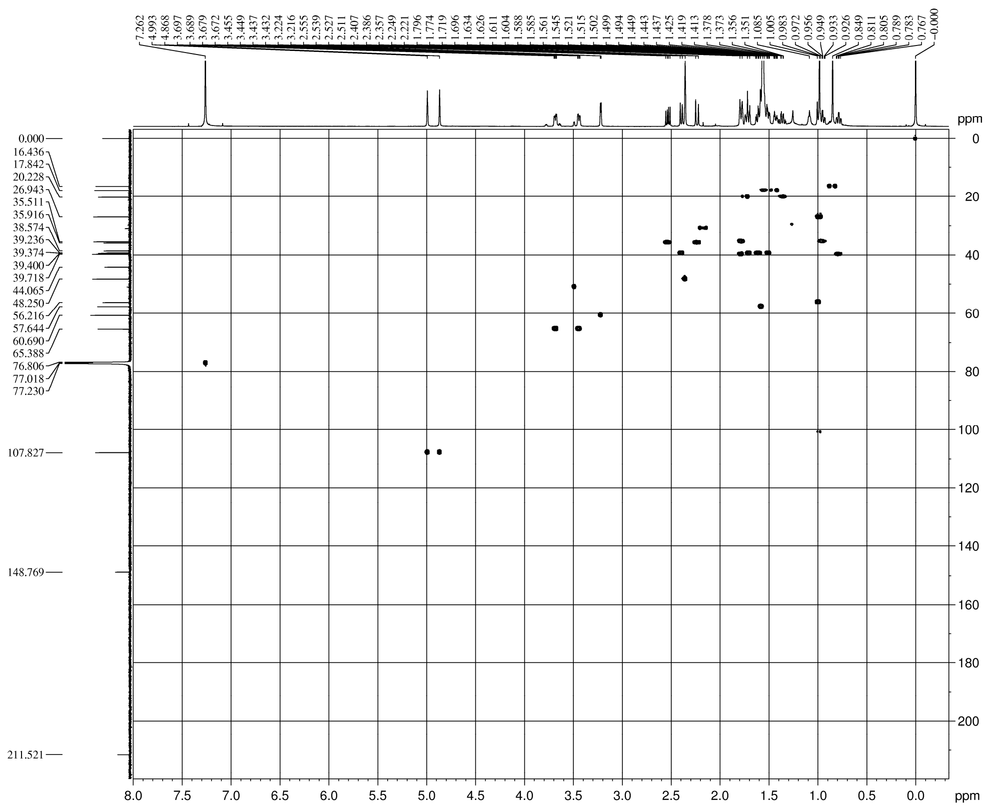

**Figure S118.** HSQC spectrum of compound **12** in $CDCl_3$.

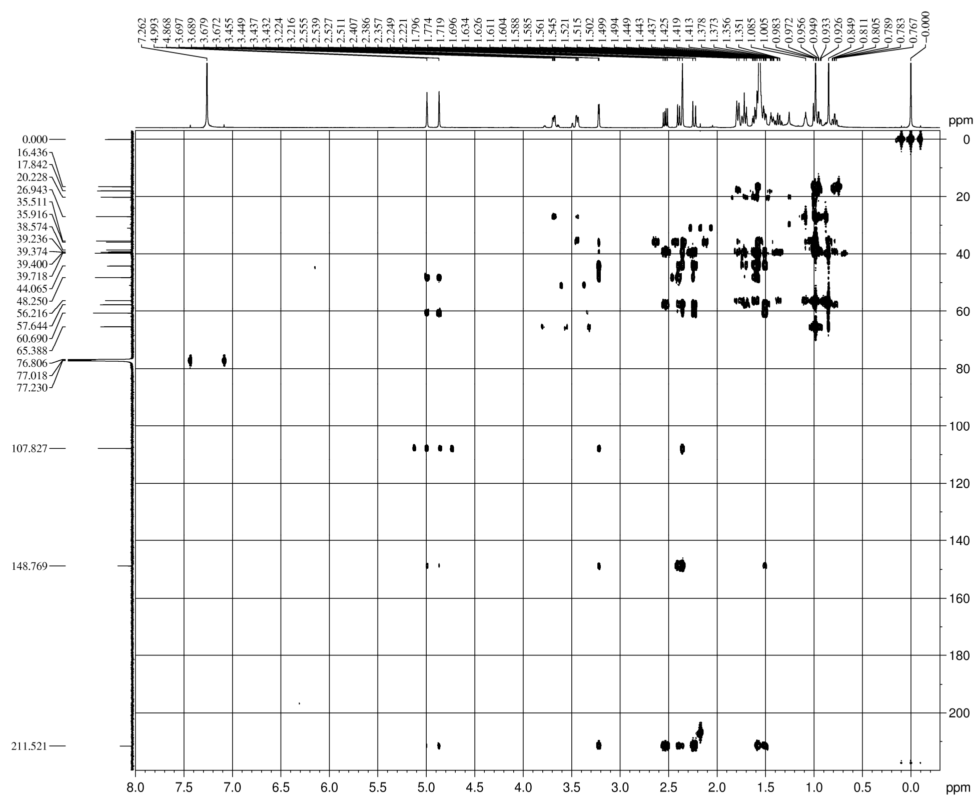

**Figure S119.** HMBC spectrum of compound **12** in $CDCl_3$.

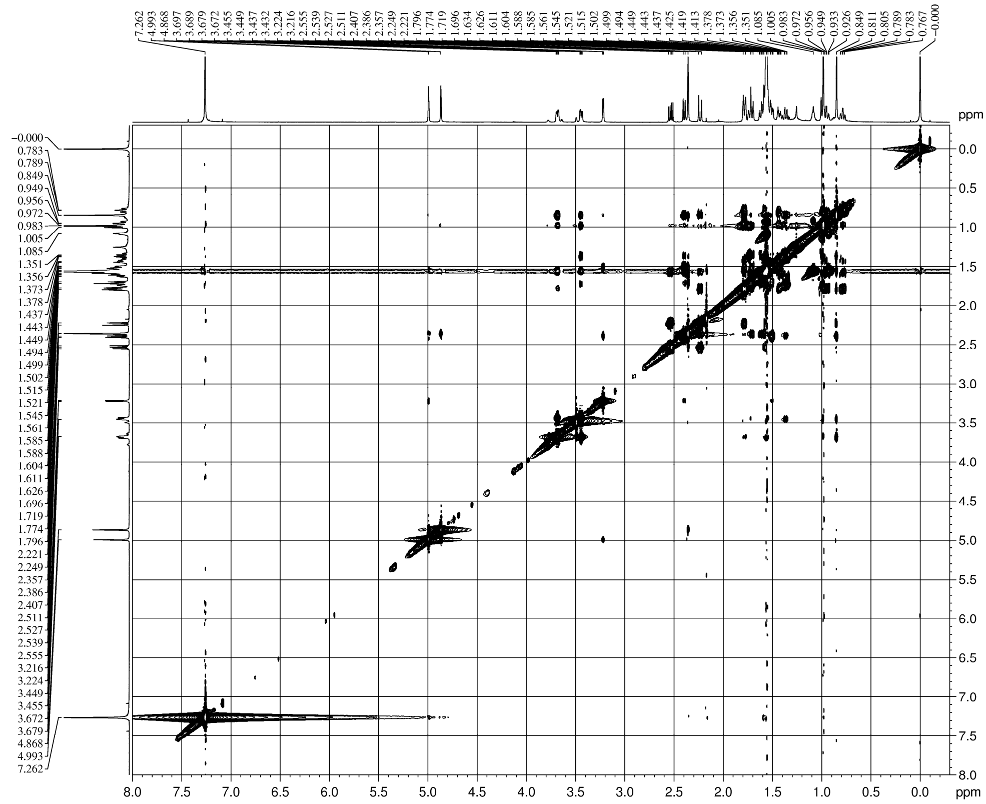

**Figure S120.** NOESY spectrum of compound **12** in $CDCl_3$.

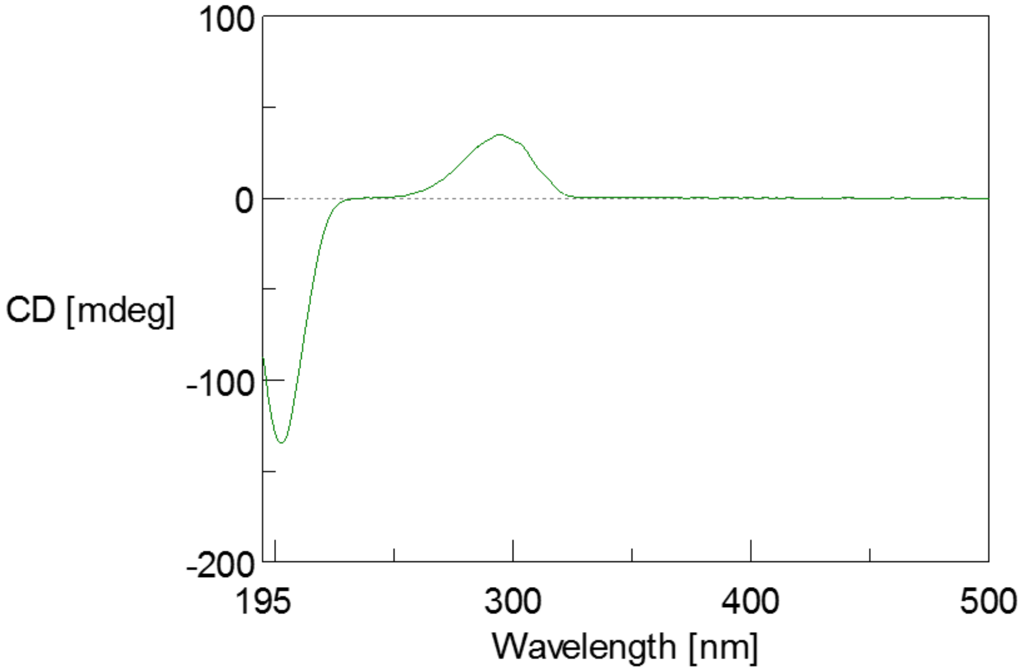


**Figure S121**. CD spectrum of compound **12** in MeOH.
